# Multiscale mathematical model-informed reinforcement learning optimizes combination treatment scheduling in glioblastoma evolution

**DOI:** 10.1126/sciadv.adv3316

**Published:** 2025-08-08

**Authors:** Zeming Liu, Ji Zhang, Liu Hong, Qing Nie, Xiaoqiang Sun

**Affiliations:** ^1^ School of Mathematics, Sun Yat-sen University, Guangzhou 510275, China.; ^2^ Department of Neurosurgery, State Key Laboratory of Oncology in South China, Collaborative Innovation Center for Cancer Medicine, Sun Yat-sen University Cancer Center, Guangzhou 510060, China.; ^3^ Department of Mathematics and Department of Developmental and Cell Biology, NSF-Simons Center for Multiscale Cell Fate Research, University of California Irvine, Irvine, CA 92697, USA.

## Abstract

Dynamic tumor-microenvironment interactions greatly affect growth and drug resistance, highlighting the importance and challenge of developing mathematical models to optimize treatment schedules. Here, we describe a multiscale mathematical model-informed reinforcement learning (M4RL) framework to simulate dynamic tumor-microenvironment interactions and optimize drug combination scheduling. We first develop a multiscale agent-based model (MSABM) for a critical biological scenario where interactions between tumor-associated macrophages (TAMs) and tumor cells (TCs) underlie immunotherapy resistance in glioblastoma. Next, we learn a surrogate model based on Fokker-Planck equations for the MSABM using a physics-informed neural network approach. We then design a surrogate model–based reinforcement learning method, using an efficient parallel actor-critic algorithm, to predict optimal scheduling of combination treatments. The most effective regimen of dynamic combination of CSF1R inhibitor (targeting TAMs) and IGF1R inhibitor (targeting TCs) is identified and then verified using spatial transcriptomic data. Overall, the M4RL framework introduces a computational approach for characterizing tumor-microenvironment interactions and optimizing dynamic scheduling of drug combinations.

## Teaser

Dynamic optimization of combination therapies is achieved by combining mathematical models and AI.

## INTRODUCTION

Glioblastoma, the most common and aggressive primary brain tumor in adults, is known for its rapid growth and extensive invasion of surrounding brain tissue, with macrophage polarization serving as an adverse prognostic indicator (*1*, *2*). Traditional treatments for glioblastoma, including surgery, radiotherapy, temozolomide chemotherapy, and targeted therapies against tumor cells (TCs), have limited efficacy (*3*). Therefore, designing more efficient therapeutic strategies remains critical for improving glioblastoma outcomes.

Recently, targeting the abundant tumor-associated macrophages (TAMs) (*4*) in the glioma microenvironment is becoming a promising immunotherapy strategy (*5*). TAMs can generally be divided into antitumorigenic (M1) macrophages and protumorigenic (M2) macrophages, which can polarize into each other within the tumor microenvironment (TME) (*6*). TCs secrete the cytokine colony-stimulating factor-1 (CSF1) (*7*), stimulating the polarization of more TAMs into M2 macrophages (*8*, *9*). By targeting the CSF1 receptor (CSF1R) on TAMs, the CSF1R inhibitor (CSF1R_I) blocks the CSF1-CSF1R interaction, promotes polarization toward the M1 phenotype, and enhances phagocytic capacity toward TCs (*10*). However, preclinical studies have shown that, despite a significant extension of the overall survival period after long-term CSF1R inhibition by the drug BLZ945, the recurrence rate of glioblastoma in mice remains higher than 50% (*7*). Mitigating the development of acquired resistance to CSF1R_I necessitates a deeper understanding of the intricate dynamics of interactions between the tumor and the TME.

Mathematical models can aid in quantitatively delineating the evolutionary dynamics of cancer drug resistance and in crafting more efficacious therapeutic strategies (*11*). Ordinary differential equations (ODEs) are frequently used to describe the evolution of TC population densities (*12*, *13*), especially in the context of drug resistance (*14*), as well as to investigate adaptive therapies (*15*–*17*) and combination therapies (*18*). Accounting for the spatial heterogeneity within tumor tissues and their microenvironments, partial differential equations (PDEs) can be effectively used for dynamic modeling that captures spatiotemporal evolution mechanisms and informs drug treatment strategies (*19*–*23*). In addition, stochastic differential equations (SDEs) can model the stochastic dynamics inherent in cancer processes, including cell division (*24*, *25*), cell migration (*26*), developmental trajectories (*27*), gene mutations (*28*, *29*), phenotypic heterogeneity, and drug resistance (*30*, *31*). Furthermore, to mimic tumor growth at individual cell level, cellular automata or agent-based models (ABMs) (*32*–*34*) have been developed to simulate cell behaviors, such as migration, proliferation, and death, as well as cell-cell interactions. Moreover, ABMs at the cellular level can be integrated with models at the TME scale and the molecular scale (*35*), forming a multiscale model to investigate the complex interactions between TCs and other TME components (*36*–*38*).

In particular, several mathematical modeling studies have been devoted to examine tumor-microenvironment interactions in glioblastoma (*38*–*43*). In our previous studies (*44*), we constructed a PDEs model to simulate the spatiotemporal dynamics of microenvironment-mediated resistance in glioma immunotherapy. This model characterizes the interactions between TCs and TAMs in glioblastoma, specifically those mediated by CSF1, IGF1, and interleukin-4 (IL-4), under treatment with the CSF1R inhibitor BLZ945. However, this model does not consider phenotypic heterogeneity and plasticity of TAMs nor molecular signaling regulations of TCs. In addition, we developed a multiscale ABM that incorporates cell dynamics, signaling pathways, gene mutations, and angiogenesis to investigate drug resistance to epidermal growth factor receptor (EGFR) inhibitors in glioblastoma (*41*).

Through the aforementioned modeling approaches, one can attempt to derive improved treatment regimens based on the tumor mechanisms and evolutionary characteristics demonstrated by the model (*45*). For instance, a constrained dynamic optimization problem has been formulated to design an adaptive treatment regimen for prostate cancer, where the optimal decision set in the optimization problem represents the treatment cycles and dosages of the adaptive therapy (*16*). Moreover, reinforcement learning (RL) has recently been used to optimize the adaptive therapy (*46*). Gallagher *et al.* (*47*) integrated dynamical modeling with RL to optimize adaptive drug scheduling, yielding better outcomes than existing adaptive protocols for prostate cancer. Nevertheless, these works establish the optimization of drug treatment on the basis of ODEs models of cell populations that do not account for spatial heterogeneity and stochasticity. In addition, these RL methods only optimize single drug scheduling, which cannot be simply extended to multidrug paradigm, since the latter requires finer mechanistic mathematical models. As mentioned above, ABM is good at simulating complex interactions between TCs and TME but has inherent shortcoming of high computational heavy. How to leverage the insights gained from the ABM simulations for efficient dynamic optimization of combination treatment is a long-standing challenging question.

In this study, we propose a multiscale mathematical model-informed reinforcement learning (M4RL) framework to simulate dynamic tumor-microenvironment interactions and further to optimize drug combination scheduling. We focus on glioblastoma to demonstrate the feasibility of the M4RL framework. We first develop a multiscale agent-based model (MSABM) to simulate glioma-macrophage interactions and resistance to immunotherapy with CSF1R_I treatment. This model incorporates diffusion of cytokines and drug molecules at the TME scale, interactions between TAMs and TCs at the cellular scale, and signaling pathways at molecular scale. Considering the considerable time cost associated with running the MSABM, which impedes the efficient exploration of optimal treatment regimens, we simplify the MSABM to a SDE-ODE hybrid model and mathematically derive the Fokker-Planck equations to characterize cell population dynamics for prediction and optimization purpose. Subsequently, we use a physics-informed neural network (PINN) to approximate a surrogate model, through combining the aforementioned Fokker-Planck equations as physical constraint and the output data from the MSABM under different combination treatments as data-driven constraint. Last, we use an efficient parallel actor-critic–based RL method to identify the most effective drug combination scheduling. Multiple sets of experimental data and spatial transcriptomic (ST) data are used to verify the predictions of MSABM, PINN, and M4RL.

## RESULTS

### Multiscale modeling of glioma-macrophage interactions

**Fig. 1.**
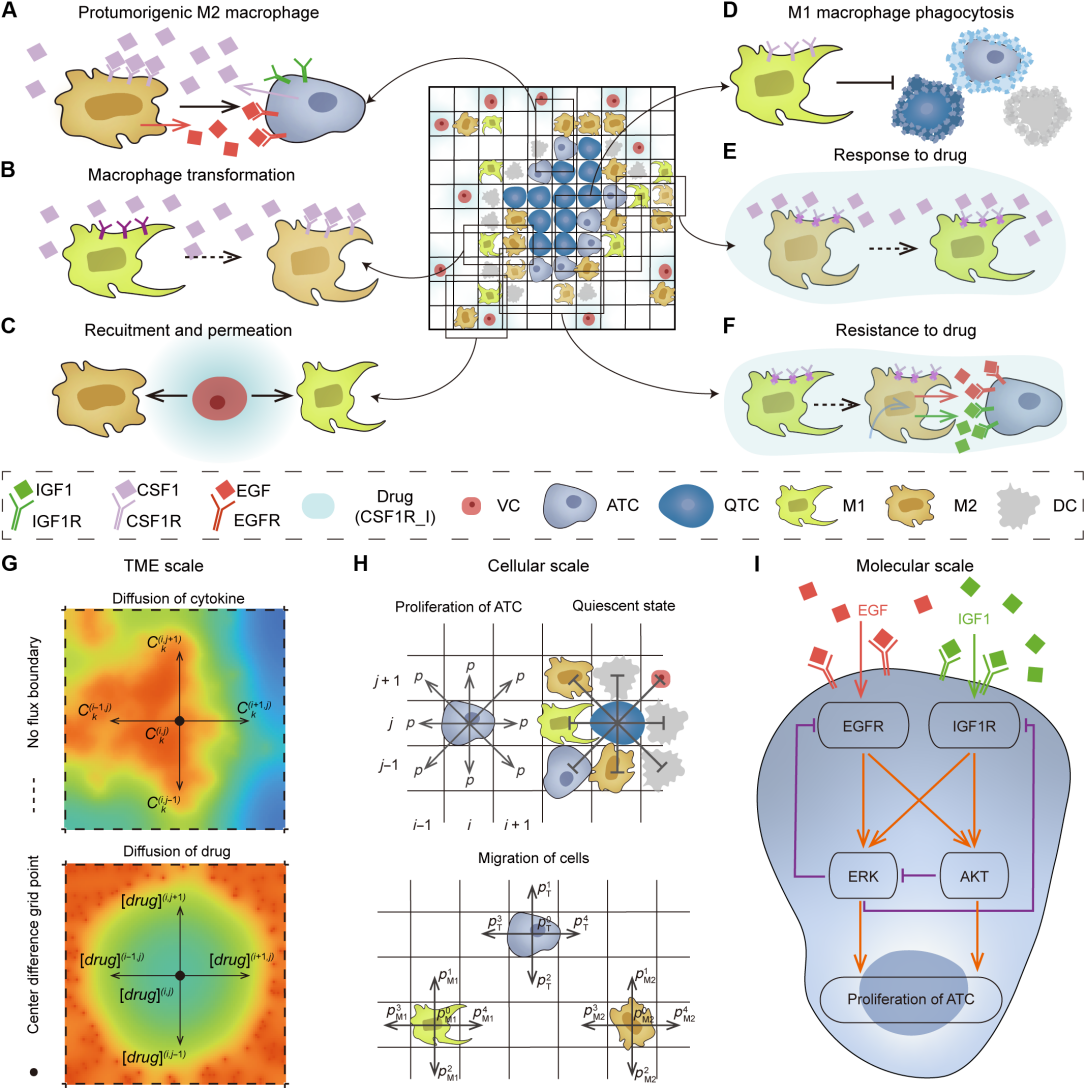
Schematic of MSABM. (**A**) TCs secrete CSF1, which can bind to CSF1R on TAMs, and M2 macrophages secrete EGF, thereby promoting TCs’ proliferation. (**B**) TCs secrete CSF1, which can bind to CSF1R on M1 macrophages, promoting their polarization into M2. (**C**) Macrophages are recruited to the TME through vascular cells and differentiate into M1 and M2 macrophages, while drug molecules also diffuse into the TME from the vascular cells. (**D**) M1 macrophages phagocytose TCs, inhibiting their proliferation, and can also phagocytose DCs. (**E**) CSF1R_I blocks the interaction between CSF1R and CSF1, leading to the polarization of a greater number of M2 macrophages toward the M1 phenotype. (**F**) Prolonged use of CSF1R_I induces the conversion of M1 macrophages into M2 phenotype, which then secrete IGF1, enhancing TCs’ proliferation. (**G**) Diffusion of cytokines and drug molecules in the TME. (**H**) The active and quiescent states of TCs, along with the migration of TAMs and TCs at cellular scale. (**I**) The molecular signaling pathways inside TCs. VC, vascular cell; QTC, quiescent TC.

We construct a MSABM of glioma TME to simulate the resistance of TCs to the CSF1R_I inhibition (Fig. 1). The model is based on mechanisms of the interactions between TCs and TAMs, mediated by the cytokines CSF1, epidermal growth factor (EGF), and IGF1, as well as the drug CSF1R_I. The model simulation is performed on a two-dimensional (2D) lattice representing a tissue slice, with one cell per lattice site. We establish three types of cell agents on a 2D lattice, including TCs, TAMs, and dead cells (DCs) that originate from TCs and TAMs after reaching their life span, along with vascular cells that are non-agent cells fixed on the lattice.

On the basis of the experimental mechanisms, the rules for agents in our MSABM is designed as follows (table S1). First, in the virtual TME, EGF secreted by M2 macrophages binds to the EGFR on TCs, promoting their proliferation (Fig. 1A); TCs can secrete CSF1, which binds to CSF1R on M1 macrophages, inducing the polarization of M1 into M2, thereby protecting TCs from being phagocytosed by M1 (Fig. 1B) (*8*, *9*, *48*, *49*). Undifferentiated (M0) macrophages are generally recruited into the TME through vascular cells, after which they differentiate into M1 macrophages and M2 macrophages (*6*, *50*). In addition, the drug factor CSF1R_I in the vasculature can infiltrate into the TME through diffusion (Fig. 1C). M1 macrophages in the TME phagocytose TCs and DCs, subsequently occupying their spaces (Fig. 1D) (*49*). Second, CSF1R_I blocks the binding of CSF1 with CSF1R on TAMs, inhibiting the polarization of M1 macrophages into M2, as such promoting the phagocytosis of TCs in the TME (Fig. 1E) (*10*). Third, prolonged use of CSF1R_I leads to the accumulation of other secreted cytokines, such as IL-4, in the TME. This accumulation induces a conversion of M1 macrophages into the M2 phenotype and promotes the secretion of IGF1 by M2 macrophages (*7*). The decrease in M1 macrophages and the increased secretion of IGF1, which binds to IGF1R on TCs, collectively contribute to the resistance of TCs to CSF1R_I (Fig. 1F) (*7*).

At the TME scale (Fig. 1G), we use a system of PDEs to describe the diffusion, secretion, and consumption of cytokines or drugs and numerically solved them using the finite difference method. At the cellular scale (Fig. 1H), apart from the interactions between the aforementioned cells, we designated TCs with available proliferation space (Moore neighborhood) as active TCs (ATCs), while those without proliferation space were classified as quiescent TCs; moreover, both TAMs and TCs can either remain stationary or relocate their available migration space (Von Neumann neighborhood), based on a calculated probability. While at the molecular scale (Fig. 1I), EGFR and IGF1R on TCs can bind with their respective ligands, EGF and IGF1 [both secreted by M2s, as previously described in Fig. 1 (B and F)], activating the downstream extracellular signal–regulated kinase (ERK) and protein kinase B (PKB or AKT) signaling pathways that regulates cell proliferation (*51*). A system of ODEs is used to describe this signaling network, with kinetic parameters estimated from time-course perturbation data using an improved genetic algorithm (fig. S1). More details of MSABM constructions can be found in text S1.

### Simulating tumor growth under treatment-naïve condition

**Fig. 2.**
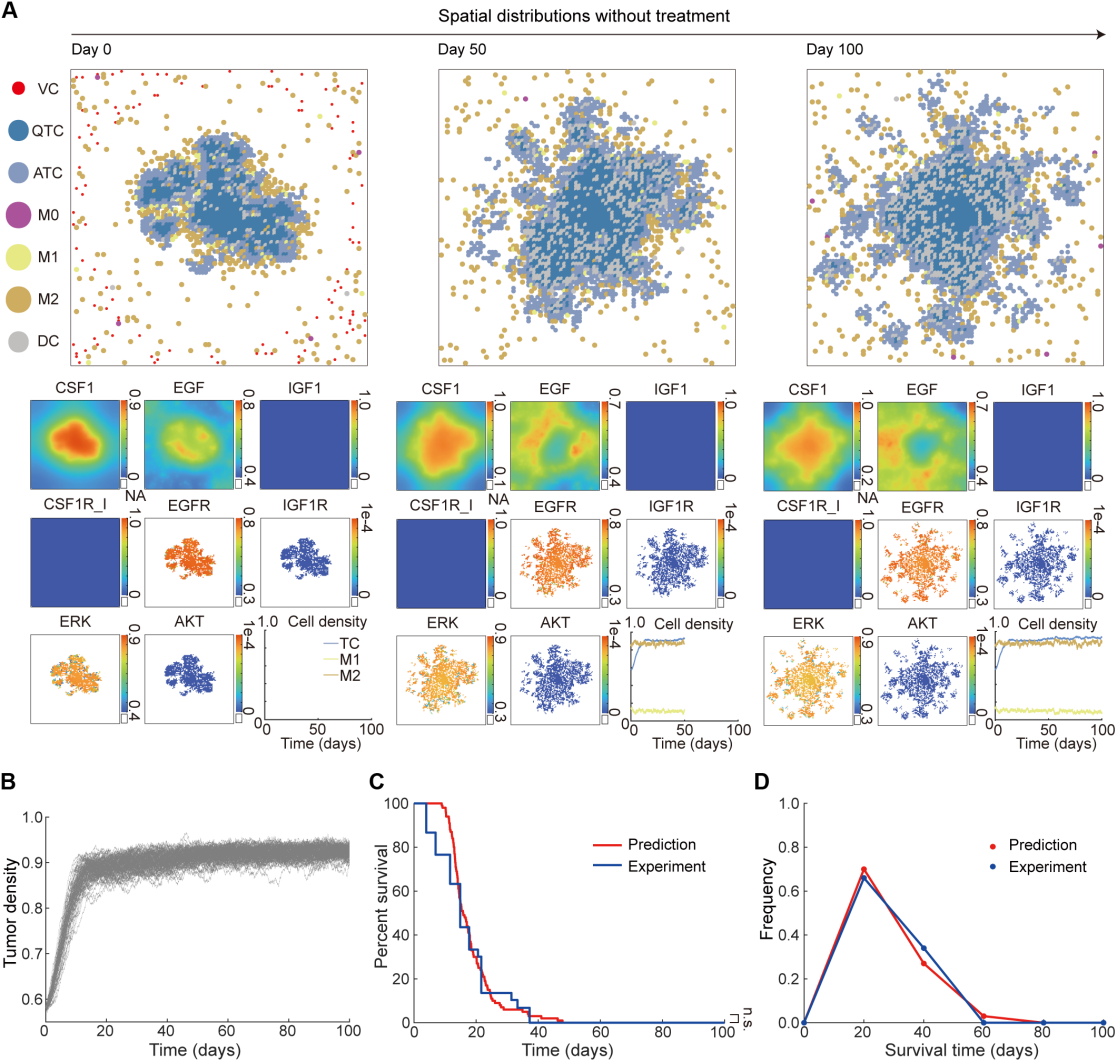
MSABM simulations and verification under the treatment-naïve condition. (**A**) The initial virtual TME (day 0) and single simulation slices of the MSABM at day 50 and day 100, along with the changes in cell density over time. (**B**) The evolution of TC density over time from multiple predictions (N = 100) without treatment. (**C**) Survival analysis from multiple predictions (N = 100), compared to the experimental data (*7*). (**D**) Survival time frequency from multiple predictions (N = 100) without treatment, compared to the experimental data (*7*). NA, not available; n.s., not significant.

We here demonstrate a typical simulation of tumor growth under treatment-naïve condition for up to 100 days, as shown in movies S1 and S2. In the initial virtual TME (Fig. 2A, day 0), there is a notable number of TCs at the center of the TME. Quiescent TCs occupy the interior, lacking proliferative space, while ATCs reside on the exterior, capable of proliferating outward. The copious secretion of CSF1 by the TCs results in a high CSF1 concentration (normalized, similarly applied to molecule concentration and cell density) at their aggregation sites within the TME (Fig. 2A, day 0, CSF1). This high concentration of CSF1 chemotactically attracts TAMs to these sites, leading to the formation of a dense layer of TAMs surrounding the TCs. Because of the absence of CSF1R_I (Fig. 2A, day 0, CSF1R_I), the abundant CSF1 binds extensively to the CSF1R on the TAMs, promoting the polarization of lots of M1 macrophages into M2 macrophages. Hence, the phagocytic activity of the few remaining M1 macrophages is insufficient to engulf the large number of TCs, in the treatment-naive case. In turn, the profuse M2 macrophages secrete EGF, increasing the EGF concentration in the TME (Fig. 2A, day 0, EGF). Without the influence of the drug CSF1R_I, the secretion of IGF1 by M2 macrophages is extremely low (Fig. 2A, day 0, IGF1). Furthermore, looking into the molecular signaling pathways in TCs depicted in Fig. 1I, the secreted EGF binds to and activates EGFR (Fig. 2A, day 0, EGFR), promoting downstream ERK activation (Fig. 2A, day 0, ERK). The elevated ERK exerts a negative feedback regulation on EGFR, maintaining EGFR activation at a relatively stable level. Simultaneously, because of the low level of IGF1 in the TME as well as the negative feedback regulation by ERK, both IGF1R (Fig. 2A, day 0, IGF1R) and its downstream AKT (Fig. 2A, day 0, AKT) remain at extremely low levels throughout the 100-day simulation, when treatment is absent.

Upon reaching the 50th day of simulation, we revisited the spatial distribution of cells, cytokines, and relevant proteins (Fig. 2A, day 50). It becomes evident that the cell aggregation formed by TCs, TAMs, and DCs has substantially enlarged, with peripheries exhibiting a petal-like pattern. This observation is attributed to the proliferation of ATCs into surrounding tissue. Moreover, the increased number of interspersed DCs is due to the scarcity of phagocytic M1 macrophages, coupled with the limited removal rate of DCs within the TME. According to the time course of cell density (Fig. 2A, day 50, cell density), we observe that most TAMs remain in the M2 state, with their density stabilizing around 0.85, while M1 macrophages account for only approximately 0.15. As for TCs, their density initially rises sharply from approximately 0.58 to nearly 0.9 within the first 20 days and then decelerates because of the constraints imposed by the maximum carrying capacity of the TME. As the simulation progresses to day 100, we observe the emergence of numerous small cell aggregations surrounding the central, larger aggregation, potentially leading to tumor metastasis (Fig. 2A, day 100).

The above simulations are consistent with current understanding and experimental data on glioma growth, demonstrating that our model can faithfully recapitulate the tumor growth dynamics under treatment-naïve condition. Furthermore, to mimic intertumor heterogeneity, we firstly selected three parameters (i.e., $\alpha_{\text{ERK}},\alpha_{C},$ and $\alpha_{I}$, which are related to the tumor proliferation, TAM polarization, and CSF1R_I responsiveness, respectively) in the MSABM. We generated three normal distributions with these parameters’ basal value as the means and 0.1 times the basal value as the variance. We then conducted multiple predictions (*N* = 100) with the same initial virtual TME, which includes the spatial distributions of cells, cytokines, and drugs, along with the intracellular protein concentrations (as shown in Fig. 2A, day 0). In each prediction, the values of the three selected parameters were randomly sampled from their respective normal distributions, while all other parameters were set at their basal values. Details of the multiple predictions are mentioned in Materials and Methods. Subsequently, we calculated survival time for each individual based on the evolution of TC density (Fig. 2B). Here, we assumed that an individual is considered dead once the TC density within the TME reaches a predefined, fixed threshold. The time at which this occurs is defined as the individual’s survival time. In addition, the Kaplan-Meier (KM) curve was calculated using the equation described in Materials and Methods. Figure 2C shows that the KM curve aligns well with that from the experimental data, which is also represented as a KM curve derive from vehicle-treated mice (*N* = 30) bearing high-grade glioma tumors (*7*). Also, the predicted survival time frequency closely matches the experimental results (Fig. 2D) (*7*).

### Simulating drug resistance to CSF1R_I treatment

**Fig. 3.**
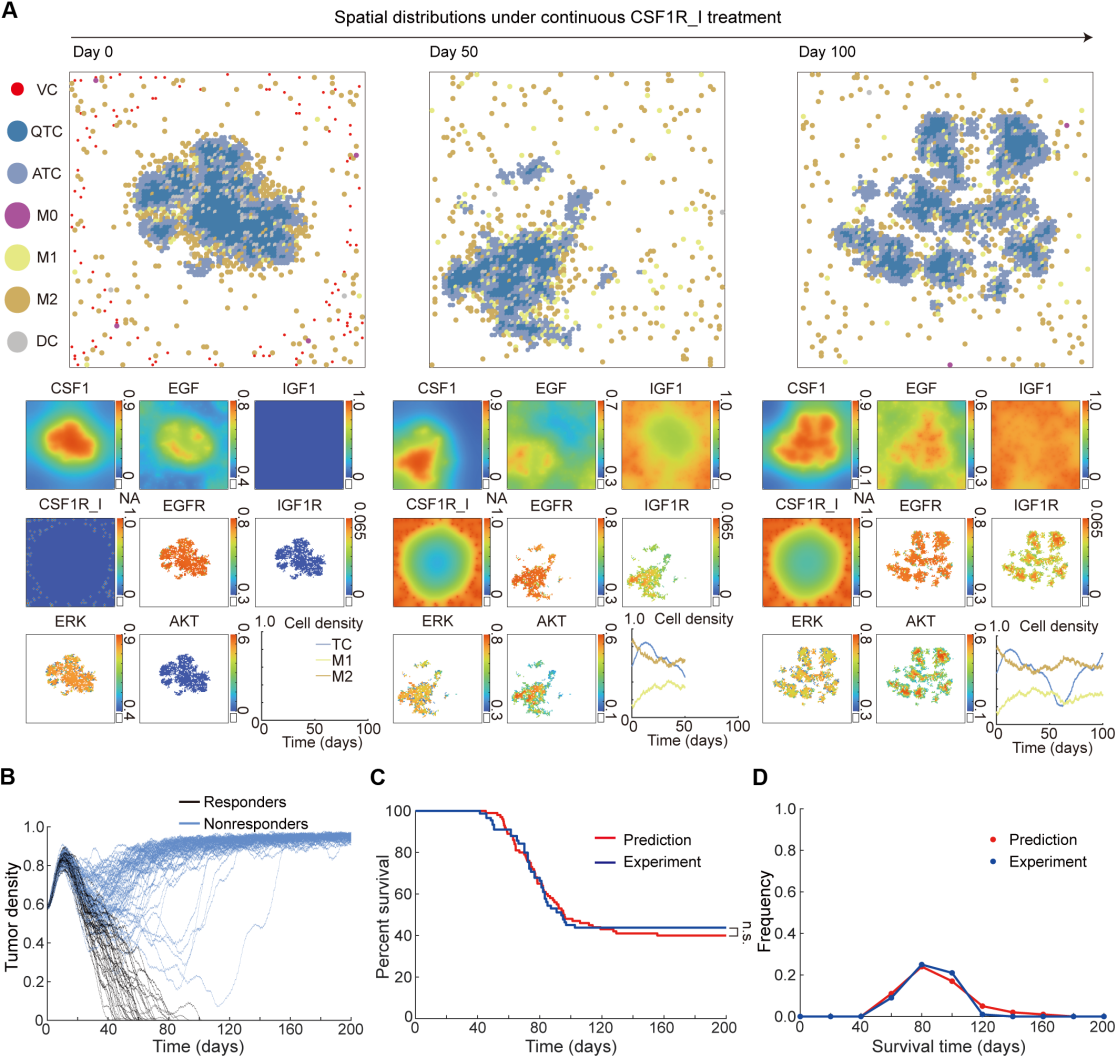
MSABM simulation and verification under the continuous-CSF1R_I-treatment condition. (**A**) The initial virtual TME (day 0) and single simulation slices of the MSABM with continuous CSF1R_I treatment at day 50 and day 100, along with the changes in cell density over time. (**B**) The evolution of TC density over time from multiple predictions (N = 100) with continuous CSF1R_I treatment. (**C**) Survival analysis from multiple predictions (N = 100) with continuous CSF1R_I treatment, compared to experimental data (*7*). (**D**) Survival time frequency from multiple predictions (N = 100) with continuous CSF1R_I treatment, compared to experimental data (*7*).

In the case of simulating continuous CSF1R_I treatment, we kept the initial virtual TME (Figs. 2A, day 0 and 3A, day 0), the number of predictions (*N* = 100), and the sampling strategy for the three parameters unchanged, but the maximum simulation and prediction duration were extended to 200 days. First, we examine the spatial distribution in a single simulation. Unlike the treatment-naive case above (Fig. 2A, day 50, CSF1R_I), CSF1R_I permeates into the TME through the peripheral vasculature (Fig. 3A, day 50, CSF1R_I) continuously. These CSF1R_I molecules initially blocks the binding of CSF1 to CSF1R on M2 macrophages, leading some M2 macrophages to polarize into M1 macrophages (Fig. 3A, day 50). Consequently, the density of M1 macrophages increases, reaching a maximum of approximately 0.4 (Fig. 3A, day 50, cell density). The increase in the number of M1 macrophages leads to the phagocytosis of ATCs on the outer layers of the cell aggregation, thereby allowing the internal quiescent TCs to gain proliferative space and convert back into ATCs. As a result, the overall number of TCs substantially decreases. However, since the distribution of M1 macrophages around the TCs is uneven, phagocytosis may cause the cell aggregation to migrate. Accordingly, the migration of TCs results in the change of spatial distribution of CSF1 in the TME (Fig. 3A, day 50, CSF1). Because of the chemotaxis toward CSF1, TAMs continue to aggregate around the TCs.

In addition to affecting the polarization of TAMs, the accumulation of CSF1R_I also promotes the secretion of large amounts of IGF1 by M2 macrophages. Thus, although both EGF and IGF1 are secreted by M2 macrophages, they have different concentration distributions within the TME. The spatial distribution of EGF and IGF1 closely corresponds to the M2 macrophage aggregation (Fig. 3A, day 50, EGF and IGF1). Because of the chemotactic movement of M2 macrophages toward high CSF1 concentration, the concentration of EGF is also elevated around TCs. As a result, the concentrations of EGFR and its downstream kinase protein ERK (Fig. 3A, day 50, ERK) are similar to those in the no-treatment case. However, two key differences arise: First, the secretion of IGF1 leads to an increase in IGF1R and AKT concentrations within TCs; second, the spatial distribution of IGF1, with higher concentrations at the periphery and lower concentrations in the center of the TME, results in spatial variations in the concentrations of IGF1R (Fig. 3A, day 50, IGF1R) and its downstream protein AKT (Fig. 3A, day 50, AKT) within TCs.

As the duration of continuous CSF1R_I treatment extends to 100 days, the accumulation of CSF1R_I accelerates the polarization of M1 macrophages into M2 macrophages, with the secretion rate of IGF1 also increasing in response to the accumulating CSF1R_I (Fig. 3A, day 100). While the concentration of ERK within TCs remains relatively stable at a high level (Fig. 3A, day 100, ERK), the increased AKT concentration at a moderate level (Fig. 3A, day 100, AKT) promotes the proliferation of TCs. Simultaneously with the decrease in the number of phagocytic M1 macrophages, this ultimately leads to tumor resistance to CSF1R_I (Fig. 3A, day 100, cell density). After tumor recurrence, we observed that the TCs, due to the phagocytic activity of M1 macrophages, do not form highly concentrated cell aggregations but instead appear as more dispersed cell clusters in smaller size. Furthermore, the phagocytic effect of M1 macrophages on DCs is clearly noticeable; compared to the no-treatment case (Fig. 2A), almost no DCs are present in the continuous-CSF1R_I-treatment case (Fig. 3A).

The MSABM multiple predictions (*N* = 100) under continuous CSF1R_I treatment demonstrate that the population includes both responders and nonresponders to CSF1R_I (Fig. 3B). It shows that responders can be cured after 40 to 80 days of continuous CSF1R_I treatment (movie S3), while nonresponders initially experience a reduction in TCs during the early stages of the treatment, followed by a gradual increase, ultimately leading to the tumor relapse (movie S4). These predictions show good agreement with the experimental studies [figure 1D in (*7*)].

To further quantitatively verify the model predictions, we performed survival analysis for the simulated virtual cohort based on multiple predictions. The predicted survival curve over 200 days showed no significant difference with the experimental observations after conducting a log-rank test (Fig. 3C). The experimental observation here is represented as a KM curve of BLZ945-treated mice (*N* = 90) bearing high-grade glioma tumors (*7*). In addition, the predicted survival time distribution aligns well with the experimental one (Fig. 3D). The above results indicate that continuous treatment with CSF1R_I alone is likely to result in tumor resistance and can only maintain the survival of ~40% of the population, necessitating the designing and optimization of combination treatments with reduced dosage of CSF1R_I to mitigate the resistance.

### Sensitivity analysis of key parameters

**Fig. 4.**
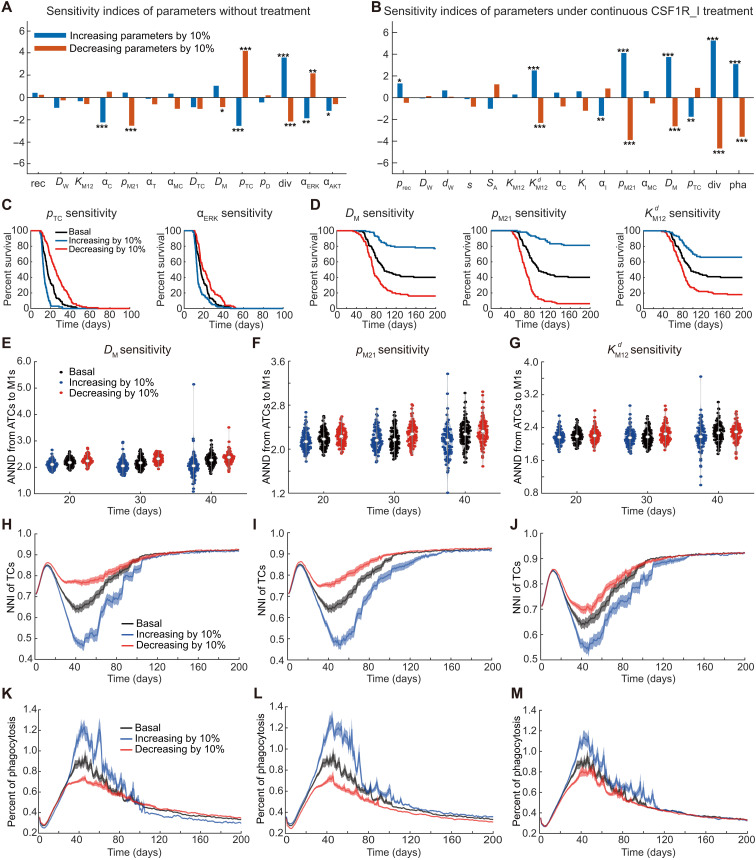
Sensitivity analysis of model parameters. (**A**) Parameter sensitivity analysis in the treatment-naïve case. Sensitive parameters are closely related to the proliferation of TCs and the phagocytosis of M1 macrophages. $p_{\text{TC}},div,\alpha_{\text{ERK}},\text{and}\;\alpha_{\text{AKT}}$ are key parameters related to TC proliferation; $\alpha_{C}\;\text{and}\;p_{M21}$ directly influence the number of M1 macrophages; $D_{M}$ stands for the migration rate of TAMs. ***P < 0.01, **P < 0.05, and *P < 0.1 for log-rank test. (**B**) Parameter sensitivity analysis in the continuous-CSF1R_I-treatment case. Apart from the aforementioned parameters related to TC proliferation, parameters associated with TAM migration and polarization, such as $K_{M12}^{d}$, $p_{M21}$, $D_{M}$, and pha, have a more pronounced impact on the model in this case. (**C**) Survival curves predicted by the model with varying values of $p_{\text{TC}}$ or $\alpha_{\text{ERK}}$ under the treatment-naïve condition. (**D**) Survival curves predicted by the model with varying values of $D_{M}$, $p_{M21}$, and $\;K_{M12}^{d}$ in the continuous-CSF1R_I-treatment case. (**E** to **G**) Violin plots of the ANND from ATCs to M1 macrophages at day 20, day 30, and day 40 as $D_{M}$, $p_{M21}$, and $K_{M12}^{d}$ change. We selected these time points because the period from 20 to 40 days is a critical phase during which difference between responders and nonresponders to CSF1R_I begins to emerge, and after 40 days, the TCs in some individuals (Fig. 3B) which are responders to CSF1R_I have been almost completely phagocytosed. (**H** to **J**) The mean NNI (with 0.1 × SD) of TCs across the predicted population when $D_{M}$, $p_{M21}$, and $K_{M12}^{d}$ change. (**K** to **M**) The mean phagocytosis rate (with 0.1 × SD) of M1 macrophages across the predicted population when $D_{M}$, $p_{M21}$, and $K_{M12}^{d}$ change.

For the multiple predictions (*N* = 100) in the treatment-naïve case and the continuous-CSF1R_I-treatment case, we separately selected certain parameters from the parameter space of the MSABM for sensitivity analysis (Fig. 4). We increased or decreased each selected parameter by 10% from its baseline value, and the resulting model outcomes were used to calculate the sensitivity indices of the selected parameters using the formulation described in Materials and Methods. A larger sensitivity index indicates a greater positive impact on individual survival. The parameters closely related to TC proliferation, $p_{\text{TC}}$ and div, represent the basal proliferation probability of TCs and the cell cycle of TCs, respectively, and show significant effects in both the treatment-naïve case (Fig. 4A) and the continuous-CSF1R_I-treatment case (Fig. 4B). Meanwhile, we found that in Fig. 4A, increasing $\alpha_{C}$ (the adjustment coefficient of the CSF1-related Hill function) can promote the polarization of M1s to M2s, thereby markedly enhancing TC proliferation. In contrast, decreasing $\alpha_{C}$ does not lead to a pronounced inhibitory effect on tumor growth, suggesting that controlling CSF1 alone is insufficient to transform enough M2s into M1s to effectively suppress tumor progression. Moreover, an increase in $p_{\text{TC}}$ accelerates TC proliferation, leading to a decrease in the survival rate of the predicted population (Fig. 4C, $p_{\text{TC}}$ sensitivity). Conversely, an increase in div represents a longer TC cycle in the simulation, delaying tumor formation and extending the survival time of individuals. $\alpha_{\text{ERK}}\;$ and $\alpha_{\text{AKT}}$ represent regulatory parameters related to ERK and AKT in signaling pathways of TCs. A decrease in either $\alpha_{\text{ERK}}\;$ or $\alpha_{\text{AKT}}$ could slow down tumor proliferation, thereby extending the survival time (Fig. 4C, $\alpha_{\text{ERK}}$ sensitivity). In addition, pha and $p_{\text{rec}}$ represent the basal phagocytosis rate of M1s and the basal recruitment probability of TAMs, respectively. The significance of these two parameters highlights the critical role of TAMs during CSF1R_I treatment (Fig. 4B).

In the no-treatment case, the impact of $D_{M}$ (migration coefficient of TAMs) is minimal (Fig. 4), while in the continuous-CSF1R_I-treatment case, $D_{M}$ exhibits high significance (Fig. 4B). This suggests that the increase in $D_{M}$ effectively enhances M1 macrophages’ phagocytosis toward TCs only when a sufficient number of M1 macrophages are present in TME as a result of CSF1R_I treatment. Conversely, a reduction in $D_{M}$ markedly decreases the survival rate of the population in multiple predictions (Fig. 4D, $D_{M}$ sensitivity). A decrease in $p_{\text{M21}}$ inhibits the polarization of M2 macrophages into M1 macrophages in the TME, thereby suppressing phagocytosis and reducing the survival time of individuals. However, increasing $p_{\text{M21}}$ in the no-treatment case has a little effect on the survival curve, whereas in the continuous-CSF1R_I-treatment case, increasing $p_{\text{M21}}$ notably enhances individuals’ survival (Fig. 4D, $p_{\text{M21}}$ sensitivity). This indicates that, although the polarization from M2 macrophages to M1 macrophages occurs naturally in the TME, the CSF1R_I treatment is necessary for this process to exert a more pronounced effect. $\;K_{\text{M12}}^{d}$ reflects the responsiveness of TAMs to the CSF1R_I factor. CSF1R_I permeates from vascular cells into the TME, with diffusion being continuous. The larger the value of $K_{\text{M12}}^{d}$, the stronger inhibition of CSF1R on the TAMS by CSF1R_I, resulting in a marked improvement in individuals’ survival. Conversely, a lower value of $K_{\text{M12}}^{d}$ indicates that TAMs are less responsive to CSF1R_I, leading to a decrease in the predicted population survival rate (Fig. 4D, $K_{\text{M12}}^{d}$ sensitivity).

Together, sensitivity analyses of parameters $D_{M}$ and $K_{\text{M12}}^{d}$ at the TME scale, $p_{\text{TC}}$ and $p_{M21}$ at the cellular scale, as well as $\alpha_{\text{ERK}}$ at molecular scale emphasize the need for in-depth investigation of tumor evolution and drug treatment, necessitating a multiscale approach. The above results also suggest that intervention strategies, including increasing CSF1R_I effectiveness on TAMs, accelerating the TAMs’ migration rate, or suppressing ERK and AKT signaling in TCs, might enhance the CSF1R_I treatment effect.

We further conducted a more in-depth quantitative analysis of $D_{M}$*,* $p_{M21}$, and $\;K_{\text{M12}}^{d}$ in the continuous-CSF1R_I-treatment case (Fig. 4, E to M). To this end, we defined the average nearest-neighbor distance (ANND) from ATCs to M1 macrophages, the nearest-neighbor index (NNI) of TCs, and the percent of phagocytosis (see details in Materials and Methods). We found that adjusting $D_{M}$*,* $p_{\text{M21}}$, and $\;K_{\text{M12}}^{d}$ by increasing or decreasing their values by 10% essentially affects the ANND from ATCs to M1 macrophages (Fig. 4, E to G). Comparative analysis reveals that, after 20 to 40 days of continuous CSF1R_I treatment, increasing these three parameters results in a relative decrease in the ANND from ATCs to M1 macrophages, while the opposite occurs when these parameters are decreased. These occur as increasing $D_{M}$ enhances TAM migration, reducing the ANND from the ATCs on the periphery of the central TC aggregation to M1 macrophages, making ATCs more likely to be phagocytosed. Also, increasing $p_{M21}$ raises the polarization rate of M2 macrophages to M1 macrophages, resulting in a greater number of M1 macrophages around the TCs, thus decreasing the ANND from ATCs to M1 macrophages. Similarly, increasing $K_{\text{M12}}^{d}$ enhances the responsiveness of TAMs to CSF1R_I and then promotes the polarization of more M2 macrophages into M1 phenotype, leading to a slight reduction in the ANND from ATCs to M1 macrophages.

Furthermore, we evaluated NNI that reflects the degree of cell aggregation, with lower values indicating greater clustering of TCs. Consistent with the above analysis, increasing $D_{M}$*,* $p_{M21}$, and $\;K_{M12}^{d}$, leads to enhanced M1 phagocytosis due to the decrease in ANND from ATCs to M1 macrophages in the period between day 20 and day 40. As a result, the small cell clusters on the periphery of the central TC aggregation are initially phagocytosed, leading to increased clustering of TCs while reducing overall TC density (Fig. 4, H to J). Consistently, the impact of the above three parameters on the phagocytosis rate of M1 macrophages on TCs were also analyzed (Fig. 4, K to M). In addition, the average nearest-neighbor index and Moran index were used to further assess the impact of $D_{M}$ on cell distributions (fig. S2). Together, the above results demonstrate how the three significant sensitive parameters influence the ANND from ATCs to M1 macrophages and thus the M1 phagocytosis and the aggregation of TCs, explaining the changes in the predicted survival curves observed in Fig. 4D.

### Investigating role of quiescent TCs in drug resistance

**Fig. 5.**
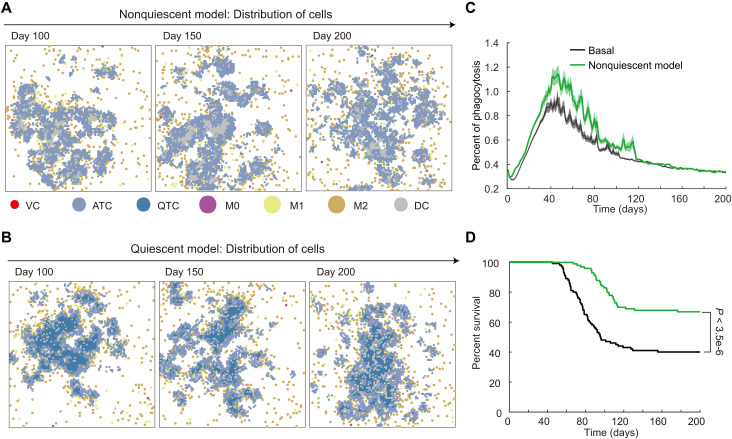
Comparison of basal MSABM with nonquiescent model under continuous CSF1R_I treatment. (**A**) The spatial distributions of cells for a nonresponder at day 100, day 150, and day 200 in the nonquiescent model. It shows that, in the absence of quiescent TCs, a larger number of DCs appear within the TC aggregation. (**B**) The cell distributions for a nonresponder at day 100, day 150, and day 200 in the quiescent model (i.e., basal MSABM). (**C**) The mean phagocytosis rate, with 0.1 × SD, of M1 macrophages across the predicted population under nonquiescent model versus the basal MSABM. (**D**) Survival curves predicted by the nonquiescent model versus the baseline MSABM. Log-rank test *P* value was used to assess the statistical significance of difference between the two survival curves.

In our MSABM model, TCs are divided into two categories. One type is ATCs, which have proliferative space and undergo division once per cell cycle based on a calculated probability, with their age increasing over time in the simulation. When an ATC attempts to divide but its Moore neighborhood is fully occupied, it transitions into a quiescent state, with its age ceasing to increase. Once proliferative space becomes available again, it reverts to the active state and immediately proliferate. To investigate the role of TC quiescence in tumor resistance under CSF1R_I treatment, we defined a nonquiescent model, in which TCs exist only in the active state. When a TC’s Moore neighborhood is fully occupied in the nonquiescent model, it cannot proliferate and proceeds to the next cell cycle phase, but its age continues to increase. Then, we compared the basal MSABM model with the nonquiescent model under continuous CSF1R_I treatment (Fig. 5). We investigated the cell distributions at day 100, day 150, and day 200 in the individual simulation of a nonresponder, as shown in movie S5. In the nonquiescent model, we observed a large number of DCs at the center of the TC aggregation, caused by the natural death of central ATCs that had reached the end of their life span. In addition, the TCs were more dispersed, allowing TAMs to easily infiltrate the TC aggregations (Fig. 5A). While in the basal model, the center of the TC aggregation is occupied by quiescent TCs with arrested cell cycles (*52*). As a result, there are fewer DCs inside the tumor, with smaller clusters at the periphery. Hence, TAMs encounter increased difficulty in entering the TC aggregations (Fig. 5B). Therefore, the more dispersed TCs in the nonquiescent case leads to more active phagocytic activity between M1s and TCs, which causes the TCs at the periphery to be more easily phagocytosed, as reflected by the higher average phagocytosis rate in the nonquiescent model (Fig. 5C). Consequently, the nonquiescent model predicts a higher survival curve (Fig. 5D), which, however, violates the experimental data (*7*) as analyzed in Fig. 3C.

The above analysis uncovers a substantial role of quiescent state of TCs in contributing to tumor resistance against CSF1R_I treatment. That is, the quiescent state of TCs preserves the proliferative potential of TCs while maintaining the stability of the large central cell aggregation in terms of spatial distribution, rather than having the aggregation break into smaller clusters due to natural cell death, as shown in movies S5 and S6. This prevents TAMs from migrating into the tumor interior, thereby protecting the TCs from extensive phagocytosis by M1s, ultimately promoting the development of tumor resistance under the continuous-CSF1R_I treatment (Fig. 5D).

Summarizing the above results, our computational analysis (Figs. 3 and 4) justifies that IGF1-mediated effects on both TAMs and TCs are crucial for tumor resistance to CSF1R_I treatment, aligning with the experimental findings (*7*). Furthermore, our additional investigation (Fig. 5) reveals that the quiescent state of TCs not only shields them from M1-mediated phagocytosis but also preserves their proliferative potential under high levels of IGF1, both of which contribute to the development of tumor resistance. Given this, exploring a combination treatment strategy that combines CSF1R_I (targeting TAMs) with drugs targeting TCs (such as IGF1R_I) holds considerable promise. These analyses collectively underscore the importance of further investigating the optimal combination scheduling of CSF1R_I and IGF1R_I, which we will delve into in the following sections.

### Development of a M4RL framework

**Fig. 6.**
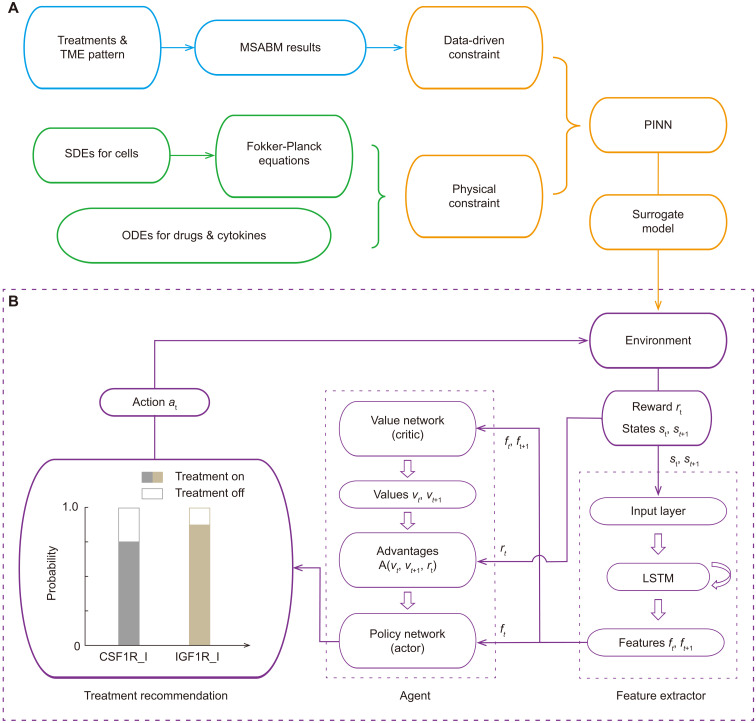
Flowchart of M4RL framework. (**A**) Diagram illustrating the process for learning the surrogate model from the MSABM. (**B**) Schematic of the surrogate model–based RL.

Our MSABM adopts a bottom-up approach to reconstructing the spatiotemporal dynamics of tumor growth and drug resistance by integrating the molecular scale, cellular scale, and TME scale mechanisms. However, the high time cost of the MSABM and the substantial resources required for multiple predictions (approximately 550 core hours for a full 200-day multiple predictions with *N* = 100 and simulation time interval = 0.5 hours) make its application in clinical settings challenging. To enhance the applicability of the MSABM for optimizing drug treatment scheduling, we develop a M4RL framework (Fig. 6) by introducing a surrogate model. As detailed in Materials and Methods below, the surrogate model is driven by simulated data from the MSABM under various drug combination treatments and is physically constrained by a hybrid model composed of SDEs for describing cell densities and ODEs for describing cytokine and drug concentrations. Furthermore, we transform each SDE into a Fokker-Planck equation to capture the evolution of the probability distribution of cell density across a predicted population over time from a more macroscopic perspective. Last, we use PINN to approximate the Fokker-Planck equation model, resulting in the surrogate model (Fig. 6A). Specifically, the surrogate model is a neural network approximation of the Fokker-Planck equations, representing the temporal evolution of the probability density function (p.d.f.) for TC density within the population, with a specific treatment and initial conditions as the input. The resulting surrogate model substantially reduces the computing cost for large-scale predictions (less than 18 core hours for a 200-day prediction with simulation time interval = 0.5 hours), providing an efficient and reliable tool for subsequent use in RL to optimize treatment scheduling (i.e., dosage, timing, and combination).

Next, we combine the trained surrogate model with the asynchronous advantage actor–critic (A3C) algorithm (*53*), a RL method, to identify the optimal scheduling of combination treatment. Consistent with the experimental design (*7*), we combine CSF1R_I with IGF1R_I to reduce glioma resistance to CSF1R_I (Fig. 3, B and C), mitigate the inevitable negative impact of the TCs’ quiescent state on CSF1R_I treatment (Fig. 5D), and minimize drug usage. During RL training, the environment guided by the surrogate model provides a time series composed of two dimensions—current predicted population survival probability and the change in survival probability—which are used as the state, while the reward is calculated on the basis of the reward function (algorithm S2). The state is then passed through a long short-term memory model for feature extraction, which outputs a 4D feature vector. This feature vector, along with the reward, is input into the agent of RL for value assessment and advantage calculation, thereby assisting in policy decisions for two drugs (CSF1R_I and IGF1R_I). Last, we apply a SoftMax function to convert the policy output into probabilities, and the next action at each time step is recommended probabilistically (Fig. 6B). Further details can be found in Materials and Methods. The pseudocode and relevant parameters for the surrogate model–based A3C network are provided in algorithm S1 and table S5, and those for the reward function in RL are provided in algorithm S2 and table S6. The whole M4RL framework (including the MSABM simulations, the surrogate model training, and the RL training) was performed on a server equipped with two Intel Xeon Platinum 8280 central processing units (CPUs) at 2.70 GHz, offering a total of 112 logical cores.

### Learning and verification of the surrogate model

**Fig. 7.**
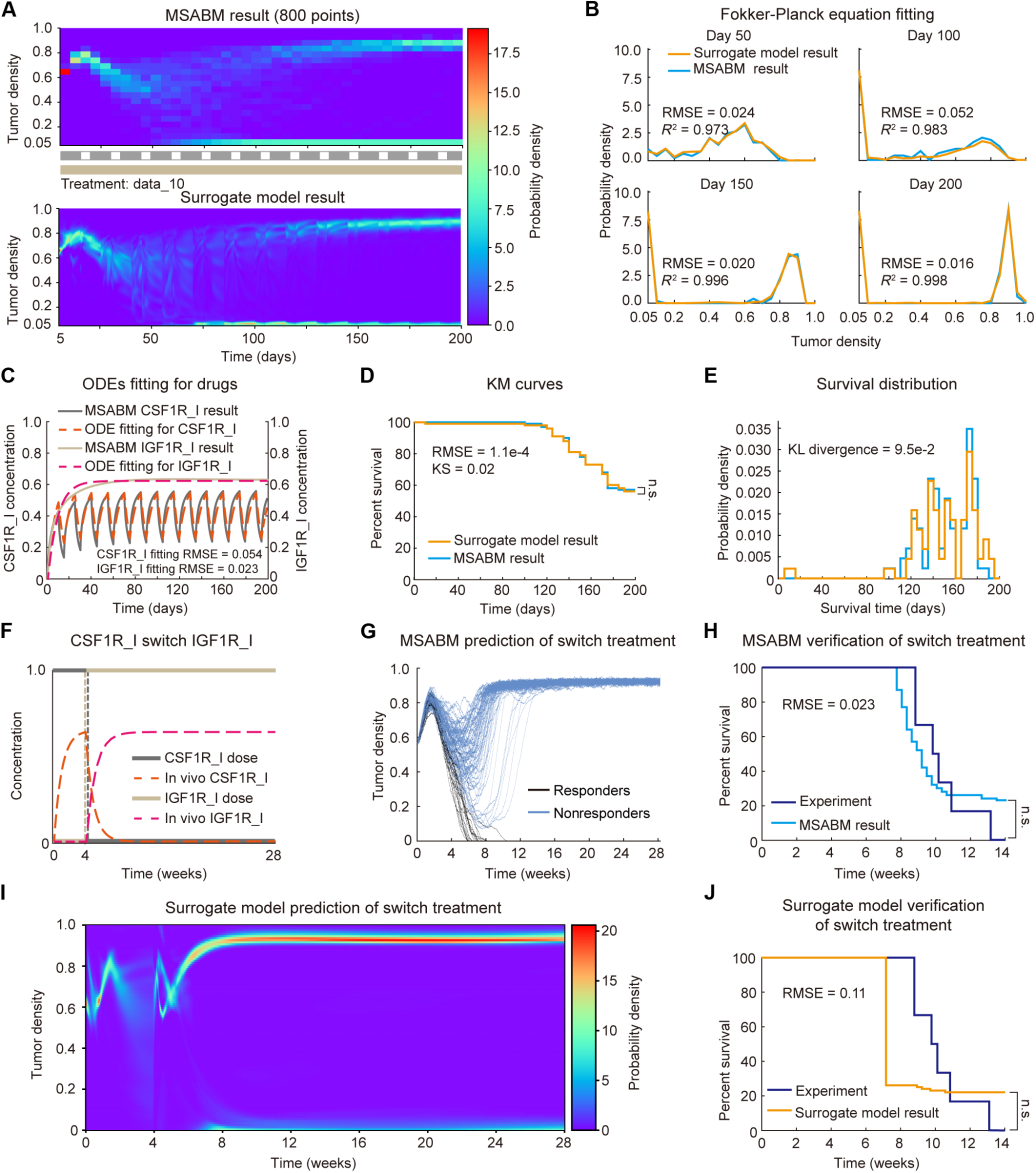
Learning of the surrogate model illustrated with a representative treatment, along with the verification of the surrogate model with switch treatment. (**A**) Comparison of the trained surrogate model with the MSABM simulation under the condition of the representative treatment. The representative treatment here consists of a cyclic CSF1R_I treatment combined with a continuous IGF1R_I treatment, i.e., treatment data_10 as shown in fig. S3. (**B**) Fitting results of Fokker-Planck equation–based surrogate model compared with MSABM simulations at day 50, day 100, day 150, and day 200. The RMSE of each, which is less than 0.06, and the *R*^2^, which is greater than 0.97, indicate high accuracy of the Fokker-Planck equation learning. (**C**) Comparison of the ODEs solutions within the surrogate model with the normalized average concentrations of CSF1R_I and IGF1R_I in MSABM. (**D**) Comparison of survival curves generated by the surrogate model and MSABM multiple simulations (N = 100). Log-rank test, Kolmogorov-Smirnov (KS) statistics, as well as RMSE were used to assess the difference between the two curves. (**E**) Comparison of distributions of survival time derived from the surrogate model and the MSABM. Kullback-Leibler (KL) divergence was used to assess the difference between the two distributions. (**F**) The switch treatment and simulated in vivo drug concentrations. (**G**) Multiple simulations (N = 100) of the MSABM under switch treatment. (**H**) Verification of the survival curve predicted by MSABM under switch treatment against the experimental data (*7*). (**I**) The approximate solution of the Fokker-Planck equation obtained from the surrogate model under switch treatment. (**J**) Verification of the survival curve predicted by the surrogate model under switch treatment through comparison with the experimental data (*7*).

We hypothesize that the Fokker-Planck equation–based surrogate model can recapitulate the temporal evolution of probability distribution of TC number or density, closely matching the MSABM. To validate this hypothesis, we first generated 20 in silico combination treatments of CSF1R_I and IGF1R_I and applied these, along with the initial virtual TME, to the MSABM for multiple simulations (*N* = 100). The processed 20 sets of MSABM outputs were then used as the data-driven constraint of the PINN for learning the surrogate model. A summary comparison between the trained surrogate model and MSABM simulations under all the 20 combination treatments can be found in fig. S3. Here, we present one typical combination treatment in detail for illustration (Fig. 7, A to E). Using the cyclic CSF1R_I (starting with 10 days of continuous treatment followed by 5 days off treatment as one complete cycle) combined with continuous IGF1R_I treatment (i.e., the treatment data_10, as shown in fig. S3) as input, we obtained multiple MSABM simulations of TC density and the output from the trained surrogate model (i.e., evolution of p.d.f. of TC density) (Fig. 7A). We calculate the root mean square error (RMSE) and *R*-squared (*R*^2^) values for the selected four slices at day 50, day 100, day 150, and day 200 for evaluation of the fitting. The RMSE was consistently below 0.06, and the *R*^2^ was consistently above 0.97, indicating that the surrogate model accurately captures the TC density evolution characteristics of the simulated population in the MSABM (Fig. 7B).

In addition, the ODEs used within the surrogate model to describe the average cytokines and drug concentrations also demonstrate a high degree of fit (Fig. 7C). Notably, the Fokker-Planck equations serve as the physical constraint in PINN learning, with some parameters from the ODEs being essential for accurate fitting. Moreover, in the population survival analysis, we obtained survival curves from both the MSABM and the surrogate model simulations. A comparison of the two curves shows a low RMSE of 1.1 × 10^−4^ and Kolmogorov-Smirnov statistic of 0.02, with no statistical significance of the difference as assessed by the log-rank test (Fig. 7D). Last, we calculated the p.d.f. of survival time by taking the negative derivative of each survival curve. The Kullback-Leibler divergence between the two distributions is rather low (0.095), demonstrating a close statistical resemblance (Fig. 7E).

To verify the surrogate model, we applied an additional, independent combination treatment as the input for the surrogate model, perform multiple predictions (*N* = 100) of the MSABM, and then compare the predicted survival results with experimental observations [figure 7G in (*7*)], as shown in Fig. 7 (F to J). The combination treatment is derived from the experiment on established gliomas in mice (*7*). In this experiment, the researchers administered continuous CSF1R_I treatment for 4 weeks, then ceased CSF1R_I, and switched to continuous IGF1R_I treatment (i.e., “switch” treatment). Of note, the drug treatment dosage does not directly correspond to the in vivo drug concentration in the TME. Hence, as explained earlier, we used PDEs in the MSABM and ODEs in the surrogate model to simulate in vivo drug concentrations under treatment (Fig. 7F). Through the MSABM, we found that tumors in approximately 80 individuals experienced shrinkage followed by recurrence (Fig. 7G). This recurrence indicates that replacing CSF1R_I with IGF1R_I is less effective than continuous use of CSF1R_I. Note that in the experimental data, all individuals died, while the MSABM’s predictions showed ~20% survival. We believe that this discrepancy is due to the small sample size (*N* = 6) in the experimental data, leading to potential bias. Nevertheless, considering that the RMSE between the two 14-week survival curves is 0.023, and there is no significance in the log-rank test, the agreement between the prediction and the experiments is acceptable (Fig. 7H). Given the same treatment, we used the surrogate model to obtain an approximate solution of the Fokker-Planck equation describing the TC density within the predicted population (Fig. 7I) and then conducted survival analysis. The RMSE between the predicted and experimental survival curves is 0.11, with no statistical significance of difference assessed by the log-rank test (Fig. 7J).

### Dynamic optimization of combination treatment scheduling

**Fig. 8.**
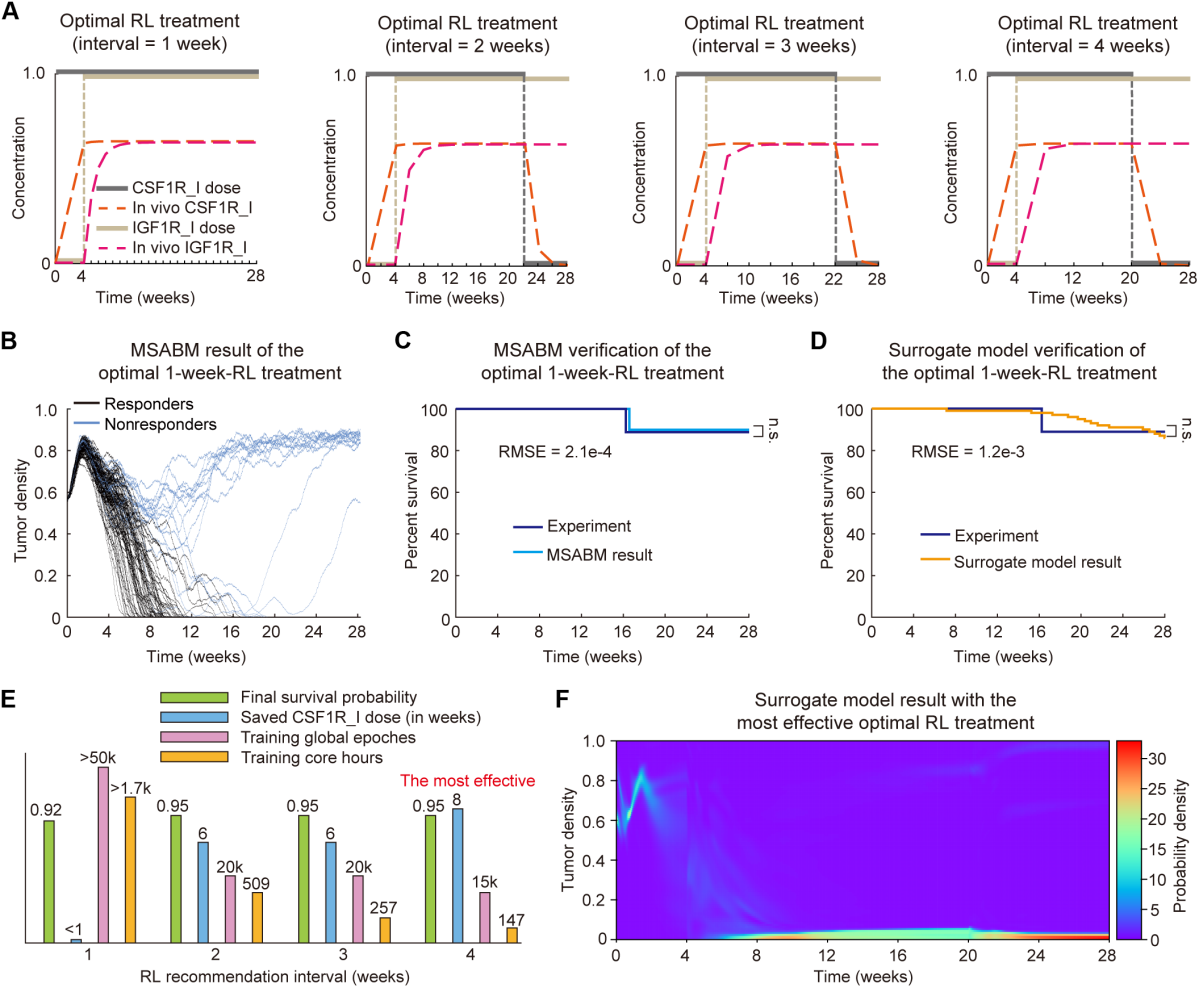
Dynamic optimization of combination treatment scheduling. (**A**) Optimal RL treatment scheduling for 1-week, 2-week, 3-week, and 4-week drug recommendation intervals. (B to D) Verification of the optimal 1-week-RL treatment. (**B**) Multiple predictions (N = 100) for the MSABM under the optimal 1-week-RL treatment. (**C**) Verification of the MSABM under the optimal 1-week-RL treatment against experimental data (*7*). (**D**) Verification of the surrogate model under the optimal 1-week-RL treatment through comparison with the experimental data (*7*). (**E**) Comparison under different RL recommendation intervals. (**F**) The approximate solution to the Fokker-Planck equation derived from the surrogate model under the most effective treatment strategy (i.e., the optimal 4-week-RL treatment).

We next use the M4RL framework to dynamically optimize the scheduling of the combination of CSF1R_I and IGF1R_I, aiming to maximize the population survival rate and reduced drug dosages. To this end, the surrogate model was used to drive the environment in the RL setting, and the A3C method was applied to explore the optimal treatment strategy under different treatment recommendation intervals (Fig. 8). Subsequently, we compared the 1-week-RL combination treatment results of the MSABM and the surrogate model with the experimental results (*7*). Last, an optimum dynamic scheduling of the combination was identified that balances survival rate improvement and drug conservation, as well as training efficiency.

Exploring the drug recommendation interval holds substantial clinical significance. On the basis of the recognition that weekly based treatments have considerable practical applicability in clinical implementation, we explored drug recommendation intervals of 1, 2, 3, and 4 weeks under the constraint of a maximum of 15,000 to 50,000 global training epochs using the improved A3C method (Fig. 8A). In addition, all parameters were controlled to be identical except for the drug recommendation intervals, including the network architectures of both the actor and the critic. The optimal treatment for the 1-week interval is the “add” treatment. We found that this is due to the lower interval generating a longer time series within the fixed total treatment duration (28 weeks), causing that even the 50,000 global training epochs or the network architecture is insufficient to adequately train the critic network to guide the actor network. The 2-week interval is also limited by the longer time series state; however, compared to the 1-week interval, this approach improved population survival rates and conserved medication, resulting in a better overall treatment strategy. We believe that with more time and computational resources, optimal results could eventually be achieved for the above two scenarios as well. Both the 3-week and 4-week intervals resulted in more drug savings; however, the 3-week interval led to the drug savings less pronounced compared to the 4-week interval. In addition, the 4-week interval required fewer computational resources and, by stopping the continuous treatment of CSF1R_I at week 20, saved the most drugs compared to other scenarios.

In addition, we examined the population-level response to the predicted optimal RL treatment with 1-week-interval and compared the predicted survival curve with the experimental data (*7*). The multiple simulations of the MSABM (Fig. 8B) show the stochastic evolution dynamics of TC density in response to the predicted optimal RL treatment with 1-week-interval, demonstrating heterogeneous response of individuals to the treatment. Furthermore, the predicted survival curves derived from both MSABM simulations and the surrogate model exhibit good agreement with the experimental data, as evidenced by statistical test and small RMSE (Fig. 8, C and D). These results, along with the above verification results shown in Fig. 7 (H and J), again verify the accuracy of the MSABM and surrogate model.

Through comparison of the four optimal treatment schedules, we found that the final survival probability of individuals under all four optimal treatments is greater than or equal to 0.92. Nevertheless, the treatment derived from the 4-week interval requires the least computational resources (i.e., training global epochs and core hours with surrogate model simulation time interval = 7 days) while saving the most drugs, indicating a most effective treatment scheduling (Fig. 8E). In addition, the survival curve for the 4-week-RL treatment shows significant difference with that of the continuous combination treatment as assessed by the log-rank test (fig. S4). Accordingly, the Fokker-Planck equation–based surrogate model demonstrates that, under the most effective treatment scheduling, the predicted population’s TC density remains at a low level with a high probability (Fig. 8F).

By the above procedures, we identified the optimal treatment involving continuous use of CSF1R_I ending after week 20, combined with continuous use of IGF1R_I starting from week 5. In addition, we found that increasing the drug recommendation interval from 1 to 4 weeks not only reduces the computational costs for prediction but also facilitates clinical application and drug management. This demonstrates the feasibility and effectiveness of the M4RL framework in design and optimizing combination treatment scheduling for nonresponders to CSF1R_I.

### Testing the optimized treatment scheduling with ST data–based TME

**Fig. 9.**
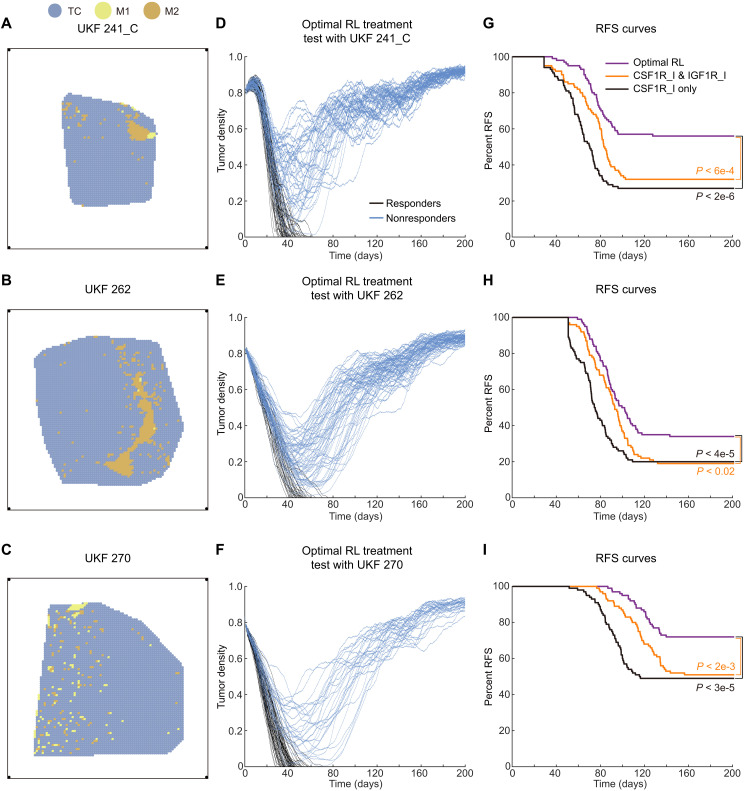
Testing the optimal scheduling of the combination treatment with more realistic TME derived from ST data as MSABM input. (**A** to **C**) The initialization of TME for the MSABM based on the ST data from (*54*, *55*). (**D** to **F**) The MSABM multiple predictions (N = 100) under the predicted optimal RL treatment with (A to C) as the input TME, respectively. (**G** to **I**) Recurrence-free survival (RFS) curves of the optimal RL treatment compared to those of CSF1R_I and IGF1R_I combination treatment and CSF1R_I only treatment, with (A to C) as the input TME for MSABM multiple predictions (N = 100), respectively. The log-rank test *P* value was used to assess the statistical significance of difference between RFS curves.

We further tested the effect of the optimal scheduling of the combination treatment with more realistic TME derived from ST data (*54*, *55*). To estimate spatial distributions of cell densities of TCs, M1s, and M2s in the ST data, we used a ST data deconvolution method, spatialDWLS (*56*), to estimate the proportions of the above three cell types in each spot of the ST data, with a set of single-cell RNA sequencing (scRNA-seq) data of glioma (*57*) (GSE84465) as reference. The estimated cell densities in the three ST datasets were subsequently used to set the virtual TME inputs (Fig. 9, A to C) for the MSABM (as detailed in Materials and Methods). Last, we conducted multiple predictions (*N* = 100) initialized with the ST data–based TMEs under the RL-optimized treatment, and the results were shown in Fig. 9 (D to F). For comparison, we also tested three other treatments, “IGF1R_I only” treatment, “CSF1R_I only” treatment, and “CSF1R_I and IGF1R_I” treatment (fig. S9), using the three ST data–based TMEs as inputs for multiple predictions (fig. S10).

We found that the IGF1R_I only treatment could moderately inhibit TC proliferation but was ineffective in reducing TC density within the TME (fig. S10, A to C). Thus, we chose to compare the recurrence-free survival (RFS) curves obtained from the optimal RL treatment with those from the CSF1R_I only and CSF1R_I and IGF1R_I treatments using the log-rank test. The results verify that the RFS curve of the optimal RL treatment is significantly higher than those of the other two treatments (Fig. 9, G to I), indicating that the optimal RL treatment is substantially superior to both the CSF1R_I only treatment and the CSF1R_I and IGF1R_I treatment.

Meanwhile, we observed that the RFS percent at day 200 under the CSF1R_I and IGF1R_I treatment is comparable to that of the CSF1R_I only case (Fig. 9, G to I), indicating that the combination use of CSF1R_I and IGF1R_I does not confer additional therapeutic benefit over CSF1R inhibition alone in suppressing tumor recurrence. Furthermore, comparing the results of these two treatment strategies, we found that individuals who relapsed under the CSF1R_I only treatment exhibit a tumor density of approximately 0.8 or higher at day 200 (fig. S10, D to F), whereas the CSF1R_I and IGF1R_I treatment is able to control the tumor density of recurrence individuals within the range of about 0.6 to 0.8 (fig. S10, G to I). These results verify that, when CSF1R_I and IGF1R_I are used in combination, the dose scheduling of CSF1R_I has a substantial impact on tumor recurrence rates, while IGF1R_I helps slow tumor proliferation, thereby maintaining a lower tumor density in relapsed individuals compared to the case of CSF1R_I alone.

## DISCUSSION

In this study, we proposed a M4RL framework to bridge the gap between the multiscale mathematical ABM and the RL-based treatment optimization via learning a simplified surrogate model of tumor evolution. We applied this framework to the glioma microenvironment, focusing primarily on the interactions between TCs and TAMs within the TME, as well as the immunotherapy resistance to TAM-targeting CSF1R_I treatment (*44*). First, we established the MSABM to describe the glioma-TME interactions across three scales (i.e., microenvironment, cellular, and molecular scales). The MSABM could faithfully reproduce key experimental observations and is quantitatively verified by the experimental data of population survival rate in preclinical studies (*7*). Then, the spatial, cellular, and parameter analyses of the MSABM shed insights into multilevel mechanisms of immunotherapy resistance of glioma. Subsequently, we learned and validated a Fokker-Planck equation–based surrogate model for the MSABM by using PINN, notably reducing the computational heavy of the MSABM during multiple simulations. Last, we integrated the surrogate model and the A3C algorithm to construct a RL method, dynamically optimizing scheduling of combinations of CSF1R_I and IGF1R_I.

In addition to reproduce well-established knowledge reported by the previous experimental studies [e.g., (*7*, *10*, *58*)], our MSABM also revealed several insights not previously identified in earlier works. For example, in the initial simulation of tumor growth under treatment-naïve condition (movie S2), the number of TCs and various types of TAMs in the TME gradually reaches an equilibrium (Fig. 2A, day 100). Breaking this equilibrium is crucial for effective tumor treatment. It is worth noting that observing this predetectable growth stage is experimentally costly both in vitro and in vivo (*34*). Furthermore, the sensitivity analysis of MSABM reveals that, in addition to the ERK and AKT signaling pathways in TCs and the quiescent state of the TCs, the migration and polarization of TAMs also influence tumor resistance. Moreover, we found in our simulations that cyclic treatment and cut treatment performed worse compared to continuous treatment with CSF1R_I alone. This may be due to that the space generated by M1 phagocytosis is used by ATCs for proliferation as revealed by the nonquiescent model (movies S5 and S6). It also indicates that inhibiting TC proliferation while targeting TAMs in the treatment strategy is essential. However, when we used CSF1R_I for 4 weeks and then switched to IGF1R_I (Fig. 7, F to H), the population survival effect was inferior to that of continuous CSF1R_I treatment. This suggests that the two drugs need to be combined rather than used in succession. Our multiple simulations of MSABM suggest that overlapped joint combinations of CSF1R_I and IGF1R_I can result in a higher population survival rate (fig. S3). These insights gained from the MSABM inform us to design the M4RL framework for dynamic optimization of combination treatment targeting macrophages with CSF1R_I and targeting TCs with IGF1R_I.

To identify the optimal scheduling of combination treatment strategies, RL must explore a vast array of action combinations within the action space. This requirement is also the direct reason why the MSABM cannot directly inform the updates of the RL environment. Although we used a surrogate model, asynchronous updates are still necessary to enhance the efficiency of the dynamic optimization process. We chose to collect the state and action in the environment on a weekly interval basis to avoid excessively long pseudo-time series that would hinder the efficient training of the critic network, which guides the actor in selecting actions. Moreover, simplified network architectures (as shown in table S5) fail to effectively learn the complex temporal dependencies and transitions in long pseudo-time series (Fig. 8A). When we upgraded the critic network to a more complex architecture (as shown in table S5) under the 1-week drug recommendation interval setting, we were able to learn a treatment policy equivalent to the optimal 4-week-RL treatment after 50,000 global training epochs (fig. S11). However, the complex critic network required 1960 core hours for 50,000 global training epochs, compared to only 1700 core hours (Fig. 8E) for the simpler architecture. Although computational efficiency is not a major concern when identifying optimal treatment strategies for general patients in this study, we included the computational resource analysis (Fig. 8E) to illustrate the complete workflow of the M4RL framework. Such considerations become particularly important in scenarios requiring real-time personalized clinical support, such as generating multidrug treatment strategies tailored to individual patients.

In our M4RL framework, learning a surrogate model to predict the survival outcomes of populations under different treatments is a critical step. This approach avoids the substantial CPU resources and time required for multiprocessing in the parallel MSABM. We input 20 treatment regimens into the MSABM and used the resulting simulated data to train the PINN for approximating the solution to the Fokker-Planck equations (fig. S3). In the MSABM’s multiple predictions, the initial virtual TME inputs are designed to ensure that the number of TCs in the TME across various simulations is roughly consistent, yet their spatial distributions vary. This approach, we believe, more accurately simulates the spatial heterogeneity observed among different individuals. The surrogate model yielded consistent predictions with both the MSABM and the experimental data (Figs. 7H and 8D); however, its performance was less accurate near the discontinuities of the dose function, particularly at the fourth week (Figs. 7I and 8F). This may be due to insufficiency of the sampled data during the training phase of the PINN. Meanwhile, it highlights the need to enhance the constraint role of the Fokker-Planck equations in the PINN, especially around these discontinuity points.

Our MSABM has several limitations, which could be improved in the future studies. For instance, we did not consider the impact of other signaling pathways on TC activities (*7*), nor did we explore the feasibility of combination treatments with other inhibitors such as EGFR inhibitor (*43*, *51*, *59*). Furthermore, we only accounted for macrophages appearing as M0, M1, and M2 types, without considering their heterogeneous phenotypes in a continuous spectrum (*6*, *60*). Also, although we included vascular cells in the TME, they were only used for recruiting macrophages and representing drug penetration; we did not investigate the impact of blood vessels on the TME evolution and drug resistance (*35*, *39*). In addition, previous experimental studies have shown that immune cells and other microenvironment variables may influence the treatment efficacy of gliomas (*61*, *62*). Therefore, incorporating other immune cells associated with gliomas, such as T cells (*63*), into the multiscale model will enhance the simulation of a more realistic TME and may facilitate the design of immunotherapeutic strategies, potentially in combination, for improving glioblastoma treatment.

In the current MSABM, we focus on a simplified molecular-scale signaling network of TCs, derived from prior experimental studies. To expand this network into a more comprehensive framework, we applied our recently developed computational tool, stMLnet (*64*), which integrates ST data with comprehensive signaling interaction databases to construct multilayer cell-cell communication networks. Using one of the three ST datasets (sample UKF 262), we specifically analyzed macrophage-to-glioma cell signaling interactions. This investigation identified key pathways central to our MSABM, including the EGF-EGFR and IGF1-IGF1R pathways, along with their downstream transcription factors and target genes (fig. S12). In addition, we detected multiple other prominently activated signaling cascades. Current efforts are directed toward exploring the integration of these ST data–driven multilayer signaling networks into agent-based modeling of intercellular interactions.

Our M4RL framework also holds substantial potential for extension to other tumor types and the combination uses of more than two drugs and dynamic calibration of the model using intermediate data to optimize personalized treatment strategies. However, these extensions also present several challenges. The feasibility of constructing models for various tumor types and implementing multidrug treatment strategies relies on supporting evidence from organoid models or animal experiments. In addition, obtaining intermediate data for real-time calibration of personalized models, as well as acquiring high-throughput ST or scRNA-seq data, also poses practical challenges in terms of data accessibility, cost, and standardization. Moreover, the high dimensionality of the RL action space in optimizing multidrug treatment strategies and the time-sensitive nature of personalized clinical decision-making necessitate minimizing the computational cost of running our M4RL framework as much as possible.

Overall, in this study, we formulated a M4RL framework to characterize the evolutionary dynamics of glioblastoma and to optimize combination treatment scheduling. We explored the multiscale characteristics of glioma evolution under different drug treatment regimens, revealed the pivotal roles of the TME in glioma development and resistance processes, and efficiently optimized the treatment scheduling by combining CSF1R_I with IGF1R_I. The optimal combination schedule we identified substantially improved population survival rates while minimizing drug dosage usage. Our approach holds immense potential for simulating, predicting, and optimizing treatment protocols in various types and levels of tumor evolutions.

## MATERIALS AND METHODS

The M4RL framework contains three key components, including multiscale agent-based modeling, surrogate model learning informed by Fokker-Planck equations, and RL-based dynamic optimization. The M4RL does not only learn governing equations of tumor growth dynamics but also provides a feasible way to greatly enhance computational efficiency of RL for dynamic optimization in such complex systems often simulated by agent-based models.

Below, we describe the details of the M4RL framework by taking glioma-macrophage interactions in the context of immunotherapy resistance of glioblastoma as a critical biological scenario for illustration. We first develop a MSABM that includes three scales: the diffusion of cytokines and drug molecules in the TME (microenvironment scale), interactions between TCs and TAMs as well as their phenotypic switches (cellular scale), and the inter/intracellular signaling pathways (molecular scale). Subsequently, we simplify the MSABM into a Fokker-Planck equation–based surrogate model to describe the evolution of TC population, which is learned by using PINNs for approximate representations. Ultimately, we use the simplified surrogate model to guide the RL environment in the pursuit of optimal treatment strategies.

### Multiscale agent-based modeling

Our MSABM uses a 100 by 100 lattice-based 2D grid to model the glioma microenvironment, corresponding to a tissue section of 1.5 mm by 1.5 mm. As shown in Fig. 1, TCs can exist in two states: active and quiescent. In addition, our model includes three types of TAMs (i.e., M0, M1, and M2), along with DCs and vascular cells. Similar to many other on-lattice models, we further assume that only one cell can occupy a grid cell at any given time (*34*, *41*). We use PDEs to simulate the diffusion of cytokines and drug molecules within the TME, numerically solved using the finite difference method on the lattice. In terms of cellular activities, TAMs and TCs can engage in chemotactic migration, while DCs and vascular cells remain stationary. In addition, M1 macrophages can phagocytose TCs (*2*, *6*, *49*) and DCs (*65*) and then move to the phagocytosed cells’ position. Moreover, ODEs are used to describe the intracellular signaling pathways of TCs. The schematic of MSABM is illustrated in Fig. 1. Detailed mathematical formulae of the MSABM are described in text S1, along with the rules for agents and parameters in MSABM are summarized in tables S1 and S2, respectively.

### Simulation and analysis of the multiscale model

#### Single simulation and multiple predictions

We assume that initially there are four stem TCs located at the center of the lattice, while vascular cells are randomly distributed near the boundary, with higher density at the edges and lower density toward the center. M0 macrophages are recruited from the vasculature into the TME at a calculated rate and undergo differentiation until the maximum macrophage carrying capacity is reached. This phase of the simulation lasts approximately 20 days. As the simulation continues for another 20 days, we observe that the CSF1 secreted by TCs cause most TAMs to polarize into the M2 form, thereby protecting the TCs and allowing them to continue proliferating. After about 40 days in the simulation, we obtain a virtual TME with a developed tumor, as shown in movie S1, and we set it as the initial virtual TME for both the subsequent treatment-naive simulation and the continuous CSF1R_I treatment simulation.

Our model can perform both single and multiple simulations by adjusting the size of *N*. In a single simulation (i.e., *N* = 1), all model parameters are fixed at their baseline values; however, this approach does not eliminate the influence of the model’s inherent randomness. Initially, we place four tumor stem cells at the center of the lattice. These stem cells have a longer life span and a shorter cell cycle compared to regular TCs. As the stem cells divide, the life span of the newly generated cells is halved, and their cell cycle is doubled, until they reach the life span and cell cycle duration typical of regular TCs. We simulated the process of tumor growth reaching a standardized density of 0.58 through a single simulation and saved the TME of the formed tumor for subsequent work. The workflow of the single simulation is shown in fig. S5, while the workflow of simulating behaviors of TCs and TAMs within the TME are depicted in figs. S6 and S7, respectively.

Multiple simulations (*N* = 100) are used for conducting population survival analysis. We sample certain parameters in the parameter space to simulate intertumor heterogeneity within the population of *N* individuals. In this context, we selected $\alpha_{\text{ERK}}$, which is related to tumor proliferation, $\alpha_{C}$, which is associated with macrophage polarization, and $\alpha_{I}$, which is related to CSF1R_I responsiveness. We generated three normal distributions for these three parameters, with the basal value as the expected value and 0.1 times the basal value as the variance, and randomly sampled *N* groups from these distributions. The detailed workflow is illustrated in fig. S8.

#### Survival analysis

The survival curve is a nonparametric statistic to estimate the survival function *S*(*t*) from lifetime data, and it provides a way to visualize the probability of survival at different time points, which is expressed as a proportion of the total population at risk (*66*). The estimator of survival function *S*(*t*) is calculated as the following formula with product operator

$$\hat{S}\left(t\right)=\prod_{i:t_{i}\leq t}\left(1-\frac{d_{i}}{n_{i}}\right)$$ (1)

where *d_i_*(*t*) and *n_i_*(*t*) are the occurrence number of events (e.g., death or recurrence) at time *t_i_* and the risky individual number just before time *t_i_*, respectively. Each term in Eq. 1 represents the probability of survival at a specific time point. By multiplying them together, one can obtain the cumulative probability of survival up to time *t*.

Here, to maintain consistency with the presentation of experimental results (*7*), we use “percent survival” to describe the survival characteristic of the predicted population. The function of percent survival is defined as

$$S_{p}\left(t\right)=\left(1-\frac{d\left(t\right)}{N}\right)\times 100$$ (2)

where *N* represents the number of individuals in the predicted population (e.g., the number of the multiple predictions) and $d\left(t\right)=\sum_{i=1}^{N}1_{\left\{T_{d}^{i}\leq t\right\}}$ is the number of dead individuals at time *t*. Here, $T_{d}≔\text{inf}\left\{t\;\left|\;\frac{N_{\text{TC}}\left(t\right)}{N_{\text{TC}}^{\text{max}}}\right.\geq \text{Thr}\right\}$ is defined as the survival time of each individual, with *N*_TC_(*t*) representing the number of TCs within the TME at time and the maximum carrying capacity of TCs within the TME. Thr = 0.896 is a predefined threshold determining individual’s death or recurrence. $1_{\left\{T_{d}^{i}\leq t\right\}}$ is an indicator function that equals 1 if $T_{d}^{i}\leq t$, and 0 otherwise. In addition, the simulation (or prediction) time in the MSABM is constrained to the interval 0<t≤T, where *T* represents the maximum simulation (or prediction) duration. Notably, we set *T* = 100 days under the treatment-naïve condition and *T* = 200 days under the continuous CSF1R_I treatment condition.

We subsequently use the log-rank Mantel-Cox test to compare the percent survival curves of predictions and experimental observations (*67*). To put it simply, we use the log-rank test here to compare the predicted number of events with the observed counts in experiment, aiming to determine whether there are statistically significant differences between the two. The test statistic $Z=\frac{O-E}{\sqrt{V}}$ is a standardized statistic, where *O* and *E* denote the observed experimental and expected predicted number of death up to time *T*, respectively. $V=\sum_{j}\frac{d_{j}^{2}}{n_{j}}\left(\frac{N-n_{j}}{N-1}\right)$ is the variance of the observed number of death in experiment. Then, the *P* value of log-rank test is calculated as

$$P_{\text{value}}=2\times \left[1-\Phi \left(Z\right)\right]$$ (3)

where Φ is the cumulative probability function of the standard normal distribution. A small *P* value (commonly <0.05) suggests that the differences between survival curves of the prediction and experiment groups is statistically significant.

#### Fokker-Planck equation–based surrogate model

To simplify the MSABM for rapid prediction, we develop a hybrid surrogate model composed of SDEs and ODEs to describe the evolution in cell densities and cytokine/drug concentrations in the TME. Transforming the SDEs into Fokker-Planck equations allows us to describe the evolution of the probability distribution of cell density at the population level. The details of the derivation of the hybrid surrogate model and the generation of data for training PINN can be found in text S2.

The choice of treatment regimen directly affects the progression of cancer in patients. The differences between various treatment regimens primarily manifest in the selection of drug types and the dosing variations over time. In this context, we refer to the combination of multiple drugs as “combination treatment.” According to the variation of drug dosage over time, treatment regimens are typically categorized as continuous treatment, cyclic treatment, adaptive treatment, and cut treatment (fixed-duration treatment). Further details about the simulated treatments for generating the training data are described in text S2.1.

The development of the SDE-ODE hybrid surrogate model is described in text S2.2. In particular, the SDE for describing the stochastic changes in TC density is given as follows

$$\frac{{\mathrm{dC}}_{\text{TC}}^{*}}{\mathrm{dt}}=k_{1}^{*}{\tilde{r}}_{\text{TC}}^{*}C_{\text{TC}}^{*}-k_{2}^{*}{\tilde{d}}_{\text{TC}}^{*}C_{\text{TC}}^{*}$$ (4)

where the variables and parameters marked with a star symbol * are related to surrogate modeling. Specifically, ${\tilde{d}}_{\text{TC}}^{*}=d_{\text{TC}}$$\left\{1,+,k_{8}^{*}d_{\text{TM}1}^{*}\left[1+\sigma_{\text{TC}}^{*}\varepsilon \left(t\right)\right]C_{M1}^{*}\right\}$ represents the death rate of $C_{\text{TC}}^{*}$. $k_{i}^{*},i\in {\mathbb{N}}^{+}$ denotes the adjustment parameters required for simplifying PDEs to SDEs. ε(t) is the Gaussian white noise and ε(t)dt=dW(t), with W(t) being Brownian motion. Substituting ${\tilde{d}}_{\text{TC}}^{*}$ into Eq. 4 and transforming it into Fokker-Planck form (*68*), we get

$$\begin{array}{c} \frac{\partial P_{\text{TC}}}{\partial T}=-\frac{\partial }{\partial C_{\text{TC}}^{*}}\left[\mu_{\text{TC}}\left(t,C_{\text{TC}}^{*}\right)P_{\text{TC}}\left(t,C_{\text{TC}}^{*}\right)\right]\\ +\frac{1}{2}\frac{\partial^{2}}{\partial {{\text{C}}_{\text{TC}}^{\text{∗}}}^{2}}\left[\nu_{\text{TC}}\left(t,C_{\text{TC}}^{*}\right)P_{\text{TC}}\left(t,C_{\text{TC}}^{*}\right)\right] \end{array}$$ (5)

where $P_{\text{TC}}\left(t,C_{\text{TC}}^{*}\right)$ is the p.d.f. of the random variable $C_{\text{TC}}^{*}$ at time *t*. $\mu_{\text{TC}}\left(t,C_{\text{TC}}^{*}\right)=k_{1}^{*}{\tilde{r}}_{\text{TC}}^{*}C_{\text{TC}}^{*}\left(1-C_{\text{TC}}^{*}\right)-k_{2}^{*}d_{\text{TC}}\left(1+k_{8}^{*}d_{\text{TC}}^{M1}C_{M1}^{*}\right)C_{\text{TC}}^{*}$ is the drift coefficient, while $\nu_{\text{TC}}\left(t,C_{\text{TC}}^{*}\right)=-k_{2}^{*}d_{\text{TC}}k_{8}^{*}d_{\text{TC}}^{M1}C_{M1}^{*}C_{\text{TC}}^{*}\sigma_{\text{TC}}$ is the diffusion coefficient. More details are provided in text S2.3.

By embedding the physical constraint into the loss function of the neural network, PINNs can effectively learn from existing data and predict the behavior of the system (*69*, *70*). See introduction of the PINN in text S2.5. We selected 20 sets of combination treatments of CSF1R_I and IGF1R_I with various regimens and input each of these treatment plans into the MSABM for multiple predictions (*N* = 100). The output data were processed to obtain the time-series probability distributions of TC density within the simulated population, which serves as data-driven constraint for the PINN. The Fokker-Planck equations (i.e., Eq. 5), along with other ODEs in the hybrid surrogate model, serve as a physical constraint for the PINN. In general, we can define the PINN as $\begin{array}{c} f_{\text{TC}}^{P}=\frac{\partial u_{\text{TC}}^{P}}{\partial t}+\frac{\partial }{\partial C_{\text{TC}}^{*}}\left(\mu_{\text{TC}}\cdot u_{\text{TC}}^{P}\right)-\frac{1}{2}\frac{\partial^{2}}{\partial {{\text{C}}_{\text{TC}}^{\text{∗}}}^{2}}\left(\nu_{\text{TC}}\cdot u_{\text{TC}}^{P}\right) \end{array}$. Here, $u_{\text{TC}}^{P}\left(t,C_{\text{TC}}^{*};\theta_{\text{TC}}^{P}\right)$ represents the deep neural network (DNN) approximation of the latent solution in Eq. 5, where $\theta_{\text{TC}}^{P}$ are the trainable parameters in DNN.

Therefore, the loss function of the PINN is defined as

LTCP(θTCP,σ∗;PDuTC,PDcTC)=LdTCP(θTCP;PDuTC)+LdTCP(θTCP,σ∗;PDcTC) (6)

where LdTCP(θTCP;PDuTC)=1NuTCP∑i=1NuTCP[uTCP(ti,ci;θTCP)−PTC(ti,ci)]2 corresponds to the data-driven constraint and LpTCP(θTCP,σ∗;PDcTC)=1NcTCP∑i=1NcTCP[fTCP(ti,ci;θTCP,σ∗)]2 the physical constraint. $P_{\text{TC}}\left(t_{i},c_{i}\right)$ represents the probability density of TCs with cell number (normalized cell density) $c_{i}$ at time $t_{i}\in \left[0,T\right]$. DuTCP=(ti,ci)∣i=1,…,NuTCP and DcTCP=(ti,ci)∣i=1,…,NcTCP represent the prior point set and randomly sampled point set in the domain [0,T]×[0,1], respectively. More explanations of the above loss function and details of the training steps for the surrogate model are provided in text S2.6. The parameters in the PINN training are listed in table S3. The comparison between the trained surrogate model and the MSABM simulations under the 20 sets of treatments with CSF1R_I and/or IGF1R_I are given in fig. S3.

#### Surrogate model–based RL

In a standard RL setup, an agent is the learner making decisions within the environment, which is typically the external system interacting with the agent. An agent interacts with the environment over multiple discrete time steps. At each time step, the state is used to describe the current situation of the environment, commonly denoted as $s_{t}\in S$. In addition, $a_{t}\in A$ is the action taken by the agent at a specific time; the reward is the immediate feedback signal received by the agent from the environment, represented as $r_{t}\left(s_{t},a_{t}\right)$. The cumulative reward, denoted as $R_{t}=\sum_{k=0}^{\infty }\gamma^{k}r_{t+k}$, is the total reward obtained from the start of the interaction until its end, where the discount factor γ represents the weight of future reward. A higher value of γ indicates a strong emphasis on future rewards. Thus, RL is a machine learning approach in which an agent learns how to take actions by interacting with the environment to maximize the cumulative reward $R_{t}$ at each step (*71*). More details about the setups in a standard RL can be found in table S4.

In our M4RL approach, we use the surrogate model to guide the evolution of the environment and use the A3C method (*53*) to explore the optimal CSF1R_I and IGF1R_I combination treatments (Fig. 6B). This method consists of a global network, which can be asynchronously updated through multiple processes during training and helps avoid the high computational costs associated with graphics processing unit (GPU)–based mining algorithms (*72*). We also incorporate the entropy of the policy into the objective function to enhance exploration by preventing premature convergence to suboptimal strategies (*73*). Specifically, we fix a 4-week continuous CSF1R_I treatment before drug recommendation, in accordance with the setup of first 4 weeks of the experiment (*7*). In addition, as observed in the parameter sensitivity analysis described above (Fig. 4, E to G), differentiation between responders and nonresponders to CSF1R_I typically emerges 30 to 40 days after the initiation of continuous CSF1R_I treatment, it is reasonably assumed that this setup will not negatively affect tumor treatment. More details about the surrogate model–based RL approach and the A3C method–based training procedures are provided in text S3, while RL method pseudocode is shown in algorithm S1 and the relevant parameters of RL method can be found in table S5. Meanwhile, the calculation of the reward function of RL is shown in algorithm S2, and its relevant parameters are listed in table S6.

#### MSABM simulations with ST data–based TME

We used ST data–based TMEs as inputs for the MSABM and conducted multiple predictions under four treatment strategies (fig. S9). The results of multiple predictions were then subjected to RFS analysis.

To incorporate the ST data of glioblastoma (*54*, *55*) into the MSABM, we rescaled the initialization of the MSABM. Specifically, we adjusted the grid size of our MSABM to match the ST spot size. In this setup, the MSABM still uses a 100 by 100 on-lattice grid, where each lattice grid represents a spot with a diameter of 60 μm. Accordingly, we rescaled the lattice diameter–related parameters in the MSABM. Then, we applied a ST data deconvolution tool, spatialDWLS (*56*), to estimate the cell proportions within each spot of the ST data, using scRNA-seq data (GSE84465) from (*57*) as the reference. Subsequently, we interpolated the cell proportions of TCs, M1, and M2 macrophages in the ST data based on their spot locations onto the 100 by 100 on-lattice grid. Last, we annotated the cell type of each spot according to the maximal cell type proportion by assuming that each lattice grid represents a group of the same type of cells.

We tested the effects of the four treatments, including IGF1R_I only treatment, CSF1R_I only treatment, CSF1R_I and IGF1R_I treatment, and optimal RL treatment (fig. S9). In accordance with the experimental drug administration settings (*7*), the IGF1R_I only treatment (fig. S9A) involves no drug administration for the first 4 weeks, followed by continuous administration of IGF1R_I at a concentration of 1.0 (normalized, similarly applied below). For the CSF1R_I only treatment (fig. S9B), CSF1R_I is administered continuously at a concentration of 0.7, ensuring that the total drug dosage of CSF1R_I matches that of the optimal RL treatment. The CSF1R_I and IGF1R_I treatment (fig. S9C) is a combination of the IGF1R_I only and CSF1R_I only treatments, while the optimal RL treatment (fig. S9D) refers to the 4-week-RL combination treatment scheduling obtained from Fig. 8.

## Supplementary Materials

### The PDF file includes:

Supplementary Text S1 to S3 Algorithm S1 and S2 Figs. S1 to S12 Tables S1 to S6 Legends for movies S1 to S6 References
