# Supplementary material for: Multiscale mathematical model-informed reinforcement learning optimizes combination treatment scheduling in glioblastoma evolution
Source: Sci Adv. 2025 Aug 8;11(32):eadv3316. doi: 10.1126/sciadv.adv3316 (PMC13155510; doi:10.1126/sciadv.adv3316)
Supplement: Supplementary file 1 — Supplementary Text S1 to S3 Algorithm S1 and S2 Figs. S1 to S12 Tables S1 to S6 Legends for movies S1 to S6 References [file sciadv.adv3316_sm.pdf]

Science Advances
AAAS

Supplementary Materials for

Multiscale mathematical model-informed reinforcement learning optimizes combination treatment scheduling in glioblastoma evolution

Zeming Liu *et al.*

Corresponding author: Xiaoqiang Sun, sunxq6@mail.sysu.edu.cn

Sci. Adv. **11**, eadv3316 (2025)
DOI: 10.1126/sciadv.adv3316

The PDF file includes:

Supplementary Text S1 to S3
Algorithm S1 and S2
Figs. S1 to S12
Tables S1 to S6
Legends for movies S1 to S6
References

Other Supplementary Material for this manuscript includes the following:

Movies S1 to S6

Supplementary Text
S1. Multiscale mathematical modeling

Multiscale mathematical modeling is the first main component of our M4RL framework. Our MSABM utilizes a 100×100 on-lattice 2D grid to simulate the glioma microenvironment, representing a tissue slice of 1.5 mm × 1.5 mm. As shown in Fig. 1, tumor cells can exist in two states: active and quiescent. Additionally, our model includes three types of TAMs (i.e., M0, M1, and M2), along with dead cells and vascular cells. Similar to many other on-lattice models, we assume that only one cell can occupy a grid cell of the lattice at any given time (*34, 41*). PDEs were employed to simulate the diffusion of cytokines and drug factors within the TME. ODEs are used to describe the intracellular signaling pathways of tumor cells. In terms of cellular activities, TAMs and tumor cells can engage in chemotactic migration, while dead cells and vascular cells remain stationary. Additionally, M1 macrophages can phagocytose tumor cells (*2, 6, 49*) and dead cells, then move to their space. The rules of agents in the MSABM are summarized in table S1, while the parameters used in the MSABM are listed in table S2.

S1.1. Diffusions in the TME

In our MSABM, there are three types of cytokines and two drugs diffusing within the TME. Among these, M2 macrophages release pro-carcinogenic factors EGF and IGF1 (*48, 49*), while tumor cells secrete CSF1 (*7*). EGF and IGF1 diffuse within the TME and bind to their respective receptors on tumor cells, regulating TCs proliferation through the signaling pathways that will be described below. Additionally, tumor cells exhibit chemotactic migration toward EGF and IGF1 (*8, 9*). CSF1, on the other hand, binds to CSF1R on TAMs, promoting their polarization to the M2 phenotype (*10, 59*). Moreover, TAMs migrate toward regions of high CSF1 concentration due to their chemotactic response to CSF1.

The PDEs describing the diffusion of cytokines is formulated as the following equation (*44*):

$$\frac{\partial C_W}{\partial t} = D_W \Delta C_W + \sum_m S_W^m C_m \left(1 - \frac{C_W}{C_W^{\max}}\right) - d_W C_W. \tag{1}$$

In the above equation, $C_k(t,\mathbf{x})$ is a function of time t and position $\mathbf{x}$, representing the spatiotemporal concentration of cytokine W in the TME. D_W is the diffusion coefficient of cytokine W, S_W^m represents the secretion rate of cytokine W from cell type m, and $C_m(t,\mathbf{x})$ denotes the spatiotemporal density of cell type m. $C_W^{\max}$ represents the upper limit of the concentration of cytokines in the TME. Additionally, d_W represents the degradation rate of cytokine W. We note that in our model, we assume that the secretion rates of CSF1 and EGF remain constant while S_{IGF1}^{M2} increases with the continued use of the drug CSF1R_I (*7*) :

$$S_{IGF1}^{M2} = S_{IGF1}\left(A_0 + S_A \int_0^t [CSF1R\_I](s)ds\right). \tag{2}$$

where S_{IGF1} represents the basal secretion rate of IGF1, A_0 denotes basal secretion coefficient, while S_A represents the cumulative effect coefficient of CSF1R_I on the secretion of IGF1, and $[CSF1R\_I](t,\mathbf{x})$ represents the spatiotemporal concentration of drug CSF1R_I (*44*).

The evolution of concentrations of drug factors depends on vascular permeability, as described as follows (*43*):

$$\frac{\partial[drug]}{\partial t} = D_{drug}\Delta[drug] + \chi_{VC}q_{drug}\left(dose_{drug} - [drug]\right) - \sum_m C_m u^m_{drug}[drug] - \eta_{drug}[drug]. \tag{3}$$

In equation (3), $[drug](t,\mathbf{x})$ represents the spatiotemporal concentrations of drug factors in the TME, while the spatial distribution of vascular cells $\chi_{VC}(\mathbf{x})$ is represented as fixed due to their stationary nature. $C_m(t,\mathbf{x})$ denotes the spatiotemporal density of cell type m. In addition, D_{drug}, q_{drug}, $dose_{drug}$, u^m_{drug} and η_{drug} represent the diffusion coefficient, vascular permeability, the in-blood concentration, cell type m uptake rate and natural decay rate of drug, respectively. Notably, we employed zero-flux boundary conditions and used the finite difference method for discretization, along with the forward Euler method for numerically solving the PDEs (Fig. 1G).

S1.2. Cell migration

Generally, the PDE describing the spatiotemporal evolution of tumor cell density $C_{TC}(t,\mathbf{x})$ is given by (*44*):

$$\frac{\partial C_{TC}}{\partial t} = D_{TC}\Delta C_{TC} - \alpha_{TEGF}\nabla\left(C_{TC}\nabla[EGF]\right) - \alpha_{TIGF1}\nabla\left(C_{TC}\nabla[IGF1]\right) + \tilde{r}_{TC}C_{TC} - \tilde{d}_{TC}C_{TC}, \tag{4}$$

Where D_{TC} denotes the diffusion coefficient of the tumor cells, α_{TEGF}, α_{TIGF1} represents the chemotactic coefficients of tumor cells to EGF and IGF1, respectively. $[EGF]$ and $[IGF1]$ denote the cytokine concentrations as described in the main text and the following text. $\tilde{r}_{TC}$ and $\tilde{d}_{TC}$ represents the proliferation rate and death rate of the tumor cells. For convenience, we use α_{TE}, $[E]$, α_{TI} and $[I]$ to replace α_{TEGF}, $[EGF]$, α_{TIGF1} and $[IGF1]$ in the following descriptions of tumor cell migration. Sine the migration of tumor cells in the TME is directly related to the diffusion term and the chemotaxis term, we rely on the following equation to formulate the migration probability of tumor cell migration.

$$\frac{\partial C_{TC}}{\partial t} = D_{TC}\Delta C_{TC} - \alpha_{TE}\nabla\left(C_{TC}\nabla[E]\right) - \alpha_{TI}\nabla\left(C_{TC}\nabla[I]\right). \tag{5}$$

Let (ih, jh) represent the index of the position $\mathbf{x}$ in the lattice, and discretizing eq. (5) with the Euler central difference method (*74*), we obtain

$$^{q+1}C_{TC}^{(i,j)} = P_{TC}^{0}\, {}^{q}C_{TC}^{(i,j)} + P_{TC}^{1}\, {}^{q}C_{TC}^{(i+1,j)} + P_{TC}^{2}\, {}^{q}C_{TC}^{(i-1,j)} + P_{TC}^{3}\, {}^{q}C_{TC}^{(i,j+1)} + P_{TC}^{4}\, {}^{q}C_{TC}^{(i,j-1)}, \tag{6}$$

where coefficients P_{TC}^0, P_{TC}^1, P_{TC}^2, P_{TC}^3 and P_{TC}^4 are directly proportional to the probabilities of the tumor cells being stationary or moving left, right, down, or up at time $t = q \cdot k$ (*75*). k and h are the time step size and spatial step size.

$$\begin{aligned}
P_{TC}^0 &= 1 - \frac{4kD_T}{h^2} - \frac{k\alpha_{TE}}{h^2}\left([E]_{(i+1,j)}^q + [E]_{(i-1,j)}^q + [E]_{(i,j+1)}^q + [E]_{(i,j-1)}^q - 4[E]_{(i,j)}^q\right) \\
&\quad - \frac{k\alpha_{TI}}{h^2}\left([I]_{(i+1,j)}^q + [I]_{(i-1,j)}^q + [I]_{(i,j+1)}^q + [I]_{(i,j-1)}^q - 4[I]_{(i,j)}^q\right), \\
P_{TC}^1 &= \frac{kD_T}{h^2} - \frac{k\alpha_{TE}}{4h^2}\left([E]_{(i+1,j)}^q - [E]_{(i-1,j)}^q\right) - \frac{k\alpha_{TI}}{4h^2}\left([I]_{(i+1,j)}^q - [I]_{(i-1,j)}^q\right), \\
P_{TC}^2 &= \frac{kD_T}{h^2} - \frac{k\alpha_{TE}}{4h^2}\left([E]_{(i-1,j)}^q - [E]_{(i+1,j)}^q\right) - \frac{k\alpha_{TI}}{4h^2}\left([I]_{(i-1,j)}^q - [I]_{(i+1,j)}^q\right), \\
P_{TC}^3 &= \frac{kD_T}{h^2} - \frac{k\alpha_{TE}}{4h^2}\left([E]_{(i,j+1)}^q - [E]_{(i,j-1)}^q\right) - \frac{k\alpha_{TI}}{4h^2}\left([I]_{(i,j+1)}^q - [I]_{(i,j-1)}^q\right), \\
P_{TC}^4 &= \frac{kD_T}{h^2} - \frac{k\alpha_{TE}}{4h^2}\left([E]_{(i,j-1)}^q - [E]_{(i,j+1)}^q\right) - \frac{k\alpha_{TI}}{4h^2}\left([I]_{(i,j-1)}^q - [I]_{(i,j+1)}^q\right).
\end{aligned} \tag{7}$$

Notably, we need to set $h = 15\ \mu m$ as the grid spacing and then choose an appropriate value for k to ensure that the five values mentioned above are all greater than 0. Only after normalization can these five values be considered as probabilities.

Similarly, we denote $C_M(t, \mathbf{x})$ as the spatial distribution of macrophage density (including M0, M1, and M2) over time. The migration of TAMs is related to their diffusion and chemotactic response to CSF1 (*7, 44*):

$$\frac{\partial C_M}{\partial t} = D_M \Delta C_M - \alpha_{MC} \nabla\left(C_M \nabla [C]\right), \tag{8}$$

where D_M is the diffusion coefficient of the TAMs, α_{MC} and $[C]$ are represent the chemotactic coefficient of TAMs to CSF1 and the concentration of CSF1, respectively. Using the same procedure as mentioned above, we obtain:

$${}^{q+1}C_M^{(i,j)} = P_M^0\ {}^{q}C_M^{(i,j)} + P_M^1\ {}^{q}C_M^{(i+1,j)} + P_M^2\ {}^{q}C_M^{(i-1,j)} + P_M^3\ {}^{q}C_M^{(i,j+1)} + P_M^4\ {}^{q}C_M^{(i,j-1)}, \tag{9}$$

with coefficients P_M^0, P_M^1, P_M^2, P_M^3 and P_M^4 being defined as follows,

$$
\begin{aligned}
P_M^0 &= 1-\frac{4kD_M}{h^2}-\frac{k\alpha_{MC}}{h^2}\left([C]_{(i+1,j)}^q+[\mathrm{C}]_{(i-1,j)}^q+[C]_{(i,j+1)}^q+[C]_{(i,j-1)}^q-4[C]_{(i,j)}^q\right),\\
P_M^1 &= \frac{kD_M}{h^2}-\frac{k\alpha_{MC}}{4h^2}\left([C]_{(i+1,j)}^q-[C]_{(i-1,j)}^q\right),\\
P_M^2 &= \frac{kD_M}{h^2}-\frac{k\alpha_{MC}}{4h^2}\left([C]_{(i-1,j)}^q-[C]_{(i+1,j)}^q\right),\\
P_M^3 &= \frac{kD_M}{h^2}-\frac{k\alpha_{MC}}{4h^2}\left([C]_{(i,j+1)}^q-[C]_{(i,j-1)}^q\right),\\
P_M^4 &= \frac{kD_M}{h^2}-\frac{k\alpha_{MC}}{4h^2}\left([C]_{(i,j-1)}^q-[C]_{(i,j+1)}^q\right).
\end{aligned}
\tag{10}
$$

S1.3 Text. Tumor cell activities

In the MSABM, tumor cells can either migrate or proliferate. When the internal clock of a tumor cell reaches a specific proliferation time, the cancer cell will proliferate into available space of neighborhoods with equal probability, unless its Moore neighborhood is fully occupied (*34*). Once it reaches a predetermined lifespan or attains a minimal natural death probability, it will transition into a dead cell, awaiting natural degradation by the microenvironment or phagocytosis by M1 macrophages. If there is insufficient space to accommodate a new tumor cell, it will enter a quiescent state. Tumor cells in the quiescent state will immediately transition back to an active state and proliferate when space for proliferation becomes available (Fig. 1H).

Based on our previous PDEs model (*44*), we define the proliferation probability of active tumor cells as follows,

$$
\tilde{p}_{TC} = p_{TC}\left(1+\alpha_{ERK}H_1+\alpha_{AKT}H_2\right)\left(1-\frac{N_{\mathrm{TC}}}{N_{TC}^{\max}}\right). \tag{11}
$$

The proliferation probability of active tumor cells, $\tilde{p}_{TC}(t)$, is influenced by the activations of intracellular kinase proteins ERK and AKT (*51*), where p_{TC} represents the basal proliferation probability of active tumor cells. The coefficients α_{ERK} and α_{AKT} correspond to the regulatory factors related to ERK and AKT, respectively. $H_1(t)=\frac{[ERK](t)}{K_1+[ERK](t)}$ and $H_2(t)=\frac{[AKT](t)}{K_2+[AKT](t)}$ are Hill functions (*76-78*) associated with ERK and AKT, where K_1 and K_2 are Michaelis constants. $N_{TC}(t)$ denotes the number of tumor cells in the TME over time, while $N_{TC}^{\max}$ represents the maximum carrying capacity of TCs within the TME. Note that in the MSABM we use cell count or cell number rather than cell density.

S1.4 Text. Signaling pathways in tumor cells

EGF and IGF1 secreted by M2 macrophages can activate the surface receptors EGFR and IGF1R on glioblastoma cells. Subsequently, the activation of EGFR and IGF1R triggers the downstream pathways

ERK and AKT, which influence the proliferation of glioblastoma cells (*51, 59*). We use a set of ODEs to describe the mechanisms of intracellular signaling pathways in tumor cells (Fig. 1I).

The kinetics of EGFR activation is modeled as follow:

$$\frac{d[EGFR]}{dt}=\frac{V_3[EGF]}{K_{31}+[EGF]}\cdot\frac{1}{1+\frac{[ERK]}{K_{32}}}\cdot\frac{1}{1+\frac{[EGFR\_I]}{K_{33}}}\cdot\left([EGFR]_{\max}-[EGFR]\right)-d_3[EGFR]. \tag{12}$$

In equation (12), $[EGFR]$, $[ERK]$, and $[EGFR\_I]$ represent the concentration of activated EGFR, activated ERK and drug EGFR_I, respectively. $[EGFR]_{\max}$ denotes the maximum concentration of activated EGFR, while V_3 is the maximal activation rate of activated EGFR. K_{31}, K_{32} and K_{33} are Michaelis constants, d_7 denotes the degradation of activated EGFR.

ERK is a transcription factor downstream of the EGFR and IGF1R pathways, influencing the proliferation rate of glioblastoma. The activation of ERK is positively regulated by EGFR and IGF1R, while it is inhibited by AKT. Therefore, the kinetic model of ERK activation is as follows:

$$\frac{d[ERK]}{dt}=\left(1+\frac{V_{41}[EGFR]^n}{K_{41}^n+[EGFR]^n}\right)\cdot\left(1+\frac{V_{42}[IGF1R]}{K_{42}^n+[\mathrm{I}GF1R]}\right)\cdot\frac{V_{43}}{1+\frac{[AKT]}{K_{43}}}\cdot\left([ERK]_{\max}-[ERK]\right)-d_4[ERK]. \tag{13}$$

Correspondingly to the kinetics of EGFR activation, the IGF1R activation is modeled as follows:

$$\frac{d[IGF1R]}{dt}=\frac{V_5[IGF1]}{K_{51}+[IGF1]}\cdot\frac{1}{1+\frac{[ERK]}{K_{52}}}\cdot\frac{1}{1+\frac{[IGF1R\_I]}{K_{53}}}\cdot\left([IGF1R]_{\max}-[IGF1R]\right)-d_5[IGF1R]. \tag{14}$$

Additionally, AKT is also a kinase protein regulated by EGFR and IGF1R, which is modeled as follows:

$$\frac{d[AKT]}{dt}=\frac{V_6[EGFR]}{K_{61}+[EGFR]}\cdot\frac{[IGF1R]}{K_{62}+[IGF1R]}\cdot\left([AKT]_{\max}-[AKT]\right)-d_6[AKT]. \tag{15}$$

The parameters in the above ODEs are estimated from experimental data (*51*) using genetic algorithm (*79*). The results are shown in fig. S1.

S1.5. Macrophage activities

Our model considers three states of TAMs: M0, M1, and M2 (*60, 80*). Initially, macrophages exist in an undifferentiated form within the tissue. As tumor cells proliferate, M0-polariation macrophages are recruited through blood vessels to the vascular cells' Von Neumann neighborhood in the TME (*34, 50*). Subsequently, they polarize into M2 macrophages that promote tumor growth or M1 macrophages that inhibit tumor growth, influenced by CSF1 and the accumulation of CSF1R_I in the TME (*7, 10, 59*).

We assume that the rate at which M0 macrophages are recruited into the TME is related to the number of vascular cells, which is model as follows:

$$N_{M0}(t+k) = N_{M0}(t) + k \cdot p_{rec} N_{VC}\left(1 - \frac{N_M}{N_M^{\max}}\right). \tag{16}$$

where $N_{M0}(t)$, N_{VC} and $N_M(t)$ represent the number of M0 macrophages, vascular cells, and TAMs in all states, respectively, while p_{rec} denotes the basal recruitment probability of M0s. Additionally, time $t = q \cdot k$ and k is the time step size.

For the polarization of TAMs, the probability of M1 transitioning to M2 is defined as:

$$p_{M12}(t) = \alpha_C H_C + \alpha_I H_I, \tag{17}$$

where $H_C(t) = \frac{[CSF1](t)}{K_{M12} + K_{M12}^d [CSF1R\_I](t) + [CSF1](t)}$ and $H_I(t) = \frac{[I](t)}{K_I + [I](t)}$. K_{M12}, K_{M12}^d and K_I are Michaelis constants, while $I(t) = A_0 + S_A \int_0^t [CSF1R\_I](s)ds$ is the integral term from Equation (2). α_C and α_I are adjustment coefficient. Meanwhile, the probability of M2 polarizing to M1, p_{M21}, is set as constant.

Additionally, TAMs in the TME can either remain stationary or migrate in a CSF1-chemoactic manner to empty grid locations in their Von Neumann neighborhood according to the calculated probabilities, with the specific calculation formulas provided in eq. (8-10). M1 macrophages can also phagocytose tumor cells and dead cells in their Von Neumann neighborhoods and subsequently move to and occupy these locations.

S1.6. Removal of dead cells

After tumor cells or macrophages die, they are not immediately cleared from the TME. In addition to being phagocytosed by M1 macrophages (*65*), we assume that dead cells can also decompose at a certain rate. The removal rate $v_d(t, \mathbf{x})$ for dead cells is calculated as follows:

$$v_d(t, \mathbf{x}) = v_0 \left(1 + N_{DC}(t, \mathbf{x})\right) \tag{18}$$

Here, v_0 is the basal removal rate and $N_{DC}(t, \mathbf{x})$ denotes the empty grids in the Moore neighborhood of dead cells in position $\mathbf{x}$ at time t. $N_{DC}(t, \mathbf{x})$ represents the degree of contact between dead cells and the TME. We assume that a larger contact area facilitates easier decomposition. Specifically, as soon as

$\int_{t_0}^{t} v_d(s,\mathbf{x})\,ds \geq 1$ is achieved, the dead cell formed at time t_0 in position $\mathbf{x}$ has fully decomposed at time t. At this point, we can remove the dead cell from the cell grid, leaving behind an empty grid space.

S2. Surrogate model of MSABM

In the second 'informed' part of the M4RL framework, we collect the output information from the first multiscale mathematical modeling part to train a surrogate model with both minimal computing costs and sufficient approximation for rapid prediction. We explained in the main text that the high time cost of performing multiple predictions with the MSABM, along with the substantial computational resources required, makes the MSABM-based optimization quite challenging. To address this challenge, we employ PINN to learn a surrogate model of MSABM by using the MSABM simulations as training data and a SDEs-ODEs hybrid model of cytokines and drugs as physics constrain. The resulting Fokker-Planck equations-based surrogate model reflects the temporal evolution of the probability distribution of tumor density within patient populations from a macroscopic perspective, significantly reducing the time costs associated with predictions under different treatment regimens. This provides an efficient and reliable tool for subsequent reinforcement learning aimed at identifying optimal treatment strategies.

S2.1. MSABM simulations under different treatments as data constraint

We generate 20 in silico treatment regimens of CSF1R_I and IGF1R_I. Subsequently, we input these treatment regimens into the MSABM for multiple predictions, and the output results served as the data constraints for training the PINN. The specific regimens are detailed in fig. S3.

Here, we describe the procedures of simulating the following treatment strategies: combination treatment, continuous treatment, cyclic treatment, adaptive treatment, and cut treatment. We assume that the injected drug dosage is equal to the drug dosage in the bloodstream, and let the normalized concentration in the bloodstream of CSF1R_I and IGF1R_I be represented as $dose_C(t)$ and $dose_I(t)$, then the combination treatment can be expressed as:

$$\begin{cases} dose_C(t) = \dfrac{\kappa_C(t)}{[CSF1R\_I]_{\max}}, \\ dose_I(t) = \dfrac{\kappa_I(t)}{[IGF1R\_I]_{\max}}, \end{cases} \tag{19}$$

where $\kappa_C(t)$ and $\kappa_I(t)$ are positive piecewise functions, representing the tolerable injection dosage of CSF1R_I and IGF1R_I, respectively. Additionally, $[CSF1R\_I]_{\max}$ and $[IGF1R\_I]_{\max}$ denote the maximum tolerable injection doses, which are also the supremum of $\kappa_C(t)$ and $\kappa_I(t)$, respectively.

Similarly, assuming the maximum tolerable injection dosage of the drug is $[drug]_{\max}$, the continuous treatment for the drug over a time interval $(t_1, t_2],\ 0 \leq t_1 < t_2$ can be defined as:

$$dose_{drug}(t) = \frac{\kappa}{[drug]_{\max}}, \tag{20}$$

where $0 < \kappa \le [drug]_{\max}$ is fixed.

Let the period be $T_p > 0$, then in a period, the cyclic treatment can be defined as follows:

$$dose_{drug}(t) = \begin{cases} \dfrac{\kappa_1}{[drug]_{\max}}, \ t \in \left(nT_p, nT_p + t_0\right], \\ \dfrac{\kappa_2}{[drug]_{\max}}, \ t \in \left(nT_p + t_0, (n+1)T_p\right], \end{cases} \tag{21}$$

where $n \in \mathbb{N}, 0 \le \kappa_1 \ne \kappa_2 \le [drug]_{max}$ and $t_0 \in \left(0, T_p\right)$.

Unlike cyclic treatment, which adjusts drug dosages based on time, adaptive treatment is a method that modifies drug dosages or treatment regimens according to individual patient characteristics and real-time feedback. In this case, we select the real-time spatial-average tumor density $\bar{C}_T(t)$ in the TME as the patient characteristic. Using the initial spatial-average tumor density in the TME $\bar{C}_T^0$ as a baseline, a simple adaptive treatment can be represented as follows:

$$dose_{drug}(t) = \begin{cases} \dfrac{\kappa_1}{[drug]_{\max}}, \ \text{while } \bar{C}_T(t) \le m\bar{C}_T^0, \\ \dfrac{\kappa_2}{[drug]_{\max}}, \ \text{while } \bar{C}_T(t) > m\bar{C}_T^0, \end{cases} \tag{22}$$

where $0 \le \kappa_1 \ne \kappa_2 \le [drug]_{\max}$. This treatment regimen is determined by a single hyperparameter $m > 0$, which sets the threshold for switching the drug dosage. The advantage of this approach is its simplicity and clarity in drug control. However, the ideal scenario would involve frequently switching the drug dosage to make $\bar{C}_T(t)$ approach $m\bar{C}_T^0$. In practice, frequent changes in drug dosages are not feasible in clinical settings. Another form of adaptive treatment is controlled by two hyperparameters $m_2 > m_1 > 0$ that govern the switching of drug dosages:

$$dose_{drug}(t) = \begin{cases} \dfrac{\kappa_1}{[drug]_{\max}}, \ \text{until } \bar{C}_T(t) \le m_1\bar{C}_T^0, \\ \dfrac{\kappa_2}{[drug]_{\max}}, \ \text{until } \bar{C}_T(t) > m_2\bar{C}_T^0, \end{cases} \tag{23}$$

where $0 \le \kappa_1 \ne \kappa_2 \le [drug]_{\max}$. This adaptive schedule has been used in pilot clinical trials (*81, 82*), as it can more feasibly maintain the continuous use of the same concentration of the drug over an extended period, while also make $\bar{C}_T(t)$ tend to the range $\left(m_1\bar{C}_T^0, m_2\bar{C}_T^0\right)$.

Cut treatment, or more commonly referred to as fixed-duration treatment, is a regimen defined as follows:

$$dose_{drug}(t)=\begin{cases}\dfrac{\kappa_1}{[drug]_{\max}}, & t\le t_0,\\ \dfrac{\kappa_2}{[drug]_{\max}}, & t>t_0,\end{cases} \tag{24}$$

where $0\le\kappa_1\ne\kappa_2\le[drug]_{\max}$ and $t_0>0$.

S2.2. SDEs-ODEs hybrid model for MSABM simplification

If we only concern cell density of tumor cells for prediction purpose, we sought to simplify the spatial dimension of the model. In the previously established MSABM, the migration of tumor cells and macrophages, as well as the diffusion of cytokines and drug factors within the microenvironment, is based on PDEs (*44*). Considering that the diffusion and chemotaxis terms do not affect the total density of tumor cells within the TME, we simplified the PDEs related to cell dynamics and the PDEs associated with factor diffusion into SDEs and ODEs, respectively. The equation (4) with $\tilde{d}_{TC}=d_{TC}\left(1+d_{TC}^{M1}C_{M1}\right)$ describes the spatiotemporal evolution of tumor cells, where d_{TC} denotes the baseline death rate of TCs and d_{TC}^{M1} represents the phagocytic action of M1 macrophages on TCs. As such, we utilized a SDE to describe the temporal evolution of the normalized tumor cell density $C_{TC}^{*}(t)$ within the TME as follows:

$$\frac{dC_{TC}^{*}}{dt}=k_1^{*}\tilde{r}_{TC}^{*}C_{TC}^{*}-k_2^{*}\tilde{d}_{TC}^{*}C_{TC}^{*}, \tag{25}$$

where $\tilde{r}_{TC}^{*}$ is related to the intracellular signaling pathways and the maximum carrying capacity of tumor cells within the TME:

$$\tilde{r}_{TC}^{*}(t)=r_{TC}\left(1+k_3^{*}H_1^{*}+k_4^{*}H_2^{*}\right)\left(1-C_{TC}^{*}\right). \tag{26}$$

For convenience, we simplified the modulation of signaling pathways on the cell proliferation by directly connecting the average concentrations of EGF and IGF1 to the tumor cell proliferation rate using Hill functions in the surrogate model, i.e., $H_1^{*}(t)=\dfrac{[E]_{avg}}{k_5^{*}K_1^{*}+[E]_{avg}}$ and $H_2^{*}(t)=\dfrac{[I]_{avg}}{k_6^{*}K_2^{*}+[I]_{avg}+k_7^{*}[IGF1R\_I]_{avg}}$. Here, K_1^{*} and K_2^{*} are Michaelis constants, while $k_i^{*}, i\in\mathbb{N}^{+}$ represent the adjustment parameters required when adapting to the surrogate model. The same applies to the follows, parameters and variables marked with an $*$ like $k_i^{*}, i\in\mathbb{N}^{+}$ and $\tilde{r}_{TC}^{*}$ all serve in the surrogate model, as well as $[\cdot]_{avg}$ represents the average concentrations of factors in virtual TME. Due to the spatial heterogeneity caused by tumor cell division and the migration of M1 macrophages within the MSABM, the phagocytic efficiency of M1 macrophages toward TCs exhibits randomness. Therefore, we incorporated Gaussian white noise $\varepsilon(t)$ in the modeling of TCs death rate:

$$\tilde{d}_{TC}^{*}(t) = d_{TC}\left(1 + k_8^* d_{TC}^{M1}\left(1 + \sigma_{TC}^{*} \cdot \varepsilon(t)\right) C_{M1}^{*}\right), \tag{27}$$

where σ_{TC}^{*} is the noise diffusion rate associated with tumor cells.

Assuming that the number of M0 macrophages in the TME of the developed tumor is very low, we only consider the cell population dynamics of M1 and M2 macrophages in the surrogate model. The PDEs describing the evolutions of densities of M1 and M2 macrophages are as follows (*44*):

$$\begin{cases} \dfrac{\partial C_{M1}}{\partial t} = D_M \Delta C_{M1} - \alpha_{MC} \nabla\left(C_{M1} \nabla [C]\right) + \alpha_{M21} C_{M2} - C_{M1}\left(\alpha_C H_C + \alpha_I H_I\right), \\ \dfrac{\partial C_{M2}}{\partial t} = D_M \Delta C_{M2} - \alpha_{MC} \nabla\left(C_{M2} \nabla [C]\right) - \alpha_{M21} C_{M2} + C_{M1}\left(\alpha_C H_C + \alpha_I H_I\right), \end{cases} \tag{28}$$

where α_{M21} represents the fixed rate of M2 macrophages polarizing to M1 state. $\alpha_C H_C + \alpha_I H_I$ is similar to that in eq. (17); while in eq. (17), it represents a probability, but here it denotes a rate. Additionally, the remaining parameters and variables are the same as those in eq. (17). Similar to the simplification of tumor cell dynamics as mentioned above, we transformed the above PDEs of macrophages to the SDEs. Due to the total number of TAMs in the TME approaches the environmental carrying capacity and that there are almost no undifferentiated macrophages, we make the following assumption: $C_{M1}^{*} + C_{M2}^{*} = 1$ (*4, 50*). Additionally, the polarization between M1 and M2 macrophages is influenced by drug penetration from blood vessels and their own spatial heterogeneity, introducing randomness. Therefore, the evolution equation for the M1 macrophages in the TME is as follows:

$$\frac{dC_{M1}^{*}}{dt} = k_9^* \alpha_{M21}\left(1 - C_{M1}^{*}\right) - k_{10}^{*}\left(1 + \sigma_M^{*} \cdot \varepsilon(t)\right) C_{M1}^{*}\left(k_{11}^{*} \alpha_C H_C^{*} + k_{12}^{*} \alpha_I H_I^{*}\right), \tag{29}$$

where σ_M^{*} denotes the noise diffusion rate associated with TAMs. Meanwhile, we have $H_C^{*}(t) = \dfrac{[C]_{avg}}{k_{13}^{*} K_{M12} + k_{14}^{*} K_{M12}^{d} [CSF1R\_I]_{avg} + [C]_{avg}}$, $H_I^{*}(t) = \dfrac{I^{*}}{k_{15}^{*} K_I + I^{*}}$, as well as $I^{*}(t) = A_0 + k_{16}^{*} S_A \int_0^t [CSF1R\_I]_{avg}(s) ds$.

For the cytokines in the TME, we refer to eq. (1) except diffusion term and describe them using their average concentrations by ODEs as follows:

$$
\begin{cases}
\dfrac{d[I]_{avg}}{dt} = k_{17}^* I^* S_{IGF1}^{M2} C_{M2}^* \left(1 - \dfrac{[I]_{avg}}{k_{18}^* [I]_{\max}}\right) - k_{19}^* d_{IGF1} [I]_{avg} , \\
\dfrac{d[E]_{avg}}{dt} = k_{20}^* S_{EGF}^{M2} C_{M2}^* \left(1 - \dfrac{[E]_{avg}}{k_{21}^* [E]_{\max}}\right) - k_{22}^* d_{EGF} [E]_{avg} , \\
\dfrac{d[C]_{avg}}{dt} = k_{23}^* S_{CSF1}^{TC} C_{TC}^* \left(1 - \dfrac{[C]_{avg}}{k_{24}^* [C]_{\max}}\right) - k_{25}^* d_{CSF1} [C]_{avg} .
\end{cases} \tag{30}
$$

Similarly, we use ODEs and refer to eq. (3) in text S1.1 to model the temporal changes in average concentrations of drug factors CSF1R_I and IGF1R_I as follows:

$$
\begin{cases}
\dfrac{d[CSF1R\_I]_{avg}}{dt} = k_{26}^* q_{CSF1R\_I} \left(dose_{CSF1R\_I} - [CSF1R\_I]_{avg}\right) \\
\qquad - k_{27}^* \eta_{CSF1R\_I} [CSF1R\_I]_{avg} - k_{28}^* u_{CSF1R\_I} \left(C_{M1}^* + C_{M2}^*\right)[CSF1R\_I]_{avg} , \\
\dfrac{d[IGF1R\_I]_{avg}}{dt} = k_{29}^* q_{IGF1R\_I} \left(dose_{IGF1R\_I} - [IGF1R\_I]_{avg}\right) \\
\qquad - k_{30}^* \eta_{IGF1R\_I} [IGF1R\_I]_{avg} - k_{31}^* u_{IGF1R\_I} \left(C_{TC}^*\right)[IGF1R\_I]_{avg} .
\end{cases} \tag{31}
$$

Notably, the representations and values of non-starred parameters in this subsection (text S2.2) are identical as those in text S1, and are also listed in table S2.

S2.3. Fokker-Planck equations for physical constraint

We described in the main text that we transform SDEs into Fokker-Planck equations to describe the evolution of the probability distribution of cell density at the population level. After transformation, the Fokker-Planck form of eq. (25) is as follows:

$$
\begin{aligned}
\frac{\partial \mathcal{P}_{TC}\left(t, C_{TC}^*\right)}{\partial t} = &-\frac{\partial}{\partial C_{TC}^*}\left[\mu_{TC}\left(t, C_{TC}^*\right)\mathcal{P}_{TC}\left(t, C_{TC}^*\right)\right] \\
&+ \frac{1}{2}\frac{\partial^2}{\partial {C_{TC}^*}^2}\left[\nu_{TC}\left(t, C_{TC}^*\right)\mathcal{P}_{TC}\left(t, C_{TC}^*\right)\right],
\end{aligned} \tag{32}
$$

where $\mathcal{P}_{TC}\left(t, C_{TC}^*\right)$ is the p.d.f. of the random variable C_{TC}^* at time t. $\mu_{TC}\left(t, C_{TC}^*\right) = k_1^* \tilde{r}_{TC}^* C_{TC}^* \left(1 - C_{TC}^*\right) - k_2^* d_{TC} \left(1 + k_8^* d_{TC}^{M1} C_{M1}^*\right) C_{TC}^*$ is the drift coefficient and $\nu_{TC}\left(t, C_{TC}^*\right) = -k_2^* d_{TC} k_8^* d_{TC}^{M1} C_{M1}^* C_{TC}^* \sigma_{TC}$ is the diffusion coefficient. Additionally, using the same method, the Fokker-Planck equation obtained from eq. (29) is as follows:

$$\frac{\partial \mathcal{P}_{M1}\left(t, C_{M1}^*\right)}{\partial t} = -\frac{\partial}{\partial C_{M1}^*}\left[\mu_{M1}\left(t, C_{M1}^*\right)\mathcal{P}_{M1}\left(t, C_{M1}^*\right)\right] + \frac{1}{2}\frac{\partial^2}{\partial {C_{M1}^*}^2}\left[\nu_{M1}\left(t, C_{M1}^*\right)\mathcal{P}_{M1}\left(t, C_{M1}^*\right)\right], \tag{33}$$

where $\mathcal{P}_{M1}\left(t, C_{M1}^*\right)$ is the p.d.f. of the random variable C_{M1}^* at time t.
$\mu_{M1}\left(t, C_{M1}^*\right) = k_9^*\alpha_{M21}\left(1 - C_{M1}^*\right) - k_{10}^* C_{M1}^*\left(k_{11}^*\alpha_C H_C^* + k_{12}^*\alpha_I H_I^*\right)$ is the drift coefficient and
$\nu_{M1}\left(t, C_{M1}^*\right) = -k_{10}^* C_{M1}^*\sigma_M\left(k_{11}^*\alpha_C H_C^* + k_{12}^*\alpha_I H_I^*\right)$ is the diffusion coefficient.

Thus, the ODEs (eq. (30) and (31)) and the Fokker-Planck equations (eq. (32) and (33)) constitute a new hybrid model, which is referred to as Fokker-Planck equations-based surrogate model in this study, that can be used to describe the temporal dynamics of average concentrations of cytokines/drug factors in the TME, as well as the evolutionary characteristics of cell densities at the populations level.

S2.4. ODEs solutions

The ODEs (eq. (30) and (31)) can be solved analytically. Taking the ODE related to $[C]_{avg}$ as an example, by applying variable separation, we obtain:

$$\frac{1}{a - b[C]_{avg}} d[C]_{avg} = dt,$$

where $a = k_{23}^* S_{CSF1}^T C_T^*$ and $b = \frac{a}{k_{24}^*[C]_{\max}} + k_{25}^* d_{CSF1}$. Integrating both sides of the equation yields:

$$-\int \frac{1}{b[C]_{avg} - a} d[C]_{avg} = \int dt.$$

Then, we have

$$-\frac{\ln\left(b[C]_{avg} - a\right)}{b} = t + K,$$

where K is a constant. Assuming the initial condition is $[C]_{avg}\Big|_{t=t_0} = [C]_{avg}^{t_0}$, continuing with the calculation yields:

$$[C]_{avg} = \left([C]_{avg}^{t_0} - \frac{a}{b}\right)e^{-bt} + \frac{a}{b}. \tag{34}$$

Similarly, we can analytically solve ODEs for $[E]_{avg}$, $[I]_{avg}$, $[CSF1R\_I]_{avg}$ and $[IGF1R\_I]_{avg}$.

S2.5. PINN formulation

Generally, a multi-dimensional spatio-temporal system can be characterized by a set of nonlinear coupled parameterized PDEs as follows (*69*):

$$\mathbf{u}_t + \mathcal{F}\left[\mathbf{u}, \nabla_x \mathbf{u}, \nabla_x^2 \mathbf{u}; \boldsymbol{\lambda}\right] = \mathbf{s}, \tag{35}$$

where $\boldsymbol{u} = \boldsymbol{u}(t, \boldsymbol{x}) \in \mathbb{R}^{1 \times n}$ is the *n*-dimensional latent solution. $\mathbf{u}_t$ and $\nabla_{\mathbf{x}}\mathbf{u}$ are time derivative term and spatial gradient term, respectively. $\mathcal{F}[\ \cdot\ ;\boldsymbol{\lambda}]$ denotes the nonlinear functional of $\mathbf{u}$ and its spatial derivatives and is parameterized by $\boldsymbol{\lambda}$. $\mathbf{s}(t, \mathbf{x})$ represents the source input to the system. Additionally, the initial and boundary conditions of eq. (35) can be denoted as $\mathcal{I}\left[\mathbf{x} \in \Omega, t = 0; \mathbf{u}, \mathbf{u}_t\right] = 0$ and $\mathcal{B}\left[\mathbf{x} \in \partial\Omega; \mathbf{u}, \nabla_{\mathbf{x}}\mathbf{u}\right] = 0$. Then, to demonstrate a specific dimension of the system, we can define $f(t, \mathbf{x})$ as

$$f := u_t + \mathcal{F}\left[u, \nabla_x u, \nabla_x^2 u; \boldsymbol{\lambda}\right], \tag{36}$$

where $u = u(t, \boldsymbol{x}) \in \mathbb{R}$ is the latent solution. Assuming that a deep neural network (DNN) $u^{\boldsymbol{\theta}}(t, \mathbf{x}; \boldsymbol{\theta})$ can be established to approximate $u(t, \mathbf{x})$, where $\boldsymbol{\theta}$ is the set of trainable parameters in the DNN. Thus, $f(t, \mathbf{x})$ is call as a physics-informed neural network (*70*). Notably, chain rule and automatic differentiation (*83*) can be applied in the derivation of $f(t, \mathbf{x})$. Meanwhile, the parameters (PDE coefficients) $\boldsymbol{\lambda}$ in $\mathcal{F}[\ \cdot\ ;\boldsymbol{\lambda}]$ are also the trainable parameters of the PINN. The optimal parameter sets $\hat{\boldsymbol{\theta}}$ and $\hat{\boldsymbol{\lambda}}$ can be estimated by minimizing the following loss function:

$$\mathcal{L}\left(\boldsymbol{\theta}, \boldsymbol{\lambda}; \mathcal{D}_u, \mathcal{D}_c\right) = \beta_1 \mathcal{L}_d\left(\boldsymbol{\theta}; \mathcal{D}_u\right) + \beta_2 \mathcal{L}_p\left(\boldsymbol{\theta}, \boldsymbol{\lambda}; \mathcal{D}_c\right), \tag{37}$$

where $\mathcal{D}_u = \left\{\left(t_i, \mathbf{x}_i\right) \middle| i = 1, \ldots, N_u\right\}$ and $\mathcal{D}_c = \left\{\left(t_i, \mathbf{x}_i\right) \middle| i = 1, \ldots, N_c\right\}$ represent the prior point set for data-driven constraints and randomly sampled point set for physical-driven constraints in the spatio-temporal space. β_1, β_2 is the adjustment coefficient. The mean data loss function $\mathcal{L}_d$ and the mean residual physics loss function $\mathcal{L}_p$ are defined as:

$$\mathcal{L}_d\left(\boldsymbol{\theta}; \mathcal{D}_u\right) = \frac{1}{N_u} \sum_{i=1}^{N_u} \left(u^{\boldsymbol{\theta}}\left(t_i, \mathbf{x}_i; \boldsymbol{\theta}\right) - u\left(t_i, \mathbf{x}_i\right)\right)^2, \tag{38}$$

with $\left(t_i, \mathbf{x}_i\right) \in \mathcal{D}_u$, and

$$\mathcal{L}_p\left(\boldsymbol{\theta}, \boldsymbol{\lambda}; \mathcal{D}_c\right) = \frac{1}{N_c} \sum_{i=1}^{N_c} \left(f\left(t_i, \mathbf{x}_i; \boldsymbol{\theta}, \boldsymbol{\lambda}\right) - s\left(t_i, \mathbf{x}_i\right)\right)^2, \tag{39}$$

with $\left(t_i, \mathbf{x}_i\right) \in \mathcal{D}_c$, where $s(t, \mathbf{x})$ represents the source input.

S2.6. Surrogate model training

As mentioned above, the equations eq. (30-33) constitute a surrogate model, with parameter vectors $\mathbf{k}_{TC}^* = (k_1^*, \ldots, k_8^*)$, $\mathbf{k}_{M1}^* = (k_9^*, \ldots, k_{15}^*)$, $\mathbf{k}_W^* = (k_{16}^*, \ldots, k_{31}^*)$ and $\boldsymbol{\sigma}^* = (\sigma_{TC}^*, \sigma_M^*)$ representing the parameters related to tumor cells, M1 macrophages, factors in TME, and the noise diffusion rates, respectively. Below we describe how to train the surrogate model.

In the first step, referring to text S2.1, we selected 20 different combination treatments and input them into the MSABM for multiple predictions (N = 100 and T = 200 days). We recorded the average concentrations of CSF1, EGF, IGF1, CSF1R_I, and IGF1R_I in the TME at time $t_i \in \mathcal{D}_t = \{n \cdot \triangle t | n \in \mathbb{N}, n \cdot \triangle t < T\}$ with $\triangle t = 0.5$ hour denotes the time step in MSABM. Then, we gathered the time-varying curves of tumor cell density and M1 macrophage density in the TME for the N individuals and converted them into the probability distribution of cell density at time $t_i \in \mathcal{D}_t$.

In the second step, we denoted $[W]_{avg}$ to represent the average concentration of a certain factor in the TME at time $t_i \in \mathcal{D}_t$ and defined the mean residual loss function associated with the certain factor W as:

$$\mathcal{L}_W\left(\mathbf{k}_W^*; \mathcal{D}_t\right) = \frac{1}{|\mathcal{D}_t|} \sum_{t_i \in \mathcal{D}_t} \left([W]^{\mathbf{k}}\left(t_i; \mathbf{k}_W^*\right) - [W]_{avg}\left(t_i\right) \right)^2, \tag{40}$$

where $[W]^{\mathbf{k}}\left(t; \mathbf{k}_W^*\right)$ is calculated by eq. (34) and the initial conditions are listed in table S3. Then, we estimated $\mathbf{k}_W^* = (k_{16}^*, \ldots, k_{31}^*)$ by minimizing the total mean residual loss functions derived from factors CSF1, EGF, IGF1, CSF1R_I, and IGF1R_I.

In the third step, we fixed the trained parameters $\mathbf{k}_W^* = (k_{16}^*, \ldots, k_{31}^*)$ in the second step and used the calculated average densities to estimate $\mathbf{k}_{TC}^* = (k_1^*, \ldots, k_8^*)$ and $\mathbf{k}_{M1}^* = (k_9^*, \ldots, k_{15}^*)$. Viewing equations eq. (25) and eq. (29) as ODEs with $\boldsymbol{\sigma}^* = \mathbf{0}$, we defined $f_{TC} = \frac{du_{TC}}{dt} - k_1^* \tilde{r}_{TC}^* u_{TC} + k_2^* \tilde{d}_{TC}^* u_{TC}$ and $f_{M1} = \frac{du_{M1}}{dt} - k_9^* \alpha_{M21} u_{M1} + k_{10}^* u_{M1} \left(k_{11}^* \alpha_C H_C^* + k_{12}^* \alpha_I H_I^*\right)$, where $u_{TC}\left(t; \boldsymbol{\theta}_{TC}\right)$ and $u_{M1}\left(t; \boldsymbol{\theta}_{M1}\right)$ represent the DNNs aiming to approximate C_{TC}^* and C_{M1}^*, respectively, $\boldsymbol{\theta}_{TC}$ and $\boldsymbol{\theta}_{M1}$ are trainable parameters in the DNNs. The loss function corresponding to f_{TC} is defined as

$$\mathcal{L}_{TC}\left(\boldsymbol{\theta}_{TC}, \mathbf{k}_{TC}^*; \mathcal{D}_t, \mathcal{D}_c^{TC}\right) = \mathcal{L}_d^{TC}\left(\boldsymbol{\theta}_{TC}; \mathcal{D}_t\right) + \mathcal{L}_p^{TC}\left(\boldsymbol{\theta}_{TC}, \mathbf{k}_{TC}^*; \mathcal{D}_c^{TC}\right), \tag{41}$$

where $\mathcal{D}_t = \{n \cdot \triangle t | n \in \mathbb{N}, n \cdot \triangle t < T\}$ has defined earlier and $\mathcal{D}_c^{TC} \subset [0, T]$ is randomly sampled point set. Meanwhile, $\mathcal{L}_d^{TC}\left(\boldsymbol{\theta}_{TC}; \mathcal{D}_t\right) = \frac{1}{|\mathcal{D}_t|} \sum_{t_i \in \mathcal{D}_t} \left(u_{TC}\left(t_i; \boldsymbol{\theta}_{TC}\right) - C_{TC}^{avg}\left(t_i\right)\right)^2$ and $\mathcal{L}_p^{TC}\left(\boldsymbol{\theta}_{TC}, \mathbf{k}_{TC}^*; \mathcal{D}_c^{TC}\right) = \frac{1}{|\mathcal{D}_c^{TC}|} \sum_{t_i \in \mathcal{D}_c^{TC}} \left(f_{TC}\left(t_i; \boldsymbol{\theta}_{TC}, \mathbf{k}_{TC}^*\right)\right)^2$ with C_{TC}^{avg} representing the average tumor cells density across the N individuals. Similarly, the loss function of f_{M1} is defined as

$$\mathcal{L}_{M1}\left(\boldsymbol{\theta}_{M1},\mathbf{k}_{M1}^{*};\mathcal{D}_t,\mathcal{D}_c^{M1}\right)=\mathcal{L}_d^{M1}\left(\boldsymbol{\theta}_{M1};\mathcal{D}_t\right)+\mathcal{L}_p^{M1}\left(\boldsymbol{\theta}_{M1},\mathbf{k}_{M1}^{*};\mathcal{D}_c^{\mathrm{M1}}\right), \tag{42}$$

where $\mathcal{D}_t = \{n\cdot\triangle t | n\in\mathbb{N}, n\cdot\triangle t < T\}$ has defined earlier and $\mathcal{D}_c^{M1}\subset[0,T]$ is randomly sampled point set. Meanwhile, $\mathcal{L}_d^{M1}\left(\boldsymbol{\theta}_{TC};\mathcal{D}_t\right)=\frac{1}{|\mathcal{D}_t|}\sum_{t_i\in\mathcal{D}_t}\left(u_{M1}\left(t_i;\boldsymbol{\theta}_{M1}\right)-[M1]_{avg}\right)^2$ and $\mathcal{L}_p^{M1}\left(\boldsymbol{\theta}_{M1},\mathbf{k}_{M1}^{*};\mathcal{D}_c^{\mathrm{M1}}\right)=\frac{1}{\left|\mathcal{D}_c^{M1}\right|}\sum_{t_i\in\mathcal{D}_c^{M1}}\left(f_{M1}\left(t_i;\boldsymbol{\theta}_{M1},\mathbf{k}_{M1}^{*}\right)\right)^2$ with $[M1]_{avg}$ represents the average cell density of M1 macrophages across the N individuals.

In the last step, we fixed the trained parameters $\mathbf{k}_{TC}^{*}=\left(k_1^{*},\ldots,k_8^{*}\right)$, $\mathbf{k}_{M1}^{*}=\left(k_9^{*},\ldots,k_{15}^{*}\right)$ and $\mathbf{k}_{W}^{*}=\left(k_{16}^{*},\ldots,k_{31}^{*}\right)$ in the above second and third steps. Taking into account that the variations in the evolution of average M1 macrophage density in the TME, as output by the MSABM, are relatively small under different combination treatments. and considering that the subsequent RL only requires the evolution of tumor cell probability density to drive the environment, we only approximated the solution specifically for the Fokker-Planck equation related to TCs. We defined the PINN as $f_{TC}^{\mathcal{P}}=\frac{\partial u_{TC}^{\mathcal{P}}}{\partial t}+\frac{\partial}{\partial C_{TC}^{*}}\left(\mu_{TC}\cdot u_{TC}^{\mathcal{P}}\right)-\frac{1}{2}\frac{\partial^2}{\partial C_{TC}^{*\,2}}\left(\nu_{TC}\cdot u_{TC}^{\mathcal{P}}\right)$ with $u_{TC}^{\mathcal{P}}\left(t,C_{TC}^{*};\boldsymbol{\theta}_{TC}^{\mathcal{P}}\right)\approx\mathcal{P}_{TC}\left(t,C_{TC}^{*}\right)$ representing the DNN, where $\boldsymbol{\theta}_{TC}^{\mathcal{P}}$ are the trainable parameters. The loss function of PINN $f_{TC}^{\mathcal{P}}$ is defined as

$$\mathcal{L}_{TC}^{\mathcal{P}}\left(\boldsymbol{\theta}_{TC}^{\mathcal{P}},\boldsymbol{\sigma}^{*};{}^{\mathcal{P}}\mathcal{D}_u^{TC},{}^{\mathcal{P}}\mathcal{D}_c^{TC}\right)={}^{\mathcal{P}}\mathcal{L}_d^{TC}\left(\boldsymbol{\theta}_{TC}^{\mathcal{P}};{}^{\mathcal{P}}\mathcal{D}_u^{TC}\right)+{}^{\mathcal{P}}\mathcal{L}_p^{TC}\left(\boldsymbol{\theta}_{TC}^{\mathcal{P}},\boldsymbol{\sigma}^{*};{}^{\mathcal{P}}\mathcal{D}_c^{TC}\right), \tag{43}$$

where ${}^{\mathcal{P}}\mathcal{D}_u^{TC}=\left\{\left(t_i,c_i\right)\middle| i=1,\ldots,{}^{\mathcal{P}}N_u^{TC}\right\}$ and ${}^{\mathcal{P}}\mathcal{D}_c^{TC}=\left\{\left(t_i,c_i\right)\middle| i=1,\ldots,{}^{\mathcal{P}}N_c^{TC}\right\}$ represent the prior point set and randomly sampled point set in the domain $[0,T]\times[0,1]$. Meanwhile, ${}^{\mathcal{P}}\mathcal{L}_d^{TC}\left(\boldsymbol{\theta}_{TC}^{\mathcal{P}};{}^{\mathcal{P}}\mathcal{D}_u^{TC}\right)=\frac{1}{{}^{\mathcal{P}}N_u^{TC}}\sum_{i=1}^{{}^{\mathcal{P}}N_u^{TC}}\left(u_{TC}^{\mathcal{P}}\left(t_i,c_i;\boldsymbol{\theta}_{TC}^{\mathcal{P}}\right)-\mathcal{P}_{TC}\left(t_i,c_i\right)\right)^2$ and ${}^{\mathcal{P}}\mathcal{L}_p^{TC}\left(\boldsymbol{\theta}_{TC}^{\mathcal{P}},\boldsymbol{\sigma}^{*};{}^{\mathcal{P}}\mathcal{D}_c^{TC}\right)=\frac{1}{{}^{\mathcal{P}}N_c^{TC}}\sum_{i=1}^{{}^{\mathcal{P}}N_c^{TC}}\left(f_{TC}^{\mathcal{P}}\left(t_i,c_i;\boldsymbol{\theta}_{TC}^{\mathcal{P}},\boldsymbol{\sigma}^{*}\right)\right)^2$ with $\mathcal{P}_{TC}\left(t_i,c_i\right)$ representing the abovementioned tumor cells probability density at time t_i and density c_i derived from the MSABM simulations.

S3. Reinforcement learning

In the final ‘reinforcement learning’ part of the M4RL framework, we utilized the learned efficient Fokker-Planck equations-based surrogate model to drive RL for predicting optimal scheduling of combination treatments. We will first introduce the basic formulations of RL and the A3C algorithm. Following that, we will present the design of the reward function for the RL and outline the steps for training the actor-critic networks to explore the optimal treatment.

S3.1. RL background

Although we have already introduced the standard RL process in the main text, it is helpful to reiterate the common setups and their corresponding symbols and representations in table S4. Generally, there are three methods for RL, with the first being value-based method that uses an approximator (e.g., a neural network) to represent the action value function $Q(s,a)$. The approximator $Q(s,a;\boldsymbol{\theta})$ is parameterized by trainable parameters $\boldsymbol{\theta}$, which can be updated through several RL algorithms. For instance, Q-learning is a model-free offline RL algorithm aimed at directly approximating the optimal action value function (*84*), that is, $Q^{*}(s,a) \approx Q(s,a;\boldsymbol{\theta})$. The update rule for the $Q(s,a)$ in One-step Q-learning is as follows:

$$Q(s,a) \leftarrow Q(s,a) + \alpha\left[r(s,a) + \gamma \max_{a'} Q(s',a') - Q(s,a)\right], \tag{44}$$

while all the symbols can be found in table S4. The parameters $\boldsymbol{\theta}$ are learned by minimizing a series of loss functions, with the ith loss function is defined as (*53*):

$$\mathcal{L}_i(\theta_i) = \mathbb{E}\left(r(s,a) + \gamma \max_{a'} Q(s',a';\theta_i) - Q(s,a;\theta_{i-1})\right). \tag{45}$$

Policy-based method is also important in RL. For example, the policy gradient method directly optimizes the parameters in the policy $\pi(a|s;\boldsymbol{\theta})$ by maximizing the expected return to learn the optimal policy. The target function that needs to be approximated is:

$$\mathcal{J}(\boldsymbol{\theta}) = \mathbb{E}\left[R_t | s_t, a_t, \pi(a_t|s_t;\boldsymbol{\theta})\right], \tag{46}$$

where the gradient ascent method is basically used for updating parameters as:

$$\boldsymbol{\theta} \leftarrow \boldsymbol{\theta} + \alpha \nabla_{\boldsymbol{\theta}} \mathcal{J}(\boldsymbol{\theta}). \tag{47}$$

Moreover, utilizing the unbiased estimate of $\mathcal{J}(\boldsymbol{\theta})$ for the parameters updating, i.e. $\boldsymbol{\theta} \leftarrow \boldsymbol{\theta} + \alpha \nabla_{\boldsymbol{\theta}} \log \pi(a_t|s_t;\boldsymbol{\theta}) R_t$, is more accurate over a large number of samples, and it can further reduce the variance of the estimate by employing the following (*85*):

$$\boldsymbol{\theta} \leftarrow \boldsymbol{\theta} + \alpha \nabla_{\boldsymbol{\theta}} \log \pi(a_t|s_t;\boldsymbol{\theta})\left(R_t - b_t(s_t)\right), \tag{48}$$

where $b_t(s_t)$ is a learned baseline function from the state.

Third, the actor-critic method (*86*), where the actor represents the policy $\pi(a|s;\boldsymbol{\theta})$ and the critic serves as the baseline $b_t(s_t) \approx V_\pi(s_t)$, effectively balances exploration and exploitation by simultaneously learning the optimal policy and estimating the value function. Considering R_t and b_t are estimates of $Q_\pi(s_t, a_t)$ and $V_\pi(s_t)$, the advantage function can be defined as follows:

$$A(s,a)=Q(s,a)-V(s), \tag{49}$$

and we can use $R_t - b_t$ to measure the benefit of taking an action in a given state (*53*).

S3.2. A3C algorithm

The asynchronous advantage actor-critic algorithm (*53*) consists of a global network that allows for asynchronous updates during training through multiple processes. This approach helps alleviates the need for specialized architecture for GPU-based deep learning algorithm and reduces the computational costs (*72*). The algorithm operates in a forward view, updating the parameters $\boldsymbol{\theta} = (\boldsymbol{\theta}_\pi, \boldsymbol{\theta}_v)$ in the global network with the cumulative gradient when the local process has completed a fixed n steps or reached a terminal state as follows:

$$\begin{cases} \boldsymbol{\theta}_\pi \leftarrow \boldsymbol{\theta}_\pi + \alpha \sum_t \nabla_{\boldsymbol{\theta}_\pi} \log \pi\left(a_t \middle| s_t; \boldsymbol{\theta}_\pi^{'}\right) A\left(a_t \middle| s_t; \boldsymbol{\theta}_v^{'}\right), \\ \boldsymbol{\theta}_v \leftarrow \boldsymbol{\theta}_v + \alpha \sum_t \dfrac{\partial \left(R_t - V\left(s_t; \boldsymbol{\theta}_v^{'}\right)\right)^2}{\partial \boldsymbol{\theta}_v^{'}}. \end{cases} \tag{50}$$

Here, $\boldsymbol{\theta}_\pi$ and $\boldsymbol{\theta}_v$ are the trainable parameters in global policy network and global value network, respectively, while $\boldsymbol{\theta}_\pi^{'}$ and $\boldsymbol{\theta}_v^{'}$ are local network parameters in a certain process. Additionally, $A\left(a_t \middle| s_t; \boldsymbol{\theta}_v^{'}\right)$ is the advantage function defined as follows：

$$A\left(a_t \middle| s_t; \boldsymbol{\theta}_v^{'}\right) = \sum_{i=0}^{k-1} \gamma^i r_{t+i} + \gamma^k V\left(s_{t+k}; \boldsymbol{\theta}_v^{'}\right) - V\left(s_t; \boldsymbol{\theta}_v^{'}\right), \tag{51}$$

where $\sum_{i=0}^{k-1} \gamma^i r_{t+i}$ is the return R_t. Notably, we use $\sum_{i=0}^{k-1} \gamma^i r_{t+i}$ here to emphasis the variable $k \le n$, which varies with s_t and represents the remaining time steps from time t to the parameters updating time.

S3.3. Reward function

The A3C algorithm, based on the surrogate model, guides the policy $\pi(a|s)$ updates by optimizing the value function $V_\pi(s)$. At each time step, the environment $\mathcal{E}$ provides an immediate reward $r_t(s_t, a_t)$ based on the policy $\pi(a_t|s_t)$. The design of the reward function adheres to two main points: first, to increase the survival rate of the patient population, and second, to minimize the dosage of drugs as much as possible. The pseudocode of the reward function is given in algorithm S1, and the corresponding variables and parameters are detailed in table S5.

S3.4. Surrogate model-based A3C network training steps

As shown in Fig. 6 from the main text, during the training, we first extract a time series from the environment guided by the surrogate model, which consists of two dimensions: the current population survival probability and the change in survival probability. This information collectively forms the state,

while the reward is calculated based on the defined reward function. After feature extraction using a Long Short-Term Memory (LSTM) model, the state is represented as a four-dimensional feature (*47*). Subsequently, we input the feature and reward into the agent of RL to assess the value and calculate the advantage. This process aids in the policy decision-making for the two drugs, CSF1R_I and IGF1R_I. The policy outputs are then converted into probabilities using the SoftMax function, enabling the selection of the action for the next time step based on these probabilities.

It is important to note that both the reward and advantage should be computed in a discounted form prior to their utilization. Given that the treatment regimen involves a combination of the two drugs, it is necessary to implement two separate policy networks to make decisions regarding the administration of CSF1R_I and IGF1R_I, leading to a two-dimensional action space. Furthermore, during the evaluation phase, we solely need to collect the state from the environment and input it into the two trained policy networks for decision-making, without requiring the value network or the calculation of the reward and advantage. The specific pseudocode of the A3C network training is given in algorithm S2, as well as the corresponding variables and parameters are detailed in table S6.

Algorithm S1. Surrogate model-based A3C network training.

**Algorithm S1.** A3C network training adapted from (*53*)
**Require:** parameters & variables in table S5<br>Initialize counter $t \leftarrow t_{preprocess}$<br>Initialize global shared counter $n_{itera} \leftarrow 0$<br>**while** $n_{itera} < N_{itera}$ **do**<br>reset gradients $d\theta_L \leftarrow 0;\ d\theta_C \leftarrow 0; d\theta_I \leftarrow 0; d\theta_v \leftarrow 0$<br>synchronize with global parameters $\theta_L' \leftarrow \theta_L;\ \theta_C' \leftarrow \theta_C; \theta_I' \leftarrow \theta_I; \theta_v' \leftarrow \theta_v$<br>$t_{start} = t$<br>Obtain state s_t<br>**while** $t - t_{start} < t_{max}$ **do**<br>perform action a_t^C, a_t^I using policy $\pi_C(a_t^C \| s_t; \theta_L', \theta_C')$ and $\pi_I(a_t^I \| s_t; \theta_L', \theta_I')$ respectively<br>receive reward r_t and new state s_{t+1}<br>$t \leftarrow t + 1$<br>$n_{itera} \leftarrow n_{itera} + 1$<br>**end while**<br>$$R = \begin{cases} 0 & \text{for terminal } s_t \\ V(s_t; \theta_L', \theta_v') & \text{for non} - \text{terminal } s_t \end{cases}$$<br>**for** $i \in \{t-1, \cdots, t_{start}\}$ **do**<br>$R \leftarrow r_i + \gamma R$<br>$d\theta_C \leftarrow d\theta_C + \nabla_{\theta_C'} \log \pi_C(a_t^C \| s_t; \theta_L', \theta_C')\,(R - V(s_t; \theta_L', \theta_v'))$<br>$d\theta_I \leftarrow d\theta_I + \nabla_{\theta_I'} \log \pi_I(a_t^I \| s_t; \theta_L', \theta_I')\,(R - V(s_t; \theta_L', \theta_v'))$<br>$d\theta_L \leftarrow d\theta_L + \partial(R - V(s_t; \theta_L', \theta_v'))^2 / \partial\theta_L'$<br>$d\theta_v \leftarrow d\theta_v + \partial(R - V(s_t; \theta_L', \theta_v'))^2 / \partial\theta_v'$<br>**end for**<br>asynchronous update of $\theta_C, \theta_I, \theta_L, \theta_v$ using $d\theta_c, d\theta_I, d\theta_L, d\theta_v$ respectively<br>**end while**

Algorithm S2. Reward function in RL.

**Algorithm S2.** Reward function in RL.
**Require:** parameters & variables in table S6<br>Initialize $r_t \leftarrow base$<br>**if** $p_{death} \geq th_{death}$ **then**<br>$r_t \leftarrow death$<br>**else if** $p_{cure} \geq th_{cure}$ **then**<br>$r_t \leftarrow r_t + cure$<br>**else**<br>**if** $p_{death} \geq 0.5th_{death}$ **then**<br>$r_t \leftarrow r_t - punish * (1 - dose_C)$<br>**else if** $dose_C = 0$ **then**<br>$r_t \leftarrow r_t + holiday$<br>**end if**<br>**if** $p_{survival} \leq 0.9th_{cure}$ **then**<br>$r_t \leftarrow r_t - punish * (1 - dose_I)$<br>**else if** $dose_I = 0$ **then**<br>$r_t \leftarrow r_t + holiday$<br>**end if**<br>$r_t \leftarrow r_t + \lambda * saved_C$<br>**end if**<br>**return** r_t

Fig. S1.

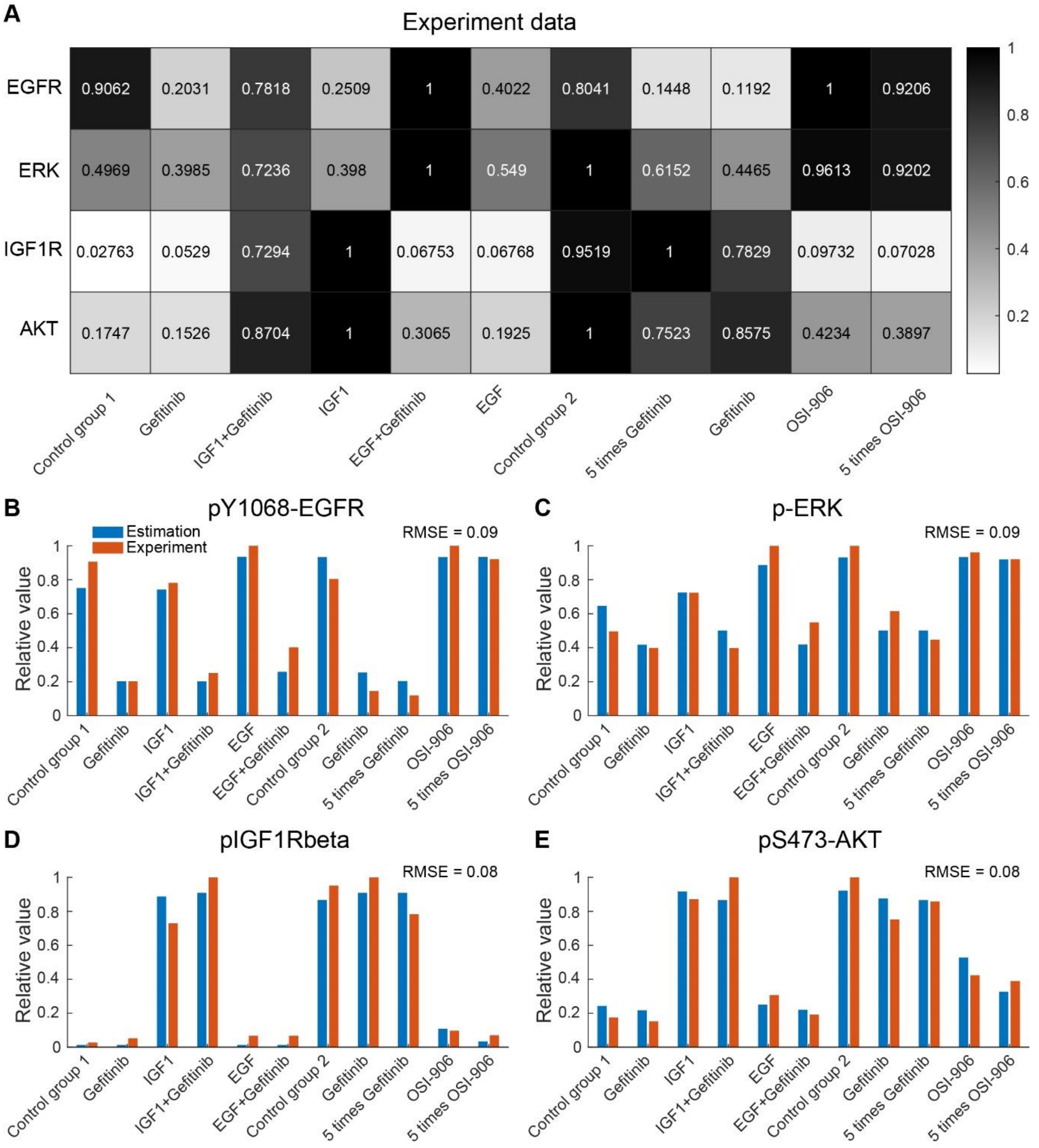

Fig. S1. Estimation of parameters in signaling pathways. (**A**) Quantification of the experimental data from Ma Y, et al. (*51*). (**B-E**) Comparison between the predicted activation levels of EGFR, ERK, IGF1R, and AKT with the corresponding experimental data under different conditions.

Fig. S2.

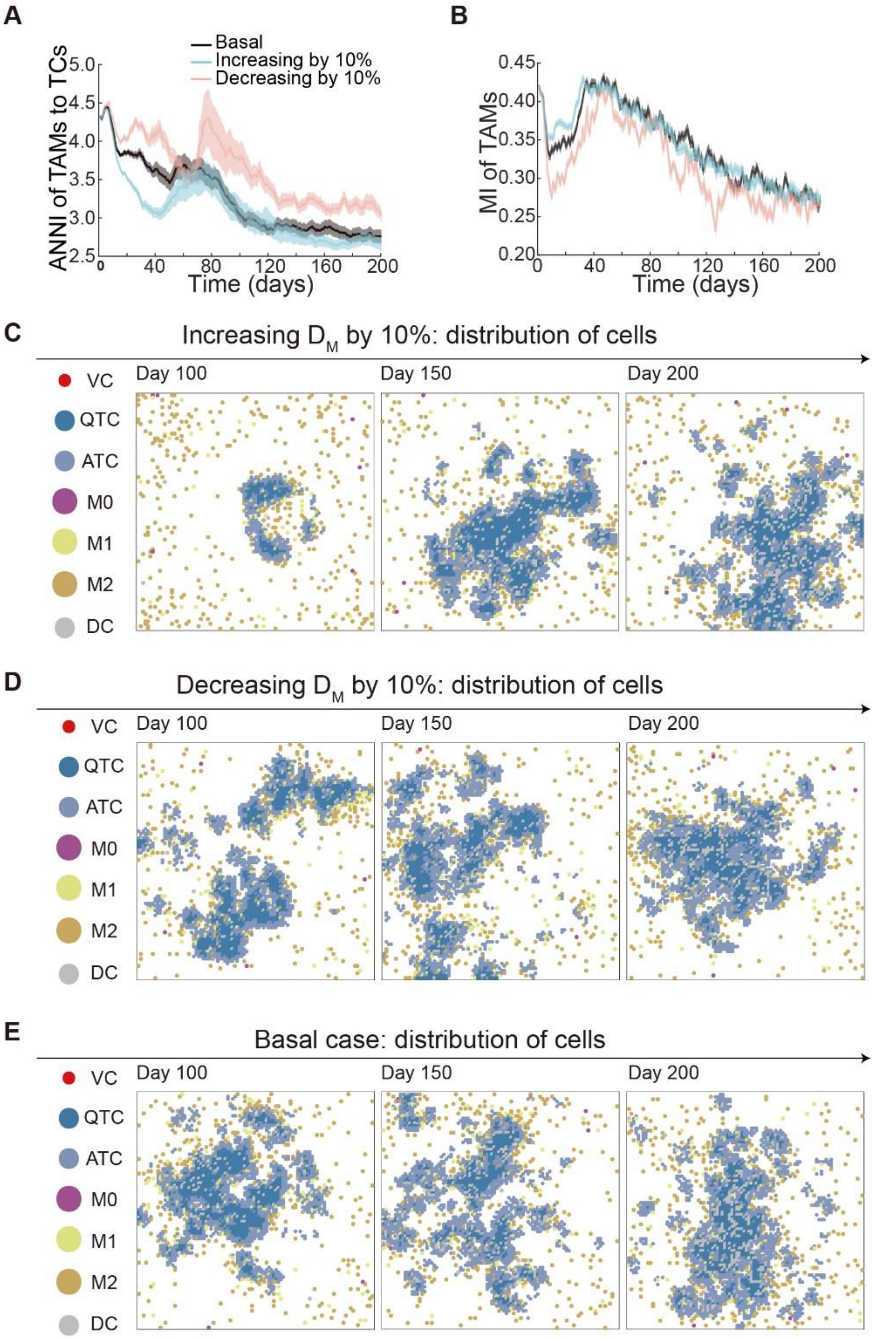

Fig. S2. Supplementary analysis of D_M in continuous-CSF1R_I-treatment case. (**A**) The mean ANNI (with 0.1×SD) of TAMs to tumor cells across the predicted population when D_M changes. (**B**) The mean MI of TAMs (with 0.1×SD) across the predicted population when D_M change. (**C**) Cell distributions for a non-responder to CSF1R_I with D_M increased by 10%. (**D**) Cell distributions for a non-responder to CSF1R_I with D_M decreased by 10%. (**E**) Cell distributions for a non-responder to CSF1R_I with basal D_M.

Fig. S3.

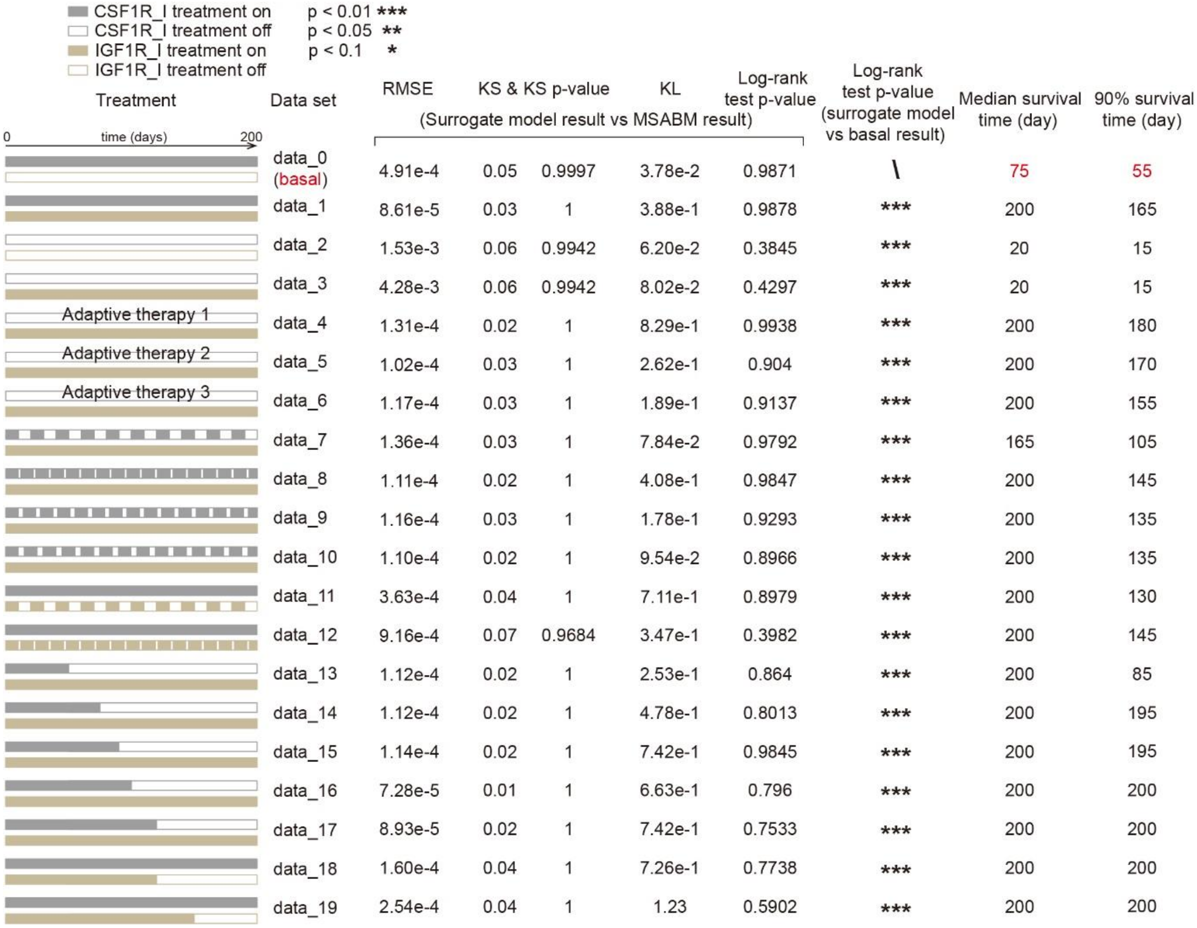

p < 0.01 ***
p < 0.05 **
p < 0.1 *

Data set	RMSE	KS & KS p-value		KL	Log-rank test p-value	Log-rank test p-value (surrogate model vs basal result)	Median survival time (day)	90% survival time (day)
	(Surrogate model result vs MSABM result)							
data_0 (basal)	4.91e-4	0.05	0.9997	3.78e-2	0.9871	\	75	55
data_1	8.61e-5	0.03	1	3.88e-1	0.9878	***	200	165
data_2	1.53e-3	0.06	0.9942	6.20e-2	0.3845	***	20	15
data_3	4.28e-3	0.06	0.9942	8.02e-2	0.4297	***	20	15
data_4	1.31e-4	0.02	1	8.29e-1	0.9938	***	200	180
data_5	1.02e-4	0.03	1	2.62e-1	0.904	***	200	170
data_6	1.17e-4	0.03	1	1.89e-1	0.9137	***	200	155
data_7	1.36e-4	0.03	1	7.84e-2	0.9792	***	165	105
data_8	1.11e-4	0.02	1	4.08e-1	0.9847	***	200	145
data_9	1.16e-4	0.03	1	1.78e-1	0.9293	***	200	135
data_10	1.10e-4	0.02	1	9.54e-2	0.8966	***	200	135
data_11	3.63e-4	0.04	1	7.11e-1	0.8979	***	200	130
data_12	9.16e-4	0.07	0.9684	3.47e-1	0.3982	***	200	145
data_13	1.12e-4	0.02	1	2.53e-1	0.864	***	200	85
data_14	1.12e-4	0.02	1	4.78e-1	0.8013	***	200	195
data_15	1.14e-4	0.02	1	7.42e-1	0.9845	***	200	195
data_16	7.28e-5	0.01	1	6.63e-1	0.796	***	200	200
data_17	8.93e-5	0.02	1	7.42e-1	0.7533	***	200	200
data_18	1.60e-4	0.04	1	7.26e-1	0.7738	***	200	200
data_19	2.54e-4	0.04	1	1.23	0.5902	***	200	200

Fig. S3. In silico treatments for MSABM input and statistical analysis for surrogate model vs MSABM results. The leftmost column lists a total of 20 treatment regimens. Comparing the surrogate model result with the MASBM result yields a low RMSE, a low KS statistic with a KS p-value close to 1, and a relatively low KL value. Additionally, the log-rank test results show no significant. These collectively indicate that the surrogate model can be accurately driven by the output data of MSABM. The rightmost three columns present a comparison between the surrogate model result and the basal case result, with all log-rank tests being significant. This demonstrates that the selection of data constraints exhibits significant differences. From the comparison of median survival time and 90% survival time, it shows that the combined use of CSF1R_I and IGF1R_I significantly outperforms the basal case.

Fig. S4.

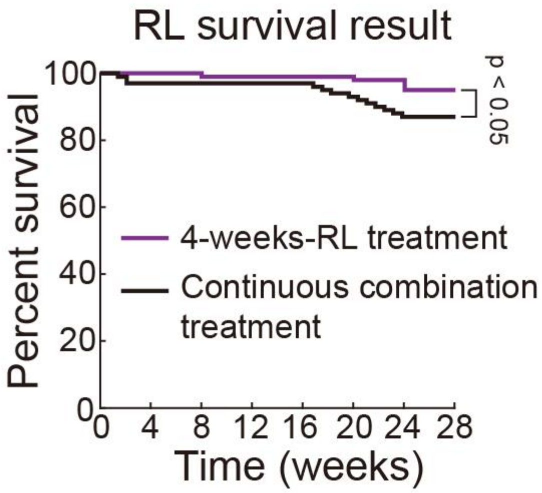

Fig. S4. Survival analysis under the 4-weeks-RL treatment regimen.

Fig. S5.

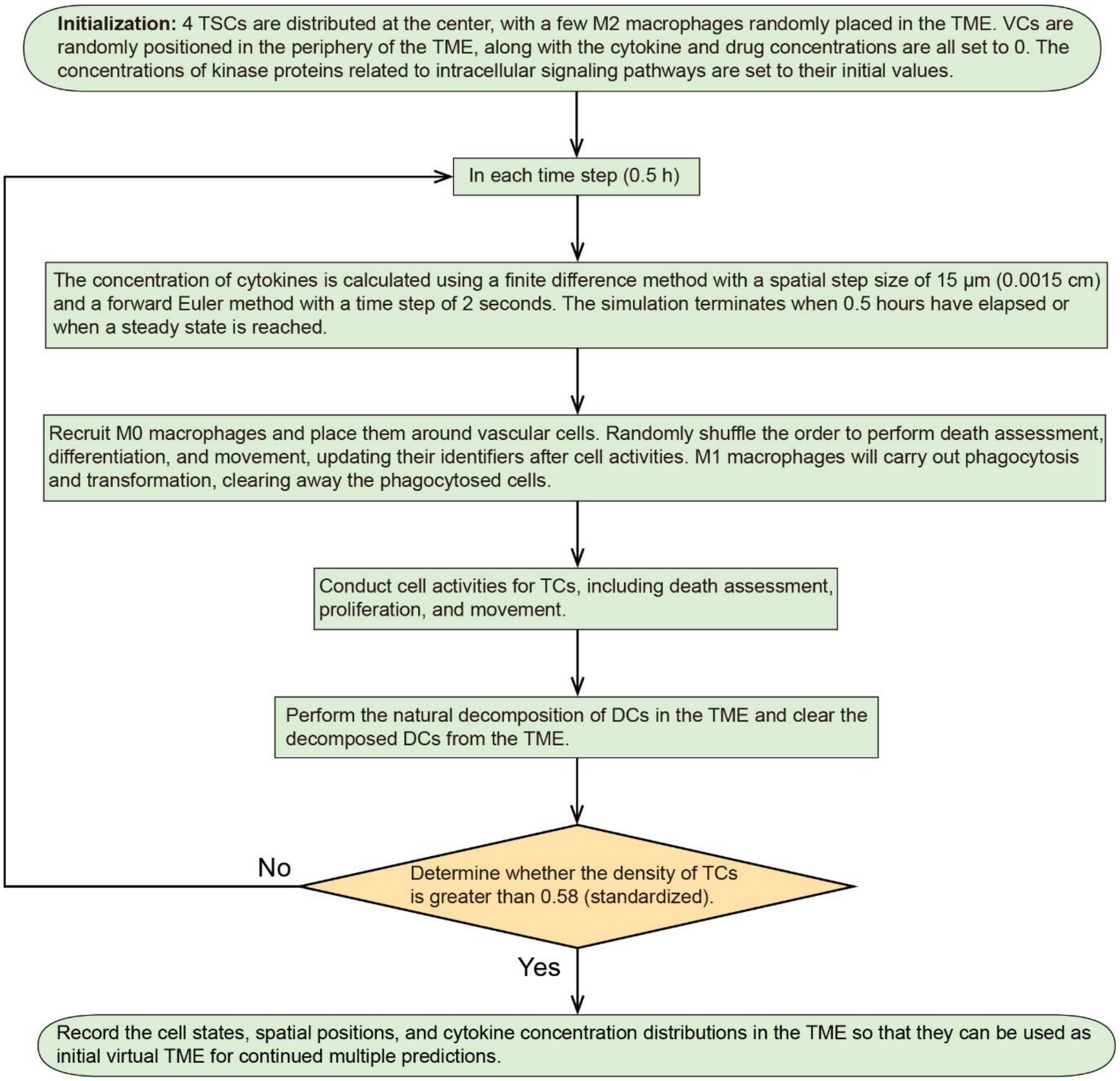

Fig. S5. Flowchart for a single simulation to generate the initial virtual TME for subsequent simulations and predictions.

Fig. S6.

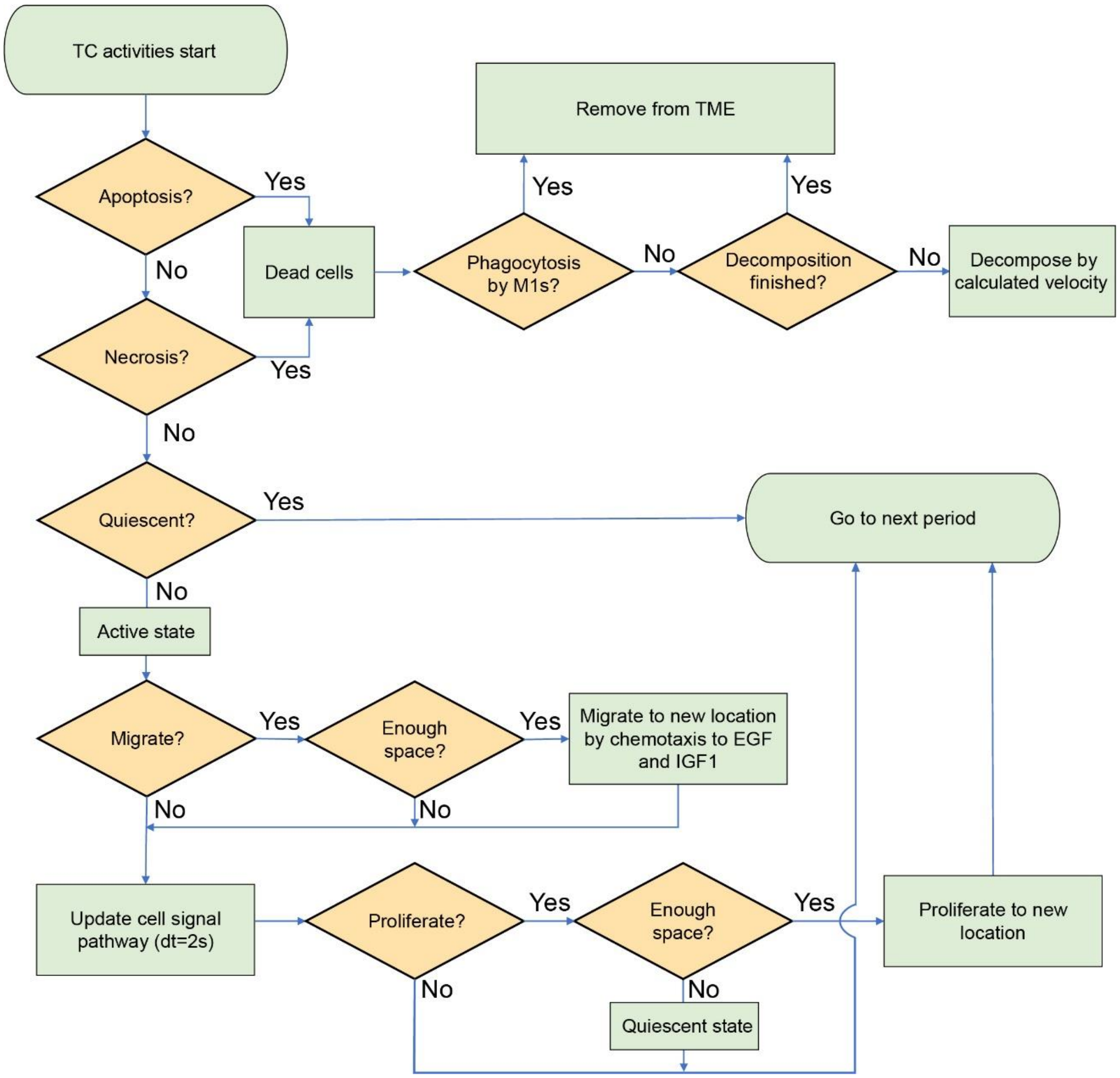

Fig. S6. Flowchart of simulating tumor cell activities.

Fig. S7.

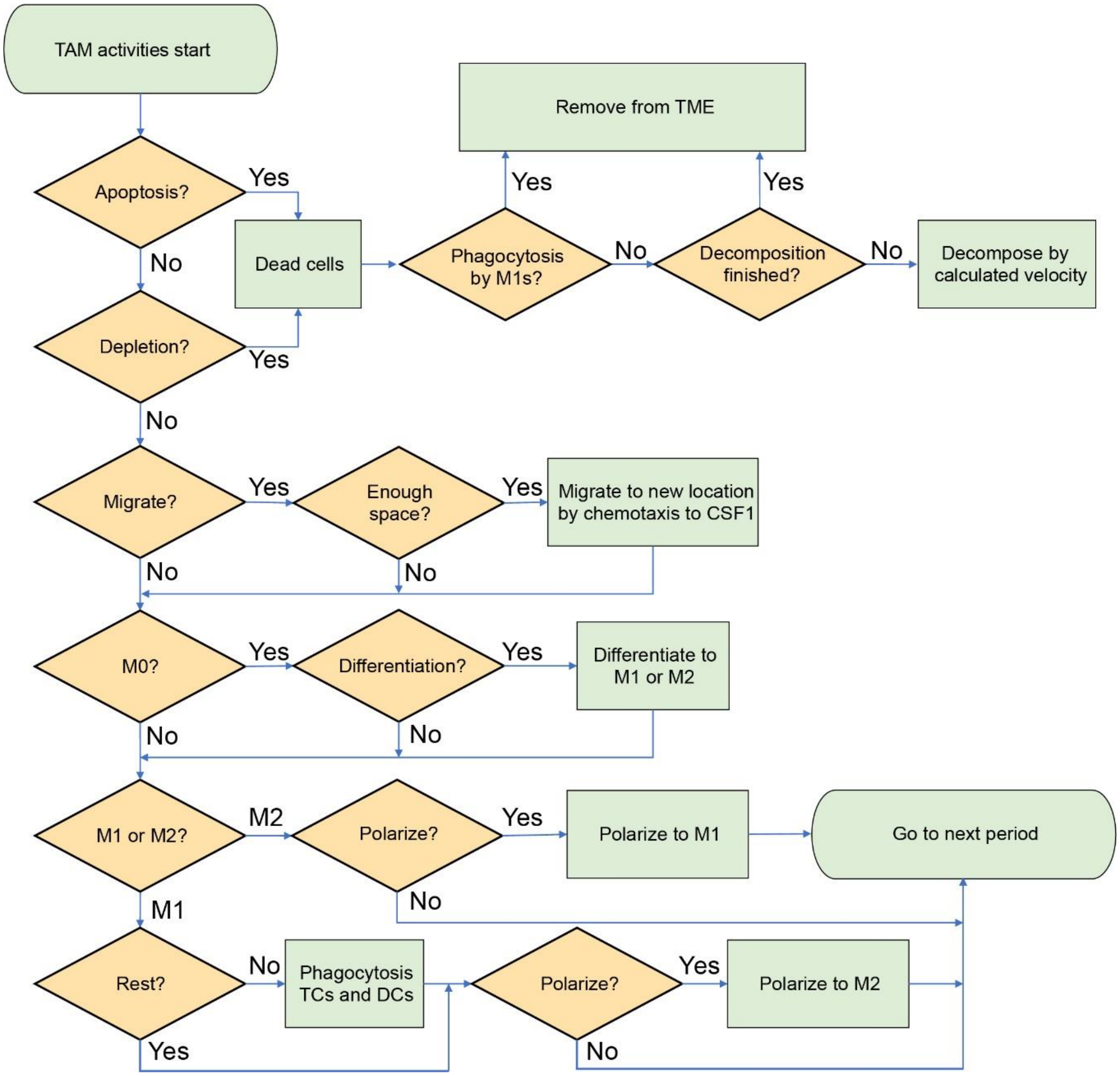

Fig. S7. Flowchart of simulating TAM activities.

Fig. S8.

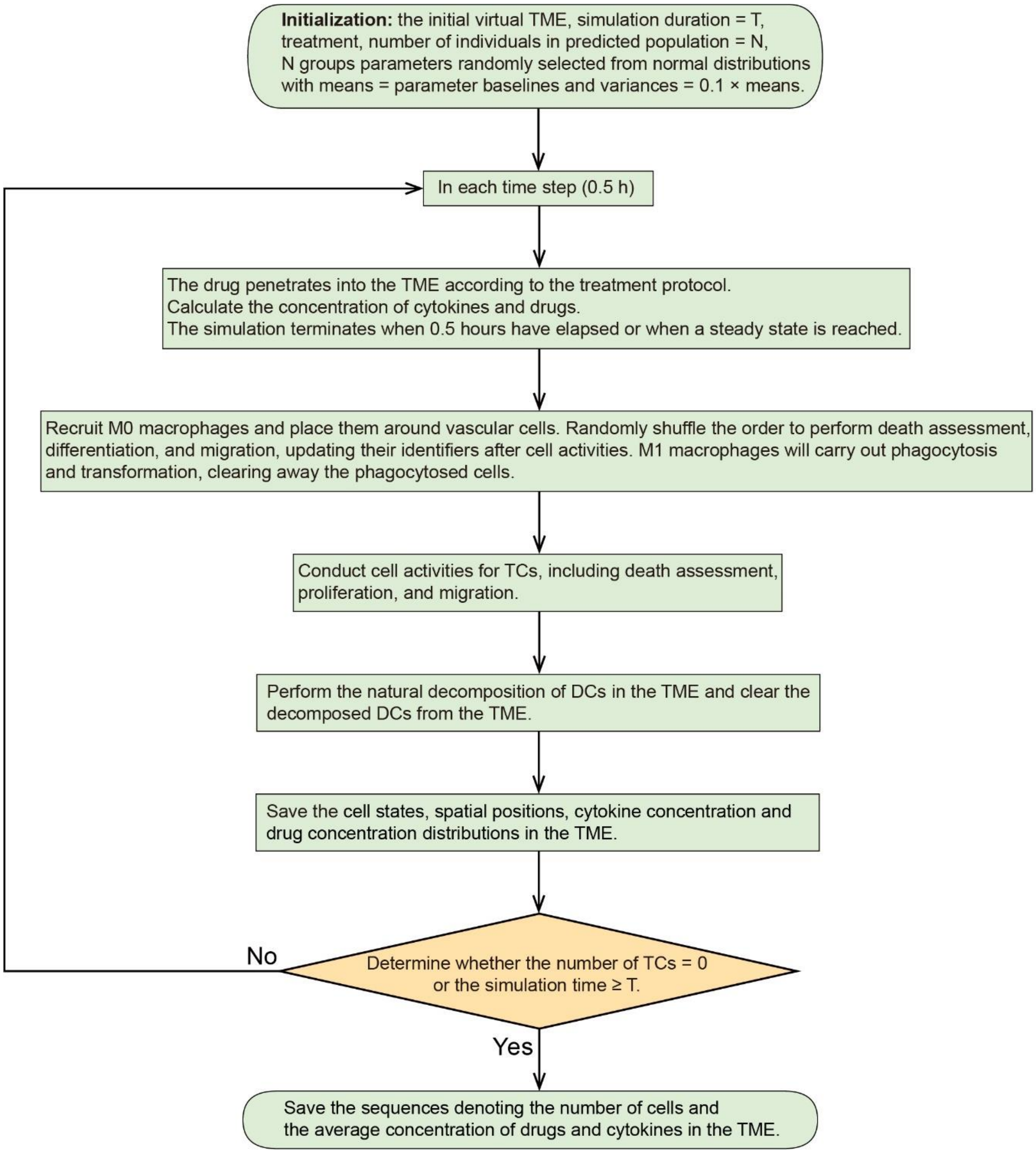

Fig. S8. Flowchart of multiple predictions.

Fig. S9.

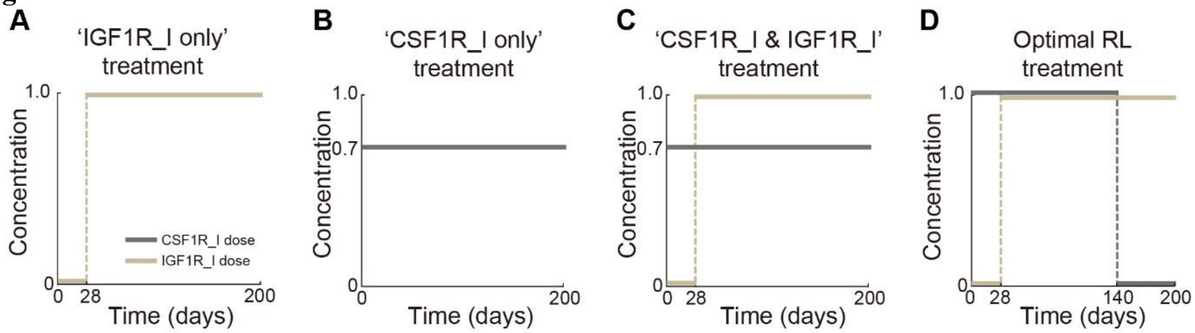

Fig. S9. Treatments used in multiple predictions with TME inputs derived from the ST data.

Fig. S10.

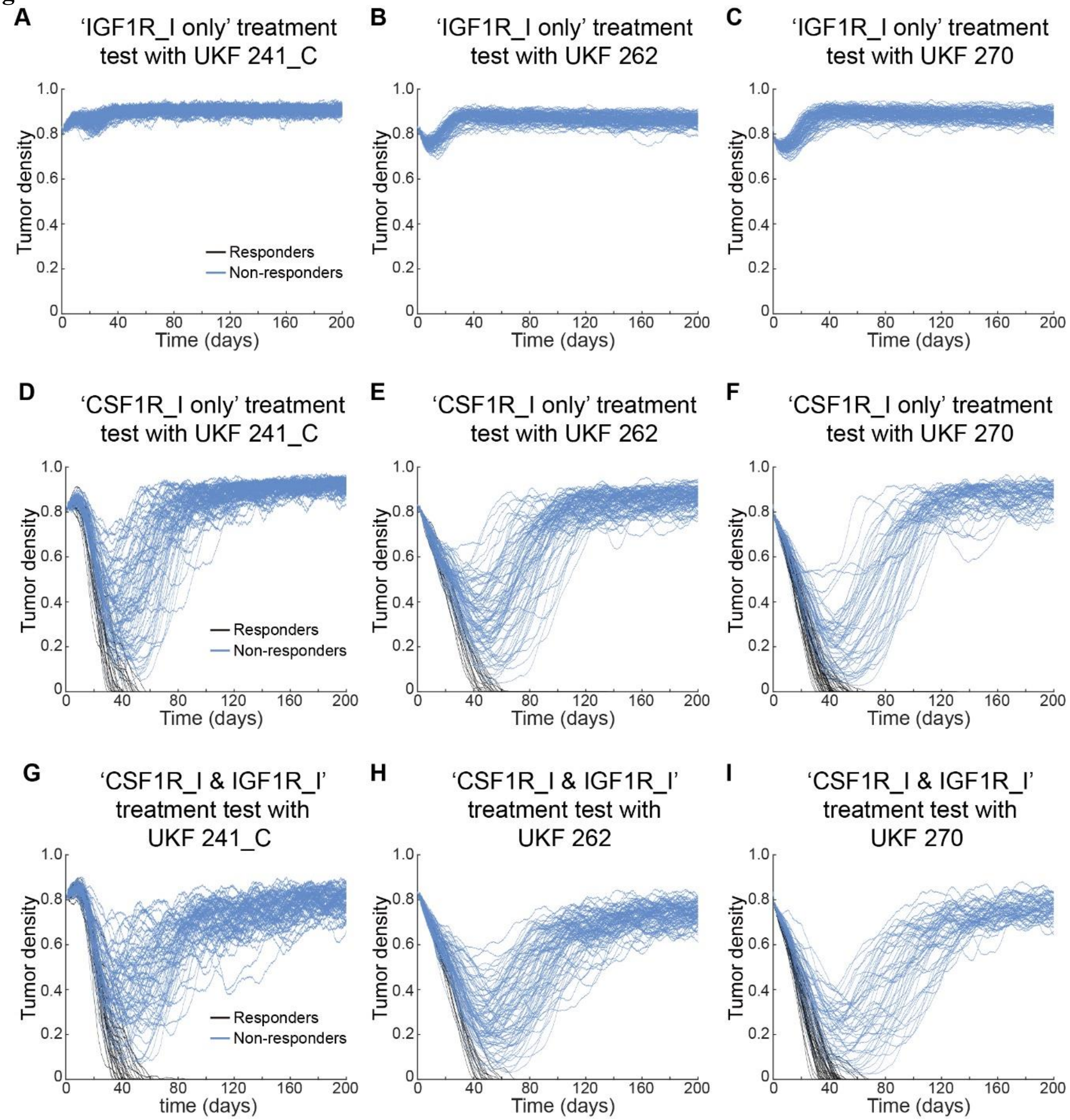

Fig. S10. Results of 'IGF1R_I only', 'CSF1R_I only' and 'CSF1R_I & IGF1R_I' treatment test with ST data-derived TME. (**A-C**) The 'IGF1R_I only' treatment could moderately inhibit tumor cell proliferation but was ineffective in significantly reducing tumor cell density within the TME. (**D-F**) The 'CSF1R_I only' treatment can decrease tumor cell density, but cumulative administration may lead to resistance in some individuals, with high tumor density (> 0.8) following relapse. (**G-I**) The 'CSF1R_I & IGF1R_I' treatment can maintain a lower tumor density (0.6-0.8) in relapsed individuals compared to the 'CSF1R_I only' treatment.

Fig. S11.

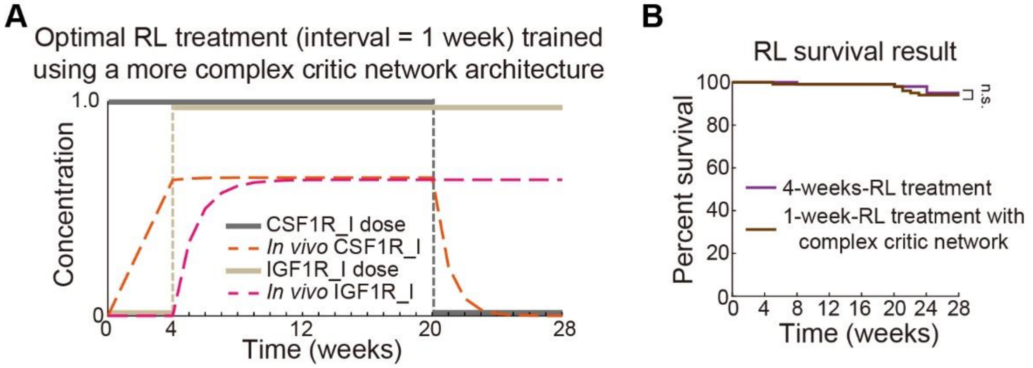

Fig. S11. RL results with a more complex architecture critic network under the 1-week drug recommendation interval. (**A**) The optimal RL treatment under the 1-week drug recommendation interval was obtained by training with a more complex critic network architecture (as shown in table S5). The optimal combination treatment strategy is equivalent to the optimal 4-week-RL treatment after 50,000 global training epochs (1960 core-hours). (**B**) The survival curve under the optimal treatment in (**A**) shows no significant difference compared to that under the optimal 4-weeks-RL treatment.

Fig. S12.

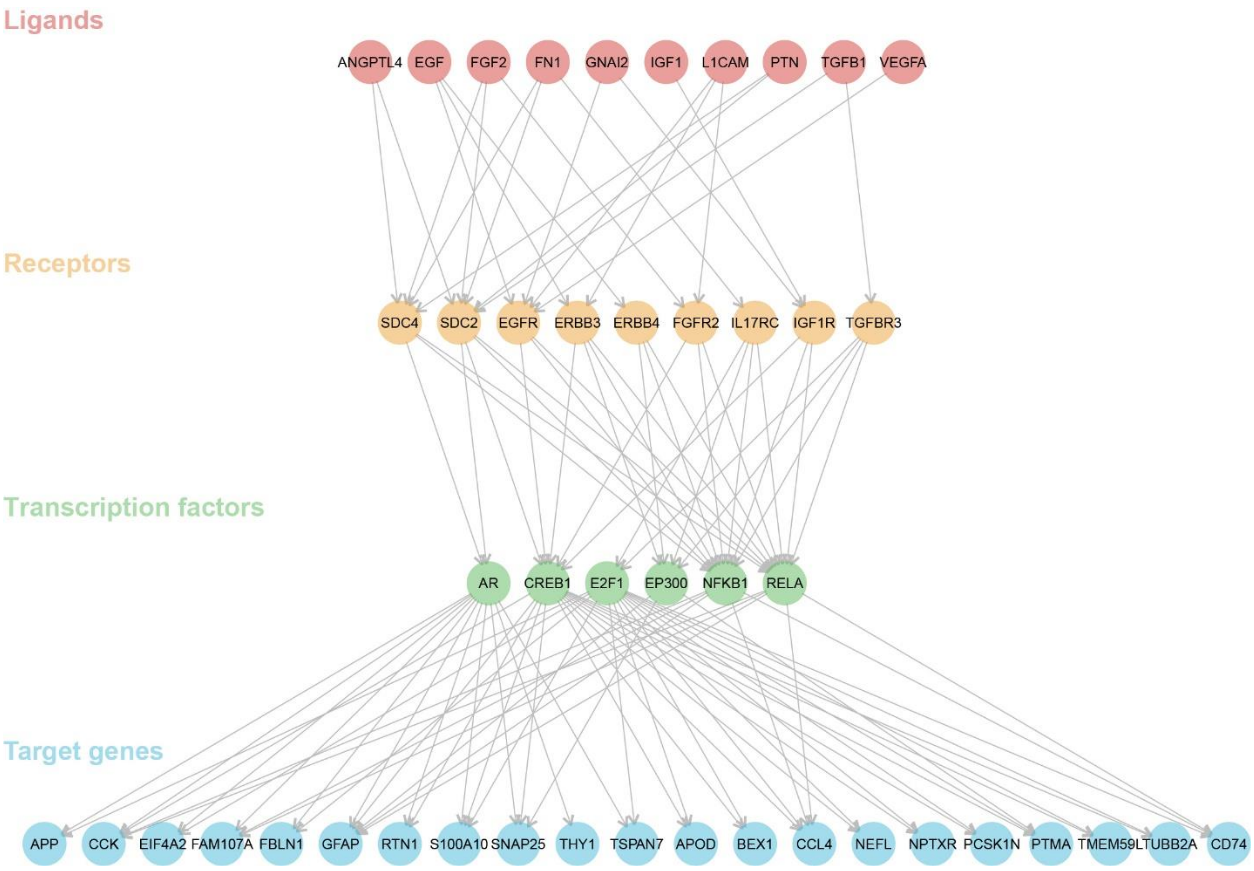

Fig. S12. Macrophage-to-glioma cell signaling network derived from ST data. The network illustrates ligand–receptor–transcription factor–target gene signaling interactions from macrophages to glioma cells, constructed based on stMLnet analysis (*64*) of sample UKF 262 from (*54, 55*). The analysis reveals key signaling pathways central to our MSABM framework, including the EGF–EGFR and IGF1–IGF1R pathways. Downstream transcription factors such as RELA, NFKB1, and CREB1, along with their respective target genes, are also highlighted.

Table S1. Rules for cell agents in MSABM.

Agent	Behavior	Source
Tumor cells	Apoptosis	(*87*)
	Necrosis	(*88*)
	Cell motility	(*89*)
	Chemotaxis towards EGF, IGF1	(*90*)
	Responsiveness to IGF1R_I	(*7*)
	Quiescent state	(*91, 92*)
	Cell division and cell cycle	(*93*)
	CSF1 secretion	(*7, 90*)
Macrophages	Recruitment	(*50*)
	Polarization	(*7, 10, 59*)
	Responsiveness to CSF1R_I	(*7, 10, 90*)
	Cell motility	(*89*)
	Chemotaxis towards CSF1	(*90*)
	Secreting IGF1, EGF	(*7, 48, 49, 90*)
	Apoptosis	(*94*)
	Depletion	(*95*)
	Phagocytosis	(*49, 65, 95*)
Dead cells	Programmed cell removal	(*65*)

Table S2. Parameters in MSABM.

Category	Symbol	Representation	Value	Unit	Reference
Simulation	\	Simulation time interval of MSABM	0.5	hour	(*34*)
Diffusion	dx	Gird size and spatial step in diffusion	0.0015	cm	(*34, 96*)
	dt	Time step in diffusion	2	s	Model specific
Cytokines in eq. (1-2)	\	Diffusion coefficient of cytokines		$cm^2\ s^{-1}$	(*97*)
	S_{CSF1}^{TC}	Secretion rate of CSF1 from tumor cells	2.5×10^{-14}	$s^{-1}\ M^{-1}$	(*98, 99*)
	S_{EGF}^{M2}	Secretion rate of EGF from M2 macrophages	5.0×10^{-15}	$s^{-1}\ M^{-1}$	(*98, 99*)
	S_{IGF1}^{M2}	Secretion rate of IGF1 from M2 macrophages	5.0×10^{-15}	$s^{-1}\ M^{-1}$	(*98, 99*)
	d_{CSF1}	Degradation rate of CSF1	5.0×10^{-4}	s^{-1}	(*98-100*)
	d_{EGF}	Degradation rate of EGF	2.5×10^{-4}	s^{-1}	(*98, 99, 101*)
	d_{IGF1}	Degradation rate of IGF1	2.5×10^{-4}	s^{-1}	(*98, 99, 101*)
	S_A	Cumulative effect coefficient of CSF1R_I on the secretion of IGF1	3.8715×10^{-7}	s^{-1}	(*7, 44*)
	A_0	Basal secretion coefficient of IGF1	0	s^{-1}	(*7, 44*)
	$[CSF1]_{\max}$	Maximum concentration of CSF1 in the TME	4.0×10^{-12}	M	Calibrated
	$[EGF]_{\max}$	Maximum concentration of EGF in the TME	6.0×10^{-13}	M	Calibrated
	$[IGF1]_{\max}$	Maximum concentration of IGF1 in the TME	1.2×10^{-13}	M	Calibrated
Drugs in eq. (3)	D_{drug}	Diffusion coefficient of CSF1R_I and IGF1R_I	2.0×10^{-10}	$cm^2\ s^{-1}$	Updated from (*98, 99, 102*)
	q_{drug}	vascular permeability of CSF1R_I and IGF1R_I	0.8	s^{-1}	Updated from (*43*)
	$u_{CSF1R\_I}^{M}$	TAMs uptake rate of CSF1R_I	2.0×10^{-7}	s^{-1}	Calibrated
	$u_{IGF1R\_I}^{TC}$	Tumor cells uptake rate of IGF1R_I	2.0×10^{-7}	s^{-1}	Calibrated
	η_{drug}	Natural decay rate of CSF1R_I and IGF1R_I	2.0×10^{-7}	s^{-1}	Calibrated
Cells migration	h	Spatial step size in eq. (7) and eq. (10)	0.0015	cm	(*34, 96, 103*)
	k	Time step size in eq. (7) and eq. (10)	112.5~562.5	s	(*103*)

	\	Tumor cells migration interval	24	hours	Adapted from (*34, 103*)
	\	TAMs migration interval	0.5	hour	(*34*)
Tumor cells	D_{TC}	Diffusion coefficient of tumor cells	1.0×10^{-9}	$cm^2 s^{-1}$	(*89, 104*)
	α_{TI} , α_{TE}	Chemotaxis coefficient of IGF1 and EGF for tumor cells	2600	$cm^2 s^{-1} M^{-1}$	Calculated in (*105*)
	p_{TC}	Tumor cell basal proliferation probability	0.01	per cell cycle per cell	Calibrated
	p_D	Tumor cell basal death probability by accident	0.0001	per simulation time interval per cell	Calibrated
	\	Lifespan of tumor cells	8	days	Adapted from (*34, 106-108*)
	\	Mature time of tumor cells	28	hours	(*34, 106-108*)
	\	Division time of tumor cells	28	hours	(*34, 106-108*)
	$N_{TC}^{\max}$	Maximum carrying capacity of tumor cells	2154	cells $(1.5mm)^{-2}$	Calculated from (*98, 99, 109*)
	K_1, K_2	Michaelis constant for the Hill function in eq. (11)	0.5	\	Calibrated
	α_{AKT}	Coefficient associated with AKT to promote tumor growth rate in eq. (11)	44	\	Calibrated
	α_{ERK}	Coefficient associated with ERK to promote tumor growth rate in eq. (11)	14.3	\	Calibrated
Signaling pathways in tumor cells in eq. (12-15)	$[ERK]_{\max}$ $[AKT]_{\max}$, $[IGF1R]_{\max}$, $[EGFR]_{\max}$	Maximum ERK, AKT, IGF1R, EGFR in tumor cells	1.0	\	Normalize d
	$[ERK]_0$	Initial value of ERK in the beginning of simulation	0.49691311 3	\	Normalize d experiment al data from (*51*)
	$[AKT]_0$	Initial value of AKT in the beginning of simulation	0.17467918 6	\	
	$[IGF1R]_0$	Initial value of IGF1R in the beginning of simulation	0.02762651 8	\	
	$[EGFR]_0$	Initial value of EGFR in the beginning of simulation	0.90617109 5	\	
	V_3	A regulatory rate	99.8031372 676745	s^{-1}	Estimated from the

	K_{31}	Michaelis constant for the Hill function of EGF	1.0	\	experimental data (*51*) using genetic algorithm. See fig. S1.
	K_{32}	Michaelis constant for the Hill function of ERK	0.724982430863775	\	
	K_{33}	Michaelis constant for the Hill function of EGFR_I	0.00790513933992095	\	
	d_3	Degradation rate of EGFR	1.56078431465490	s^{-1}	
	V_{41}	A regulatory coefficient for EGFR Hill function	69.5058823535373	\	
	V_{42}	Diffusion coefficient for IGF1R Hill function	33.4117647065726	\	
	V_{43}	A regulatory rate	0.864196307410695	s^{-1}	
	K_{41}	Michaelis constant for the Hill function of EGFR	1.0	\	
	K_{42}	Michaelis constant for the Hill function of IGF1R	0.102633043022255	\	
	K_{43}	Michaelis constant for the Hill function of AKT	0.0214285724285716	\	
	n	Hill coefficient of EGFR Hill function	10	\	
	d_4	Degradation rate of ERK	1.07819925585235	s^{-1}	
	V_5	A regulatory rate	23.8901960789529	s^{-1}	
	K_{51}	Michaelis constant for the Hill function of IGF1	0.0400211238684488	\	
	K_{52}	Michaelis constant for the Hill function of ERK	0.261304749242371	\	
	K_{53}	Michaelis constant for the Hill function of IGF1R_I	0.03	\	
	d_5	Degradation rate of IGF1R	0.753276246188159	s^{-1}	
	V_6	A regulatory rate	16.6823529420039	s^{-1}	
	K_{61}	Michaelis constant for the Hill function of EGFR	0.425704048002236	\	
	K_{62}	Michaelis constant for the Hill function of IGF1R	0.998406371352485	\	
	d_6	Degradation rate of AKT	0.416521770982161	s^{-1}	
Macrophages	D_M	Diffusion coefficient of M0, M1 and M2 macrophages	1.0×10^{-9}	$cm^2 s^{-1}$	(*89, 90*)
	α_M	Chemotaxis coefficient of CSF1 for TAMs	5200	$cm^2 s^{-1} M^{-1}$	(*105*)
	\	Lifespan of TAMs	30	days	(*34, 94*)

	\	Differentiation or polarization interval	3	hours	Calibrated
	$N_M^{\max}$	Maximum carrying capacity of TAMs	450	cells $(1.5\text{mm})^{-2}$	Calculated from (*98, 99, 109*)
	p_{rec}	Basal recruitment probability of M0 macrophages in eq. (16)	3.5×10^{-6}	per simulation time interval per cell	(*34, 97*)
	\	Mature time of TAMs	1	day	Calibrated
	pha	Basal phagocytosis rate of M1 macrophages	1	per simulation time interval per cell	Calibrated
	\	Maximum phagocytosis number of M1 macrophages before having a rest	2	cells	Calibrated
	\	Rest time after reaching the maximum phagocytosis number of TAMs	12	hours	Calibrated
	p_{M01}	Probability of M0 macrophage differentiate into M1	0.05	per differentiation interval per cell	Calibrated
	p_{M02}	Probability of M0 macrophage differentiate into M2	0.75	per differentiation interval per cell	Calibrated
	p_{M21}	Probability of M2 macrophage polarize into M1	0.01944	per polarization interval per cell	Calibrated
	\	Depletion probability of TAMs while using drugs	0~0.01	per simulation time interval per cell	Calibrated
	α_C	Adjustment coefficient of H_C in eq. (17)	0.675	\	Calibrated
	α_I	Adjustment coefficient of H_I in eq. (17)	0.45	\	Calibrated
	K_{M12}	Michaelis constant for the Hill function of CSF1 in eq. (17)	0.02	\	Calibrated
	K_{M12}^d	Michaelis constant for the Hill function of CSF1R_I in eq. (17)	14.3	\	Calibrated

	K_I	Michaelis constant for the Hill function of CSF1R_I accumulation in eq. (17)	5.7	\	Calibrated
Dead cells	v_0	Basal removal rate in eq. (18)	0.5	$hour^{-1}$	Calibrated

Table S3. Parameters in the PINN.

Symbol	Value	Representation
T	200 days	Simulation time in the MSABM
dt_{cyto}	0.5 hour	Simulation time step for cytokines and drugs
dt	5 days	Simulation time step in prior data
dc	0.05 (normalized)	Cell density step in prior data
N_u	20 * 40 = 800	Number of prior data points for data-driven constraints
N_f	200	Number of randomly sampled points in the domain
$train\_data\_num$	20	Number of prior data sets for PINN training
$[E]_{avg}^{0}$	0.5	Initial condition for ODE of normalized average concentration of EGF in the TME
$[C]_{avg}^{0}$	0.3128	Initial condition for ODE of normalized average concentration of CSF1 in the TME
$[CSF1R\_I]_{avg}^{0}$	0	Initial condition for ODE of normalized average concentration of CSF1R_I in the TME
$[I]_{avg}^{0}$	0	Initial condition for ODE of normalized average concentration of IGF1 in the TME
$[IGF1R\_I]_{avg}^{0}$	0	Initial condition for ODE of normalized average concentration of IGF1R_I in the TME
$[TC]_{avg}^{0}$	0.58	Initial condition for ODE of average density of tumor cells in the TME
$[M1]_{avg}^{0}$	35/450	Initial conditions for ODEs corresponding to average density of M1 macrophages in the TME
lb	[0.05, dt_{cyto}]	The lower bound of the density axis and the time axis
ub	[1.0, T]	The upper bound of the density axis and the time axis
FP_layers	[5, 40, 120, 250, 500, 1000, 1000, 600, 300, 150, 1]	Deep neural network setting for approximating Fokker-Planck equations
CC_layers	[4, 40, 40, 40, 40, 40, 40, 40, 40, 1]	Deep neural network setting for approximating ODEs

Table S4. Setups in a standard RL.

Setup	Symbol/definition	Representation/function
Environment	$\mathcal{E}$	The external system interacting with the agent
Agent	/	The learner interacting within the environment
States in the state space	$s',\ s \in \mathcal{S}$	The next state and current state, respectively
State at time t in the state space	$s_t \in \mathcal{S}$	Describing the current situation of the environment at a specific time
Actions in the action space	$a',\ a \in \mathcal{A}$	The next action and current action, respectively
Action at state s_t in the action space	$a_t \in \mathcal{A}(s_t)$	The decision taken by the agent at a specific state
Reward at time t	$r_t(s_t, a_t)$	The immediate feedback signal received by the agent from the environment at a specific time
Discount factor	$\gamma \in (0,\ 1]$	The weight of future rewards, with higher value of which indicates a strong emphasis on future rewards
Return from time t	$R_t = \sum_{k=0}^{\infty} \gamma^k r_{t+k}$	The return (total cumulative reward) obtained from the start of the interaction until its end
Policy function	$\pi(a_t \mid s_t)$	To decide the action under a specific state
State value function	$V(s_t)$	To evaluate the value of a specific state
	$V_\pi(s) = \mathbb{E}[R_t \mid s_t = s,\ \pi]$	The expected return obtained from a specific state under a certain policy
Optimal state value function	$V^*(s) = \max_\pi V_\pi(s)$	The maximum return from a specific state under a certain policy
Optimal policy derived from state value function	$\pi^*(s)$	The policy that can maximize the value of a specific state
Action value function	$Q(s_t,\ a_t)$	To evaluate the value of a specific state-action pair
	$Q_\pi(s,a) = \mathbb{E}[R_t \mid s_t = s, a_t = a, \pi]$	The expected return obtained from a selected action at the specific state under a certain policy
Optimal action value function	$Q^*(s,a) = \max_\pi Q_\pi(s,a)$	The maximum return from a selected action at the specific state under a certain policy
Optimal policy derived from action value function	$\pi^*(s) = \arg\max_a Q^*(s,a)$	The policy that can maximize the value of a specific state-action pair
	$\pi^*(a_t \mid s_t) = \begin{cases} 1, \text{ if } a_t = \arg\max_a Q^*(s_t, a) \\ 0, \text{ otherwise} \end{cases}$	

Learning rate	α	To control the updating rate of parameters

Table S5. Parameters and variables in surrogate model-based A3C network.

Symbol	Value	Representation
dt	1, 2, 3 or 4 weeks	Simulation treatment interval
$T_{predict}$	28 weeks	Maximum simulation time
$T_{preprocess}$	4 weeks	Preprocessing simulation time
$t_{preprocess}$	1	Preprocessing treatment steps
N_{itera}	15000~50000	Iteration number
$t_{\max}$	$T_{predict}/dt$	Maximum treatment steps
α	10^{-4} ((*47*))	Learning rate
β	0.005	Entropy control
γ	0.9999 ((*47*))	Discounted
$\boldsymbol{\theta}_L$, $\boldsymbol{\theta}_L^{'}$	\	Global, local parameters of LSTM
$\boldsymbol{\theta}_C$, $\boldsymbol{\theta}_C^{'}$	\	Global, local parameters of CSF1R_I actor neural network
$\boldsymbol{\theta}_I$, $\boldsymbol{\theta}_I^{'}$	\	Global, local parameters of IGF1R_I actor neural network
$\boldsymbol{\theta}_v$, $\boldsymbol{\theta}_v^{'}$	\	Global, local parameters of critic neural network
$lstm\_hidden\_dim$	4 ((*47*))	Dimension of the LSTM hidden layer
$action\_size$	2	Dimension of the action space (i.e., the number of drugs used in the combination treatment)
\	[$lstm\_hidden\_dim$, 128, 64, 32, 16, 8, 1] ((*47*))	The critic network architecture in Fig. 8A
\	[$lstm\_hidden\_dim$, 64, 1024, 128, 64, 32, 16, 8, 1]	The critic network architecture in fig. S11
\	[$lstm\_hidden\_dim$, 128, 64, 32, 16, 8, $action\_size$] ((*47*))	The actor network architecture in Fig. 8A & fig. S11 (*47*)

Table S6. Parameters and variables in reward function.

Symbol	Value/source	Motivation/representation
$base$	0.1 ((*47*))	Improve high survival probability
$holiday$	0.05 ((*47*))	Encourage drug saving
$punish$	0.1 ((*47*))	Punish no treatment in high death probability case
$death$	-0.1 ((*47*))	Punish high death probability
$cure$	1.0	Rewards of high cure probability
th_{death}	0.2	Death threshold
th_{cure}	0.99	Cure threshold
λ	0.05	Control coefficient
$p_{survival}$	From RL environment	Survival probability
p_{cure}	From RL environment	Cure probability
p_{death}	From RL environment	Death probability
$dose_C$	From RL environment	Last CSF1R_I dose
$dose_I$	From RL environment	Last IGF1R_I dose
$saved_C$	From RL environment	Saved CSF1R_I dose

Caption for movie S1. The virtual TME evolution of an *in silico* individual from an initial small tumor to a high-density tumor. As a supplement to single simulation.

Caption for movie S2. The virtual TME evolution of an *in silico* individual with high-density tumor growth under treatment-naïve condition. As a supplement to Fig. 2A.

Caption for movie S3. The virtual TME evolution of an *in silico* responder with high-density tumor growth under continuous-CSF1R_I-treatment condition. As a supplement to Fig. 3A.

Caption for movie S4. The virtual TME evolution of an *in silico* non-responder with high-density tumor growth under continuous-CSF1R_I-treatment condition. As a supplement to Fig. 3A.

Caption for movie S5. The virtual TME evolution of an *in silico* responder with high-density tumor growth under continuous-CSF1R_I-treatment condition using the non-quiescent model. As a supplement to Fig. 5.

Caption for movie S6. The virtual TME evolution of an *in silico* non-responder with high-density tumor growth under continuous-CSF1R_I-treatment condition using the non-quiescent model. As a supplement to Fig. 5.

REFERENCES AND NOTES

1. F. B. Furnari, T. Fenton, R. M. Bachoo, A. Mukasa, J. M. Stommel, A. Stegh, W. C. Hahn, K. L. Ligon, D. N. Louis, C. Brennan, L. Chin, R. A. DePinho, W. K. Cavenee, Malignant astrocytic glioma: Genetics, biology, and paths to treatment. *Genes Dev.* **21**, 2683–2710 (2007).

2. D. Hambardzumyan, D. H. Gutmann, H. Kettenmann, The role of microglia and macrophages in glioma maintenance and progression. *Nat. Neurosci.* **19**, 20–27 (2016).

3. R. Stupp, W. P. Mason, M. J. van den Bent, M. Weller, B. Fisher, M. J. Taphoorn, K. Belanger, A. A. Brandes, C. Marosi, U. Bogdahn, J. Curschmann, R. C. Janzer, S. K. Ludwin, T. Gorlia, A. Allgeier, D. Lacombe, J. G. Cairncross, E. Eisenhauer, R. O. Mirimanoff, Radiotherapy plus concomitant and adjuvant temozolomide for glioblastoma. *N. Engl. J. Med.* **352**, 987–996 (2005).

4. Y. Komohara, K. Ohnishi, J. Kuratsu, M. Takeya, Possible involvement of the M2 anti-inflammatory macrophage phenotype in growth of human gliomas. *J. Pathol.* **216**, 15–24 (2008).

5. F. Tang, Y. Wang, Y. Zeng, A. Xiao, A. Tong, J. Xu, Tumor-associated macrophage-related strategies for glioma immunotherapy. *npj Precis. Oncol.* **7**, 78 (2023).

6. A. J. Boutilier, S. F. Elsawa, Macrophage polarization states in the tumor microenvironment. *Int. J. Mol. Sci.* **22**, 5996 (2021).

7. D. F. Quail, R. L. Bowman, L. Akkari, M. L. Quick, A. J. Schuhmacher, J. T. Huse, E. C. Holland, J. C. Sutton, J. A. Joyce, The tumor microenvironment underlies acquired resistance to CSF-1R inhibition in gliomas. *Science* **352**, aad3018 (2016).

8. J. Wyckoff, W. Wang, E. Y. Lin, Y. Wang, F. Pixley, E. R. Stanley, T. Graf, J. W. Pollard, J. Segall, J. Condeelis, A paracrine loop between tumor cells and macrophages is required for tumor cell migration in mammary tumors. *Cancer Res.* **64**, 7022–7029 (2004).

9. J. Condeelis, J. W. Pollard, Macrophages: Obligate partners for tumor cell migration, invasion, and metastasis. *Cell* **124**, 263–266 (2006).

10. S. M. Pyonteck, L. Akkari, A. J. Schuhmacher, R. L. Bowman, L. Sevenich, D. F. Quail, O. C. Olson, M. L. Quick, J. T. Huse, V. Teijeiro, M. Setty, C. S. Leslie, Y. Oei, A. Pedraza, J. Zhang, C. W. Brennan, J. C. Sutton, E. C. Holland, D. Daniel, J. A. Joyce, CSF-1R inhibition alters macrophage polarization and blocks glioma progression. *Nat. Med.* **19**, 1264–1272 (2013).

11. P. M. Altrock, L. L. Liu, F. Michor, The mathematics of cancer: Integrating quantitative models. *Nat. Rev. Cancer* **15**, 730–745 (2015).

12. J. H. A. Creemers, A. Ankan, K. C. B. Roes, G. Schröder, N. Mehra, C. G. Figdor, I. J. M. de Vries, J. Textor, In silico cancer immunotherapy trials uncover the consequences of therapy-specific response patterns for clinical trial design and outcome. *Nat. Commun.* **14**, 2348 (2023).

13. W. Valega-Mackenzie, M. Rodriguez Messan, O. N. Yogurtcu, U. Nukala, Z. E. Sauna, H. Yang, Dose optimization of an adjuvanted peptide-based personalized neoantigen melanoma vaccine. *PLoS Comput. Biol.* **20**, e1011247 (2024).

14. J. M. Greene, J. L. Gevertz, E. D. Sontag, Mathematical approach to differentiate spontaneous and induced evolution to drug resistance during cancer treatment. *JCO Clin. Cancer Inform.* **3**, 1–20 (2019).

15. R. A. Gatenby, A. S. Silva, R. J. Gillies, B. R. Frieden, Adaptive therapy. *Cancer Res.* **69**, 4894–4903 (2009).

16. R. Liu, S. Wang, X. Tan, X. Zou, Identifying optimal adaptive therapeutic schedules for prostate cancer through combining mathematical modeling and dynamic optimization. *Appl. Math. Model.* **107**, 688–700 (2022).

17. M. A. R. Strobl, J. West, Y. Viossat, M. Damaghi, M. Robertson-Tessi, J. S. Brown, R. A. Gatenby, P. K. Maini, A. R. A. Anderson, Turnover modulates the need for a cost of resistance in adaptive therapy. *Cancer Res.* **81**, 1135–1147 (2021).

18. S. Tang, S. Li, B. Tang, X. Wang, Y. Xiao, R. A. Cheke, Hormetic and synergistic effects of cancer treatments revealed by modelling combinations of radio - or chemotherapy with immunotherapy. *BMC Cancer* **23**, 1040 (2023).

19. C. D. Arvanitis, V. Askoxylakis, Y. Guo, M. Datta, J. Kloepper, G. B. Ferraro, M. O. Bernabeu, D. Fukumura, N. McDannold, R. K. Jain, Mechanisms of enhanced drug delivery in brain metastases with focused ultrasound-induced blood-tumor barrier disruption. *Proc. Natl. Acad. Sci. U.S.A.* **115**, E8717–E8726 (2018).

20. X. Sun, J. Bao, Y. Shao, Mathematical modeling of therapy-induced cancer drug resistance: Connecting cancer mechanisms to population survival rates. *Sci Rep* **6**, 22498 (2016).

21. B. Waclaw, I. Bozic, M. E. Pittman, R. H. Hruban, B. Vogelstein, M. A. Nowak, A spatial model predicts that dispersal and cell turnover limit intratumour heterogeneity. *Nature* **525**, 261–264 (2015).

22. X. Lai, A. Stiff, M. Duggan, R. Wesolowski, W. E. Carson III, A. Friedman, Modeling combination therapy for breast cancer with BET and immune checkpoint inhibitors. *Proc. Natl. Acad. Sci. U.S.A.* **115**, 5534–5539 (2018).

23. X. Zheng, G. Y. Koh, T. Jackson, A continuous model of angiogenesis: Initiation, extension, and maturation of new blood vessels modulated by vascular endothelial growth factor, angiopoietins, platelet-derived growth factor-B, and pericytes. *Discrete Contin. Dyn. Syst. - B.* **18**, 1109–1154 (2013).

24. G. Albano, V. Giorno, P. Roman-Roman, S. Roman-Roman, F. Torres-Ruiz, Estimating and determining the effect of a therapy on tumor dynamics by means of a modified Gompertz diffusion process. *J. Theor. Biol.* **364**, 206–219 (2015).

25. L. Chen, J. Yang, Y. Tan, Z. Liu, R. A. Cheke, Threshold dynamics of a stochastic model of intermittent androgen deprivation therapy for prostate cancer. *Commun. Nonlinear Sci. Numer.* **100**, 105856 (2021).

26. A. C. Sfakianakis, A. J. Mark, A hybrid multiscal model for cancer invasion of the extracellular matrix. *Multiscale Model. Simul.* **18**, 824–850 (2020).

27. K. Zhang, J. Zhu, D. Kong, Z. Zhang, Modeling single cell trajectory using forward-backward stochastic differential equations. *PLoS Comput Biol* **20**, e1012015 (2024).

28. I. Bozic, T. Antal, H. Ohtsuki, H. Carter, D. Kim, S. Chen, R. Karchin, K. W. Kinzler, B. Vogelstein, M. A. Nowak, Accumulation of driver and passenger mutations during tumor progression. *Proc. Natl. Acad. Sci. U.S.A.* **107**, 18545–18550 (2010).

29. T. Arne, S. Reiner, B. Benedikt, Cancer initiation with epistatic interactions between driver and passenger mutations. *J. Theor. Biol.* **358**, 52–60 (2014).

30. K. Hinohara, H. J. Wu, S. Vigneau, T. O. Mcdonald, K. J. Igarashi, K. N. Yamamoto, T. Madsen, A. Fassl, S. B. Egri, M. Papanastasiou, KDM5 histone demethylase activity links cellular transcriptomic heterogeneity to therapeutic resistance. *Cancer Cell* **35**, 330–332 (2018).

31. X. Sun, J. Zhang, Q. Zhao, X. Chen, W. Zhu, G. Yan, T. Zhou, Stochastic modeling suggests that noise reduces differentiation efficiency by inducing a heterogeneous drug response in glioma differentiation therapy. *BMC Syst. Biol.* **10**, 73 (2016).

32. P. G. Jayathilake, P. Victori, C. E. Pavillet, C. H. Lee, D. Voukantsis, A. Miar, A. Arora, A. L. Harris, K. J. Morten, F. M. Buffa, Metabolic symbiosis between oxygenated and hypoxic tumour cells: An agent-based modelling study. *PLoS Comput. Biol.* **20**, e1011944 (2024).

33. P. Van Liedekerke, M. M. Palm, N. Jagiella, D. Drasdo, Simulating tissue mechanics with agent-based models: Concepts, perspectives and some novel results. *Comput. Part. Mech.* **2**, 401–444 (2015).

34. C. G. Cess, S. D. Finley, Multi-scale modeling of macrophage-T cell interactions within the tumor microenvironment. *PLoS Comput. Biol.* **16**, e1008519 (2020).

35. X. Sun, L. Zhang, H. Tan, J. Bao, C. Strouthos, X. Zhou, Multi-scale agent-based brain cancer modeling and prediction of TKI treatment response: Incorporating EGFR signaling pathway and angiogenesis. *BMC Bioinformatics* **13**, 218 (2012).

36. M. M. Olsen, H. T. Siegelmann, Multiscale agent-based model of tumor angiogenesis. *Procedia Comput. Sci.* **18**, 1016–1025 (2013).

37. E. Lima, D. Faghihi, R. Philley, J. Yang, J. Virostko, C. M. Phillips, T. E. Yankeelov, Bayesian calibration of a stochastic, multiscale agent-based model for predicting in vitro tumor growth. *PLoS Comput. Biol.* **17**, e1008845 (2021).

38. J. A. Gallaher, S. C. Massey, A. Hawkins-Daarud, S. S. Noticewala, R. C. Rockne, S. K. Johnston, L. Gonzalez-Cuyar, J. Juliano, O. Gil, K. R. Swanson, P. Canoll, A. R. A. Anderson, From cells to tissue: How cell scale heterogeneity impacts glioblastoma growth and treatment response. *PLoS Comput. Biol.* **16**, e1007672 (2020).

39. A. Randles, H. G. Wirsching, J. A. Dean, Y. K. Cheng, S. Emerson, S. S. Pattwell, E. C. Holland, F. Michor, Computational modelling of perivascular-niche dynamics for the optimization of treatment schedules for glioblastoma. *Nat. Biomed. Eng.* **5**, 346–359 (2021).

40. Z. Zhang, L. Liu, C. Ma, X. Cui, R. H. W. Lam, W. Chen, An in silico glioblastoma microenvironment model dissects the immunological mechanisms of resistance to PD-1 checkpoint blockade immunotherapy. *Small Methods* **5**, 2100197 (2021).

41. H. Yang, H. Lin, X. Sun, Multiscale modeling of drug resistance in glioblastoma with gene mutations and angiogenesis. *Comput. Struct. Biotechnol. J.* **21**, 5285–5295 (2023).

42. S. Stein, R. Zhao, H. Haeno, I. Vivanco, F. Michor, Mathematical modeling identifies optimum lapatinib dosing schedules for the treatment of glioblastoma patients. *PLoS Comput. Biol.* **14**, e1005924 (2018).

43. W. Liang, Y. Zheng, J. Zhang, X. Sun, Multiscale modeling reveals angiogenesis-induced drug resistance in brain tumors and predicts a synergistic drug combination targeting EGFR and VEGFR pathways. *BMC Bioinformatics* **20**, 203 (2019).

44. Y. Zheng, J. Bao, Q. Zhao, T. Zhou, X. Sun, A spatio-temporal model of macrophage-mediated drug resistance in glioma immunotherapy. *Mol. Cancer Ther.* **17**, 814–824 (2018).

45. T. O. McDonald, Y.-C. Cheng, C. Graser, P. B. Nicol, D. Temko, F. Michor, Computational approaches to modelling and optimizing cancer treatment. *Nat. Rev. Bioeng.* **1**, 695–711 (2023).

46. Y. Lu, Q. Chu, Z. Li, M. Wang, R. Gatenby, Q. Zhang, Deep reinforcement learning identifies personalized intermittent androgen deprivation therapy for prostate cancer. *Brief Bioinform.* **25**, bbae071 (2024).

47. K. Gallagher, M. A. R. Strobl, D. S. Park, F. C. Spoendlin, R. A. Gatenby, P. K. Maini, A. R. A. Anderson, Mathematical model-driven deep learning enables personalized adaptive therapy. *Cancer Res.* **84**, 1929–1941 (2024).

48. F. O. Martinez, S. Gordon, M. Locati, A. Mantovani, Transcriptional profiling of the human monocyte-to-macrophage differentiation and polarization: New molecules and patterns of gene expression. *J. Immunol.* **177**, 7303–7311 (2006).

49. A. Mantovani, S. Sozzani, M. Locati, P. Allavena, A. Sica, Macrophage polarization: Tumor-associated macrophages as a paradigm for polarized M2 mononuclear phagocytes. *Trends Immunol.* **23**, 549–555 (2002).

50. A. Mantovani, P. Allavena, S. Sozzani, A. Vecchi, M. Locati, A. Sica, Chemokines in the recruitment and shaping of the leukocyte infiltrate of tumors. *Semin. Cancer Biol.* **14**, 155–160 (2004).

51. Y. Ma, N. Tang, R. C. Thompson, B. C. Mobley, S. W. Clark, J. N. Sarkaria, J. Wang, InsR/IGF1R pathway mediates resistance to EGFR inhibitors in glioblastoma. *Clin. Cancer Res.* **22**, 1767–1776 (2016).

52. P. Baldominos, A. Barbera-Mourelle, O. Barreiro, Y. Huang, A. Wight, J. W. Cho, X. Zhao, G. Estivill, I. Adam, X. Sanchez, S. McCarthy, J. Schaller, Z. Khan, A. Ruzo, R. Pastorello, E. T. Richardson, D. Dillon, P. Montero-Llopis, R. Barroso-Sousa, J. Forman, S. A. Shukla,

S. M. Tolaney, E. A. Mittendorf, U. H. von Andrian, K. W. Wucherpfennig, M. Hemberg, J. Agudo, Quiescent cancer cells resist T cell attack by forming an immunosuppressive niche. *Cell* **185**, 1694–1708.e19 (2022).

53. V. Mnih, A. P. Badia, M. Mirza, A. Graves, T. Harley, T. P. Lillicrap, D. Silver, K. Kavukcuoglu, paper presented at the 33rd International Conference on International Conference on Machine Learning, New York, NY, 11 June 2016.

54. V. M. Ravi, P. Will, J. Kueckelhaus, N. Sun, K. Joseph, H. Salié, L. Vollmer, U. Kuliesiute, J. von Ehr, J. K. Benotmane, N. Neidert, M. Follo, F. Scherer, J. M. Goeldner, S. P. Behringer, P. Franco, M. Khiat, J. Zhang, U. G. Hofmann, C. Fung, F. L. Ricklefs, K. Lamszus, M. Boerries, M. Ku, J. Beck, R. Sankowski, M. Schwabenland, M. Prinz, U. Schüller, S. Killmer, B. Bengsch, A. K. Walch, D. Delev, O. Schnell, D. H. Heiland, Spatially resolved multi-omics deciphers bidirectional tumor-host interdependence in glioblastoma. *Cancer Cell* **40**, 639–655.e13 (2022).

55. J. Kueckelhaus, S. Frerich, J. Kada-Benotmane, C. Koupourtidou, J. Ninkovic, M. Dichgans, J. Beck, O. Schnell, D. H. Heiland, Inferring histology-associated gene expression gradients in spatial transcriptomic studies. *Nat. Commun.* **15**, 7280 (2024).

56. R. Dong, G.-C. Yuan, SpatialDWLS: Accurate deconvolution of spatial transcriptomic data. *Genome Biol.* **22**, 145 (2021).

57. S. K. Longo, M. G. Guo, A. L. Ji, P. A. Khavari, Integrating single-cell and spatial transcriptomics to elucidate intercellular tissue dynamics. *Nat. Rev. Genet.* **22**, 627–644 (2021).

58. S. D. Jayasingam, M. Citartan, T. H. Thang, A. A. Mat Zin, K. C. Ang, E. S. Ch'ng, Evaluating the polarization of tumor-associated macrophages into M1 and M2 phenotypes in human cancer tissue: Technicalities and challenges in routine clinical practice. *Frontiers in oncology* **9**, 1512 (2019).

59. S. J. Coniglio, E. Eugenin, K. Dobrenis, E. R. Stanley, B. L. West, M. H. Symons, J. E. Segall, Microglial stimulation of glioblastoma invasion involves epidermal growth factor

receptor (EGFR) and colony stimulating factor 1 receptor (CSF-1R) signaling. *Mol. Med.* **18**, 519–527 (2012).

60. D. M. Mosser, J. P. Edwards, Exploring the full spectrum of macrophage activation. *Nat. Rev. Immunol.* **8**, 958–969 (2008).

61. S. V. Kushchayev, T. Sankar, L. L. Eggink, Y. S. Kushchayeva, P. C. Wiener, J. K. Hoober, J. Eschbacher, R. Liu, F. D. Shi, M. G. Abdelwahab, A. C. Scheck, M. C. Preul, Monocyte galactose/N-acetylgalactosamine-specific C-type lectin receptor stimulant immunotherapy of an experimental glioma. Part II: Combination with external radiation improves survival. *Cancer Manag. Res.* **4**, 325–334 (2012).

62. S. V. Kushchayev, T. Sankar, L. L. Eggink, Y. S. Kushchayeva, P. C. Wiener, J. K. Hoober, J. Eschbacher, R. Liu, F. D. Shi, M. G. Abdelwahab, A. C. Scheck, M. C. Preul, Monocyte galactose/N-acetylgalactosamine-specific C-type lectin receptor stimulant immunotherapy of an experimental glioma. Part 1: Stimulatory effects on blood monocytes and monocyte-derived cells of the brain. *Cancer Manag. Res.* **4**, 309–323 (2012).

63. D. F. Quail, J. A. Joyce, The microenvironmental landscape of brain tumors. *Cancer Cell* **31**, 326–341 (2017).

64. L. Yan, J. Cheng, Q. Nie, X. Sun, Dissecting multilayer cell-cell communications with signaling feedback loops from spatial transcriptomics data. *Genome Res.* **35**, 1400–1414 (2025).

65. D. J. Kloosterman, L. Akkari, Macrophages at the interface of the co-evolving cancer ecosystem. *Cell* **186**, 1627–1651 (2023).

66. E. L. Kaplan, P. Meier, "Nonparametric estimation from incomplete observations" in *Breakthroughs in Statistics*, S. Kotz, N. L. Johnson, Eds. (Springer, 1992), pp. 319–337.

67. R. Peto, M. C. Pike, P. Armitage, N. E. Breslow, D. R. Cox, S. V. Howard, N. Mantel, K. Mcpherson, J. Peto, P. G. Smith, Design and analysis of randomized clinical trials requiring

prolonged observation of each patient. II. Analysis and examples. *Br. J. Cancer* **35**, 1–39 (1977).

68. P. C. Bressloff, “Random walks and Brownian motion” in *Stochastic Processes in Cell Biology* (Interdisciplinary Applied Mathematics, Springer, ed. 2, 2021), pp. 57–64.

69. Z. Chen, Y. Liu, H. Sun, Physics-informed learning of governing equations from scarce data. *Nat. Commun.* **12**, 6136 (2021).

70. M. Raissi, P. Perdikaris, G. E. Ksrniadakis, Physics-informed neural networks: A deep learning framework for solving forward and inverse problems involving nonlinear partial differential equations. *J. Comput. Phys.* **378**, 686–707 (2019).

71. R. S. Sutton, A. G. Barto, *Reinforcement Learning:An Introduction* (MIT Press, 1998).

72. Y. Gao, Y. Liu, H. Zhang, Z. Li, Y. Zhu, H. Lin, M. Yang, paper presented at the 28th ACM Joint Meeting on European Software Engineering Conference and Symposium on the Foundations of Software Engineering. Virtual Event USA, 8–13 November 2020.

73. R. J. Williams, J. Peng, Function optimization using connectionist reinforcement learning algorithms. *Connect. Sci.* **3**, 241–270 (1991).

74. A. R. Mitchell, D. F. Griffiths, “Parabolic equations” in *The Finite Difference Method in Partial Differential Equations* (Wiley, 1980).

75. A. R. A. Anderson, “A hybrid discrete-continuum technique for individual-based migration models” in *Polymer and Cell Dynamics*, W. Alt, M. Chaplain, M. Griebel, J. Lenz, Eds. (Birkhäuser, Basel, 2003).

76. P. Pivonka, J. Zimak, D. W. Smith, B. S. Gardiner, C. R. Dunstan, N. A. Sims, T. John Martin, G. R. Mundy, Model structure and control of bone remodeling: A theoretical study. *Bone* **43**, 249–263 (2008).

77. X. Sun, J. Su, J. Bao, T. Peng, L. Zhang, Y. Zhang, Y. Yang, X. Zhou, Cytokine combination therapy prediction for bone remodeling in tissue engineering based on the intracellular signaling pathway. *Biomaterials* **33**, 8265–8276 (2012).

78. U. Alon, An Introduction to Systems Biology: Design Principles of Biological Circuits (Chapman & Hall/CRC, 2007), 320 pp.

79. X. Sun, J. Bao, K. C. Nelson, K. C. Li, G. Kulik, X. Zhou, Systems modeling of anti-apoptotic pathways in prostate cancer: Psychological stress triggers a synergism pattern switch in drug combination therapy. *PLoS Comput. Biol.* **9**, e1003358 (2013).

80. J. Xue, S. V. Schmidt, J. Sander, A. Draffehn, W. Krebs, I. Quester, D. De Nardo, T. D. Gohel, M. Emde, L. Schmidleithner, H. Ganesan, A. Nino-Castro, M. R. Mallmann, L. Labzin, H. Theis, M. Kraut, M. Beyer, E. Latz, T. C. Freeman, T. Ulas, J. L. Schultze, Transcriptome-based network analysis reveals a spectrum model of human macrophage activation. *Immunity* **40**, 274–288 (2014).

81. J. Zhang, J. J. Cunningham, J. S. Brown, R. A. Gatenby, Integrating evolutionary dynamics into treatment of metastatic castrate-resistant prostate cancer. *Nat. Commun.* **8**, 1816 (2017).

82. J. Zhang, J. Cunningham, J. Brown, R. Gatenby, Evolution-based mathematical models significantly prolong response to abiraterone in metastatic castrate-resistant prostate cancer and identify strategies to further improve outcomes. *eLife* **11**, e76284 (2022).

83. A. G. Baydin, B. A. Pearlmutter, A. A. Radul, J. M. Siskind, Automatic differentiation in machine learning: A survey. *J. Mach. Learn. Res.* **18**, 1–43 (2018).

84. C. J. C. H. Watkins, P. Dayan, Q-learning. *Mach. Learn.* **8**, 279–292 (1992).

85. R. J. Williams, Simple statistical gradient-following algorithms for connectionist reinforcement learning. *Mach. Learn.* **8**, 229–256 (1992).

86. V. R. Konda, J. N. Tsitsiklis, Actor-critic algorithms. *SIAM J. Control Optim.* **42**, 1143–1166 (2003).

87. R. S. Wong, Apoptosis in cancer: From pathogenesis to treatment. *J. Exp. Clin. Cancer Res.* **30**, 87 (2011).

88. G. van Loo, M. J. M. Bertrand, Death by TNF: A road to inflammation. *Nat. Rev. Immunol.* **23**, 289–303 (2023).

89. D. Bray, "Migration of cells over surfaces" in *Cell Movements: From Molecules to Motility* (Garland Science, ed. 2, 2000).

90. S. Goswami, E. Sahai, J. B. Wyckoff, M. Cammer, D. Cox, F. J. Pixley, E. R. Stanley, J. E. Segall, J. S. Condeelis, Macrophages promote the invasion of breast carcinoma cells via a colony-stimulating factor-1/epidermal growth factor paracrine loop. *Cancer Res.* **65**, 5278–5283 (2005).

91. O. Marescal, I. M. Cheeseman, Cellular mechanisms and regulation of quiescence. *Dev. Cell* **55**, 259–271 (2020).

92. X. P. Xie, D. R. Laks, D. Sun, M. Ganbold, Z. Wang, A. M. Pedraza, T. Bale, V. Tabar, C. Brennan, X. Zhou, L. F. Parada, Quiescent human glioblastoma cancer stem cells drive tumor initiation, expansion, and recurrence following chemotherapy. *Dev. Cell* **57**, 32–46.e8 (2022).

93. E. Eden, N. Geva-Zatorsky, I. Issaeva, A. Cohen, E. Dekel, T. Danon, L. Cohen, A. Mayo, U. Alon, Proteome half-life dynamics in living human cells. *Science* **331**, 764–768 (2011).

94. M. Laviron, A. Boissonnas, Ontogeny of tumor-associated macrophages. *Front. Immunol.* **10**, 1799 (2019).

95. E. Cendrowicz, Z. Sas, E. Bremer, T. P. Rygiel, The role of macrophages in cancer development and therapy. *Cancers* **13**, 1946 (2021).

96. S. J. Hao, Y. Wan, Y. Q. Xia, X. Zou, S. Y. Zheng, Size-based separation methods of circulating tumor cells. *Adv. Drug Deliv. Rev.* **125**, 3–20 (2018).

97. D. K. Wells, Y. Chuang, L. M. Knapp, D. Brockmann, W. L. Kath, J. N. Leonard, Spatial and functional heterogeneities shape collective behavior of tumor-immune networks. *PLoS Comput. Biol.* **11**, e1004181 (2015).

98. B. Szomolay, T. D. Eubank, R. D. Roberts, C. B. Marsh, A. Friedman, Modeling the inhibition of breast cancer growth by GM-CSF. *J. Theor. Biol.* **303**, 141–151 (2012).

99. D. Chen, J. M. Roda, C. B. Marsh, T. D. Eubank, A. Friedman, Hypoxia inducible factors-mediated inhibition of cancer by GM-CSF: A mathematical model. *Bull. Math. Biol.* **74**, 2752–2777 (2012).

100. C. Morgan, J. W. Pollard, E. R. Stanley, Isolation and characterization of a cloned growth factor dependent macrophage cell line, BAC1.2F5. *J. Cell Physiol.* **130**, 420–427 (1987).

101. A. Sorkin, J. E. Duex, Quantitative analysis of endocytosis and turnover of epidermal growth factor (EGF) and EGF receptor. *Curr. Protoc. Cell Biol.* doi: 10.1002/0471143030. cb1514s46 , (2010).

102. R. G. Thorne, S. Hrabetová, C. Nicholson, Diffusion of epidermal growth factor in rat brain extracellular space measured by integrative optical imaging. *J. Neurophysiol.* **92**, 3471–3481 (2004).

103. A. R. A. Anderson, A hybrid mathematical model of solid tumour invasion: The importance of cell adhesion. *Math. Med. Biol.* **22**, 163–186 (2005).

104. H. Knútsdóttir, E. Pálsson, L. Edelstein-Keshet, Mathematical model of macrophage-facilitated breast cancer cells invasion. *J. Theor. Biol.* **357**, 184–199 (2014).

105. A. R. A. Andersona, M. A. J. Chaplain, E. L. Newman, R. J. C. Steele, A. M. Thompson, Mathematical modelling of tumour invasion and metastasis. *J. Theor. Med.* **2**, 129–154 (2000).

106. C. Gong, O. Milberg, B. Wang, P. Vicini, R. Narwal, L. Roskos, A. S. Popel, A computational multiscale agent-based model for simulating spatio-temporal tumour immune response to PD1 and PDL1 inhibition. *J. R. Soc. Interface* **14**, 20170320 (2017).

107. H. Knutsdottir, J. S. Condeelis, E. Palsson, 3-D individual cell based computational modeling of tumor cell-macrophage paracrine signaling mediated by EGF and CSF-1 gradients. *Integr. Biol.* **8**, 104–119 (2016).

108. G. Mahlbacher, L. T. Curtis, J. Lowengrub, H. B. Frieboes, Mathematical modeling of tumor-associated macrophage interactions with the cancer microenvironment. *J. Immunother. Cancer* **6**, 10 (2018).

109. U. Del Monte, Does the cell number 10(9) still really fit one gram of tumor tissue? *Cell cycle* **8**, 505–506 (2009).
